# Selected cutaneous adverse events in patients treated with ICI monotherapy and combination therapy: a retrospective pharmacovigilance study and meta-analysis

**DOI:** 10.3389/fphar.2023.1076473

**Published:** 2023-06-02

**Authors:** Wenchao Lu, Huiyun Zhang, Qixiang Guo, Zhuoyue Gou, Jiannan Yao

**Affiliations:** ^1^ Department of Pharmacy, Beijing Chaoyang Hospital, Capital Medical University, Beijing, China; ^2^ Department of Oncology, Beijing Chaoyang Hospital, Capital Medical University, Beijing, China; ^3^ Institute for Drug Evaluation, Peking University Health Science Center, Beijing, China

**Keywords:** immune checkpoint inhibitors, cutaneous adverse events, FAERS analysis, meta-analysis, prevalence

## Abstract

**Introduction:** Cutaneous adverse events are commonly reported immune-related adverse events (irAEs), some of which are serious or even life-threatening, and it is essential to study these specific cutaneous AEs to understand their characteristics and risk.

**Methods:** We performed a meta-analysis of published clinical trials for immune checkpoint inhibitors (ICIs) to evaluate the incidence of cutaneous adverse events, using data from PubMed, Embase, and the Cochrane Library databases.

**Results:** A total of 232 trials with 45,472 patients were involved. Results showed that anti-PD-1 and targeted therapy combinations were associated with higher risk for most of the selected cutaneous adverse events. In addition, a retrospective pharmacovigilance study was conducted using the Food and Drug Administration (FDA) Adverse Events System database. Reporting odds ratio (ROR) and Bayesian information components (IC) were used to perform the disproportionality analysis. Cases were extracted from January 2011 to September 2020. We identified 381 (20.24%) maculopapular rash, 213 (11.32%) vitiligo, 215 (11.42%) Stevens‐Johnson syndrome (SJS), and 165 (8.77%) toxic epidermal necrolysis (TEN) cases. For vitiligo, anti-PD-1/L1 combined with anti-CTLA-4 therapy showed the strongest signal (ROR: 55.89; 95% CI: 42.34–73.78; IC_025_: 4.73). Palmar-plantar erythrodysesthesia (PPE) was reported with the most significant association with combined anti-PD-1/L1 and VEGF (R)-TKIs (ROR: 18.67; 95% CI: 14.77–23.60; IC_025_: 3.67). For SJS/TEN, antiPD-1 inhibitors showed the strongest signal (ROR: 3.07; 95% CI: 2.68–3.52; IC_025_: 1.39). The median onset time of vitiligo and SJS/TEN was 83 and 24 days, respectively.

**Conclusion:** Overall, in selected cutaneous AEs, each of them showed specific characteristics. It is necessary to realize their differences and take appropriate interventions in patients with different regimens.

## 1 Introduction

The use of immune checkpoint inhibitors (ICIs) has ushered in a new era of cancer treatment and achieved tremendous success in various cancer types (Postow et al., 2015). Although the performance of ICI monotherapy is encouraging, rational combination strategies are developed by physicians to enhance the antitumor effects of immunotherapies. Previous literature showed that combination therapy can synergistically reverse the immunosuppressive microenvironment and activate antitumor immunity (Fucà et al., 2018). Compared with conventional therapy, ICI combination therapies have shown longer progression-free and overall survival in randomized clinical trials, such as PD-1/L1 inhibitor plus CTLA-4 inhibitor for advanced melanoma, renal cell carcinoma, and colorectal cancer (Wolchok et al., 2017; Motzer et al., 2018; Overman et al., 2018); the PD-1/L1 inhibitor combination for renal cell carcinoma (Rini et al., 2019); and the PD-1/L1 inhibitor and cytotoxic chemotherapy combination for non-small-cell lung cancer and triple-negative breast cancer (Gandhi et al., 2018; Schmid et al., 2018).

It is well known that ICIs can cause a variety of immune-related adverse events (irAEs) (Postow et al., 2018), and the additive effects of combination therapies have raised concerns about severe or even life-threatening irAEs (Gao et al., 2019). Several meta-analyses (Da et al., 2019; Gu et al., 2019; Park et al., 2020) have shown that PD-1/L1 plus CTLA-4 inhibitor combinations have a significantly higher incidence of adverse events compared to PD-1/L1 inhibitor monotherapy, which highlights a significant challenge to the development of novel combinations based on PD-1/L1 inhibitors.

Among the various irAEs, cutaneous AEs are the most common AEs, especially maculopapular rash, pruritus, lichenoid eruptions, and vitiligo. Some of them are also associated with patient survival (Quaglino et al., 2010; Quach et al., 2021; Chen et al., 2022). The Stevens–Johnson syndrome (SJS) and toxic epidermal necrolysis (TEN) are rare but life-threatening cutaneous adverse reactions (Maloney et al., 2020). However, several cutaneous AEs are neither as frequent as rash nor as life-threatening as SJS/TEN, which may still lead to treatment withdrawal or even death. Nevertheless, there is limited evidence to compare the cutaneous AE risk of ICI monotherapy and combination therapies and explore the additive effects of combination therapies. Thus, it is important to recognize and manage these specific cutaneous AEs appropriately and maximize patient benefits if possible (Apalla et al., 2021). With early diagnosis and prompt management, patients may be able to continue ICI treatment, which may ultimately be crucial for overall treatment outcomes (Qiu et al., 2020).

Therefore, we performed a systematic review and meta-analysis of randomized controlled trials (RCTs) to estimate the risk of selected cutaneous AEs in different ICI regimens. However, some cutaneous AEs were rare, and the case numbers in the RCTs were limited; therefore, we also examined the selected cutaneous AEs of ICI-based regimens in clinical practice using real-world pharmacovigilance data from the Food and Drug Administration (FDA) Adverse Event Reporting System (FAERS), to characterize the spectrum, timing, and other clinical features.

The cutaneous adverse events included in clinical trials of the meta-analysis were identified according to the Common Terminology Criteria for Adverse Events 5.0 (CTCAE 5.0). The AEs reported in FAERS were based on the Medical Dictionary for Regulatory Activities (MedDRA). In the FAERS database, adverse events were reported by clinicians, pharmacists, pharmaceutical manufacturers, and even patients, which implies that the diagnosis and definition of cutaneous irAEs may be subjected to inconsistencies compared to professional dermatologic assessment.

## 2 Methods

### 2.1 Pharmacovigilance study

#### 2.1.1 Data source

FAERS, one of the world’s largest spontaneous reporting systems of drug adverse events (AEs), is designed to support the FDA post-marketing safety surveillance program (Poluzzi et al., 2012). FAERS contains 20 million AE reports (through December 2020) submitted by health professionals, consumers, and manufacturers. Data were extracted from reports registered between January 2011 and September 2020 in FAERS, and duplicate reports were excluded from our analysis. In the deduplication process, we extracted the most recent case version from all available cases based on the case ID, initial/follow-up code (“I” or “F”) of the case event, age, sex, and reporting country, and we retained the most current case version and removed all others (Banda et al., 2016).

#### 2.1.2 Search strategy

We identified the study drugs using both generic and trade names in FAERS. Adverse events in FAERS are coded using preferred terms (PTs) according to the Medical Dictionary of Regulatory Activities (MedDRA). We searched for the selected cutaneous toxicities using all PTs listed in Supplementary Table S1.

#### 2.1.3 Data analysis

Descriptive analysis was used to present the clinical features, including demographic information (age, gender, and reporter type), seriousness (i.e., resulting in death, initial or prolonged hospitalization, life-threatening events, leading to disability, congenital anomalies, and other serious conditions), therapeutic regimen, and indication. Furthermore, we accessed the “time to onset” data, which was defined as the interval between the reported “event date” and “therapy start time” of the administration of ICIs (Sakaeda et al., 2013).

Two data mining methods, reporting odds ratio (ROR) and Bayesian information components (IC), were used to perform disproportionality analysis. For ROR, the result was considered a signal if the lower limit of the 95% CI of ROR exceeded 1. For IC, a significant signal is indicated if the lower limit of 95% confidence interval for the IC (IC_025_) was above 0 (Almenoff et al., 2007). Data analysis was performed using RStudio, version 1.3.1093 (RStudio Inc., Boston, MA).

### 2.2 Meta-analysis

PubMed, Embase, and the Cochrane Library database were systematically searched for relevant English language articles published by 20 October 2020. The following terms were used (Nivolumab or Opdivo or Pembrolizumab or Lambrolizumab or Keytruda or Cemiplimab or Pidilizumab or Camrelizumab or SHR-1210 or JS001 or Sintilimab or Durvalumab or MEDI4736 or atezolizumab or Avelumab or Bavencio or Tremelimumab or Ticilimumab or Ipilimumab) and (Carcinoma or Neoplasia or Tumor or Cancer or Malignancy). Studies that met the following criteria were included: 1) studies included ICI monotherapy or ICI combination therapy with chemotherapy/targeted therapy/ICI in patients diagnosed with malignancies; 2) studies investigating the following cutaneous adverse events: vitiligo, bullous dermatitis, palmar-plantar erythrodysesthesia (PPE) syndrome, maculopapular rash, drug eruption, erythema multiform, acneiform rash, skin exfoliation, skin ulceration, urticarial, SJS, and TEN; and 3) randomized controlled clinical trials or single/multicenter research. When more than one publication reported the same trial, the article with the longer follow-up time was selected. We also searched The Clinical Trial website by the NCT number of each article for more detailed information. Two reviewers independently screened the titles and abstracts of the retrieved citations. Any discrepancies were resolved by discussion. The extracted data included PubMed ID, tumor type, treatment regimen, the dose of ICIs, the total number of patients treated, and the number and type of cutaneous drug-related events. The proportion of selected cutaneous AEs and the 95% CI of each ICI treatment regimen were evaluated. This meta-analysis was conducted using R statistical software (packages metafor and Rstudio). The fixed- and random-effects models were used to estimate event rates and their corresponding 95% confidence intervals. Forest plots were generated to summarize the results of each analysis group in proportion and provide a visual analysis of studies evaluating selected cutaneous AEs.

## 3 Results

### 3.1 Pharmacovigilance study

A total of 96,802 adverse events were reported for approved ICIs in the FAERS database from Q1/2011 to Q3/2020, of which 1,882 were cutaneous adverse events related to ICIs. We summarized the basic demographic and clinical characteristics of these patients in Table 1. As for the reporter category, healthcare professionals reported 74.39% of cases. The median age of affected patients was 66.0 years (IQR: 57.0–73.0; data available: 1,518/1,882 cases). Male patients accounted for 56.96%, and female patients accounted for 36.34%. Among these cases, 918 (48.78%) were associated with anti-PD-1 monotherapy, 340 (18.07%) were associated with anti-PD-1/L1 combined with anti-CTLA-4 therapy, and 256 (13.60%) were associated with combined chemotherapy with anti-PD-1/L1 (Table 2). Cutaneous AEs were most prevalent in patients with melanoma (31.35%), followed by lung cancer (30.34%) and genitourinary cancer (12.86%). Death outcomes occurred in 326 (17.32%) cases.

**TABLE 1:** Characteristics of patients with ICI-associated skin diseases sourced from the FAERS database (1 January 2011–31 September 2020).

Characteristic	*n* (%)
Total case	1,882
Reporters	
Healthcare professional	1,400 (74.39%)
Non-healthcare professional	471 (25.03%)
Unknown or missing	11 (0.58%)
Sex	
Male	1,072 (56.96%)
Female	684 (36.34%)
Unknown or missing	126 (6.70%)
Age	
Total data	1,518 (80.66%)
Median (years)	66
IQR (years)	57–73
Range (years)	10–96
Outcome	
Death	326 (17.32%)
Life-threatening	88 (4.68%)
Disability	43 (2.28%)
Hospitalization	709 (37.67%)
Other serious outcome	510 (27.10%)
Unknown or missing	206 (10.95%)
Indication	
Melanoma	590 (31.35%)
Lung cancer	571 (30.34%)
Genitourinary cancer	242 (12.86%)
Gastrointestinal cancer	80 (4.25%)
Head and neck cancer	55 (2.92%)
Gynecologic cancer	45 (2.39%)
Hematological cancer and lymphoma	44 (2.34%)
Other cancer	55 (2.92%)
Non-specified cancer	47 (2.50%)
Unknown or missing	153 (8.13%)
Reaction	
Vitiligo	213 (11.32%)
Palmar-plantar erythrodysesthesia	125 (6.64%)
Dermatitis bullous	72 (3.83%)
Drug eruption	187 (9.94%)
Erythema multiforme	149 (7.92%)
Dermatitis acneiform	52 (2.76%)
Skin exfoliation	129 (6.85%)
Maculopapular rash	381 (20.24%)
Skin ulcer	92 (4.89%)
Urticaria	226 (12.01%)
Stevens–Johnson syndrome	215 (11.42%)
Toxic epidermal necrolysis	165 (8.77%)
Time to occur	
Total data	822 (43.68%)
Median (days)	27
IQR (days)	10-77
Regimen	
Anti-PD-1	918 (48.78%)
Anti-PD-L1	87 (4.62%)
Anti-CTLA-4	130 (6.91%)
Anti-PD-1/L1 + CTLA-4	340 (18.07%)
Anti-PD-1/L1 + chemotherapy	256 (13.60%)
Anti-CTLA-4 + chemotherapy	6 (0.32%)
Anti-PD-1/L1 + EGFR-TKI	8 (0.43%)
Anti-PD-1/L1 + EGFR-MA	1 (0.05%)
Anti-PD-1/L1 + VEGF(R)-TKI	131 (6.96%)
Anti-PD-1/L1 + VEGF(R)-MA	5 (0.27%)

ICI, immune checkpoint inhibitor; IQR, interquartile range; PD-1, programmed cell death-1; PD-L1, programmed cell death-ligand 1; CTLA-4, cytotoxic T lymphocyte-associated antigen-4; EGFR, epidermal growth factor receptor; VEGF(R), vascular endothelial growth factor (receptor); TKIs, tyrosine kinase inhibitors; MA, monoclonal antibody.

**TABLE 2:** Associations of ICI-related regimens with specific skin diseases.

Category	N. of cases	ROR	ROR_025_	ROR_975_	IC	IC_025_
Total	1,882	1.12	**1.07**	1.17	0.16	**0.09**
Anti-PD-1	918	1.05	0.99	1.12	0.07	−0.02
Anti-PD-L1	87	0.63	0.51	0.78	−0.65	−0.96
Anti-CTLA-4	130	0.77	0.65	0.92	−0.37	−0.63
Anti-PD-1/L1 + CTLA-4	340	1.72	**1.54**	1.92	0.76	**0.60**
Anti-PD-1/L1 + chemotherapy	256	1.36	**1.20**	1.54	0.43	**0.25**
Anti-CTLA-4 + chemotherapy	6	0.66	0.30	1.48	−0.66	−1.84
Anti-PD-1/L1 + EGFR-TKI	8	2.01	0.99	4.08	0.82	−0.22
Anti-PD-1/L1 + EGFR-MA	1	−	−	−	−	−
Anti-PD-1/L1 + VEGF(R)-TKI	131	2.14	**1.80**	2.55	1.06	**0.80**
Anti-PD-1/L1 + VEGF(R)-MA	5	0.40	0.17	0.96	−1.37	−2.66

ICI, immune checkpoint inhibitor; PD-1, programmed cell death-1; PD-L1, programmed cell death-ligand 1; CTLA-4, cytotoxic T lymphocyte-associated antigen-4; EGFR, epidermal growth factor receptor; VEGF(R), vascular endothelial growth factor (receptor); TKIs, tyrosine kinase inhibitors; MA, monoclonal antibody; ROR, reporting odds ratio; ROR_025_, the lower limit of the 95% confidence interval of ROR; IC, information components; IC_025_, the lower limit of the 95% confidence interval of IC.

Bold values showed the significant signal of the specific irAEs.

**FIGURE 1:**
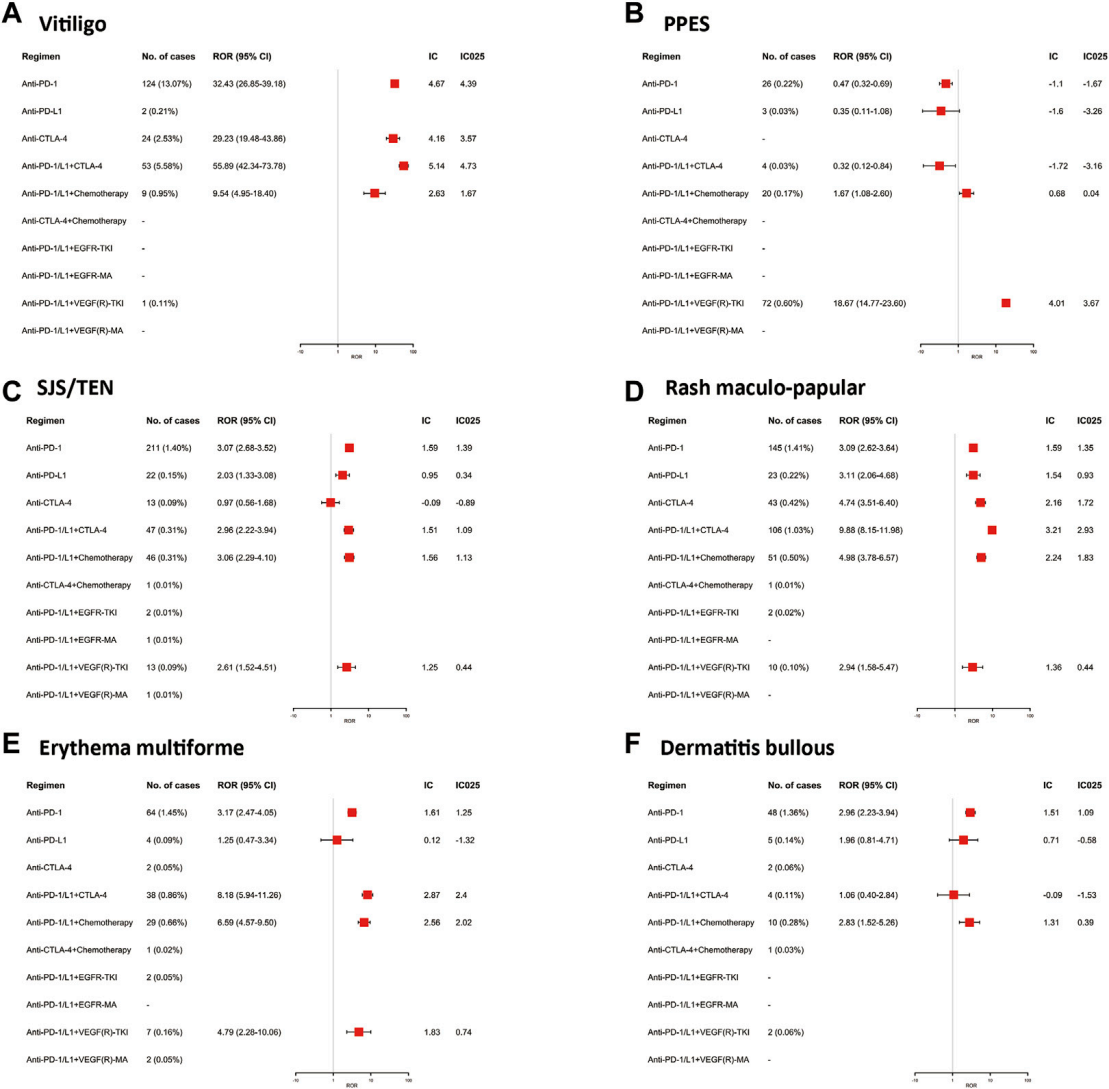
The Associations of different ICI-related regimens with specific skin diseases in the disproportionality analysis in FAERS.

**FIGURE 2:**
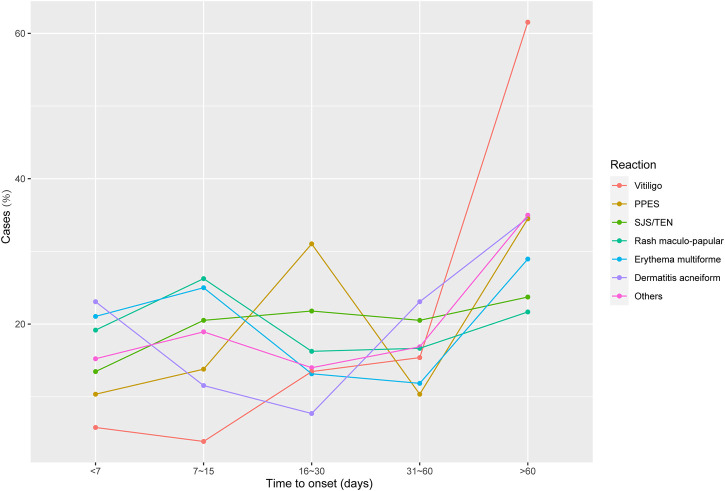
Time from drug initiation to adverse drug reaction onset, in days; PPES, palmar-plantar erythrodysesthesia syndrome; SJS/TEN, Stevens–Johnson syndrome/toxic epidermal necrolysis.

To provide detailed information on the specific cutaneous AEs associated with different ICI treatment regimens, we assessed the signal of each selected cutaneous adverse event. In the available data, all ICI-related regimens had significant vitiligo signals, and anti-PD-1/L1 combined with anti-CTLA-4 therapy showed the strongest signal (ROR: 55.89; 95% CI: 42.34–73.78; IC_025_: 4.73). ICI monotherapy, PD-1 inhibitors, or CTLA-4 inhibitors also had very significant associations with vitiligo, with RORs of 32.43 (26.85–39.18) and 29.23 (19.48–43.86), respectively. The time onset analysis (Figure 2) indicated that vitiligo occurred later than other skin AEs, with a median onset of approximately 3 months (83.0, IQR: 36.5–158.3 days). Notably, in our FAERS analysis, PPE showed the highest signal in anti-PD-1/L1 combined with VEGF(R)-TKIs, with ROR of 18.67 (14.77–23.60) and IC_025_ of 3.67. Anti-PD-1/L1 combined chemotherapy showed a weak signal (ROR: 1.67; 95% CI: 1.08–2.60; IC_025_: 0.04), and other regimens had no significant signals. SJS/TEN are rare but life-threatening adverse events. In this FAERS study, the majority of SJS/TEN cases were reported in patients receiving anti-PD-1 inhibitors (211, 1.40%; ROR: 3.07; 95% CI: 2.68–3.52; IC_025_: 1.39), followed by anti-PD-1/L1 combined chemotherapy (ROR: 3.06; 95% CI: 2.29–4.10; IC_025_: 1.13) and anti-PD-1/L1 combined with anti-CTLA-4 therapy (ROR: 2.96; 95% CI: 2.22–3.94; IC_025_: 1.09). Furthermore, SJS/TEN presented a short time-to-onset of approximately 1 month (24.0; IQR: 10.3–69.3 days). Maculopapular rash was the most common cutaneous AE, which accounted for 20.24% of selected cutaneous AEs and could be found in all the regimens, especially anti-PD-1/L1 combined anti-CTLA-4 therapy (ROR: 9.88; 95% CI: 8.15–11.98; IC_025_: 2.93). The rest of the results are shown in Figures 1, 2.

### 3.2 Meta-analysis

**FIGURE 3:**
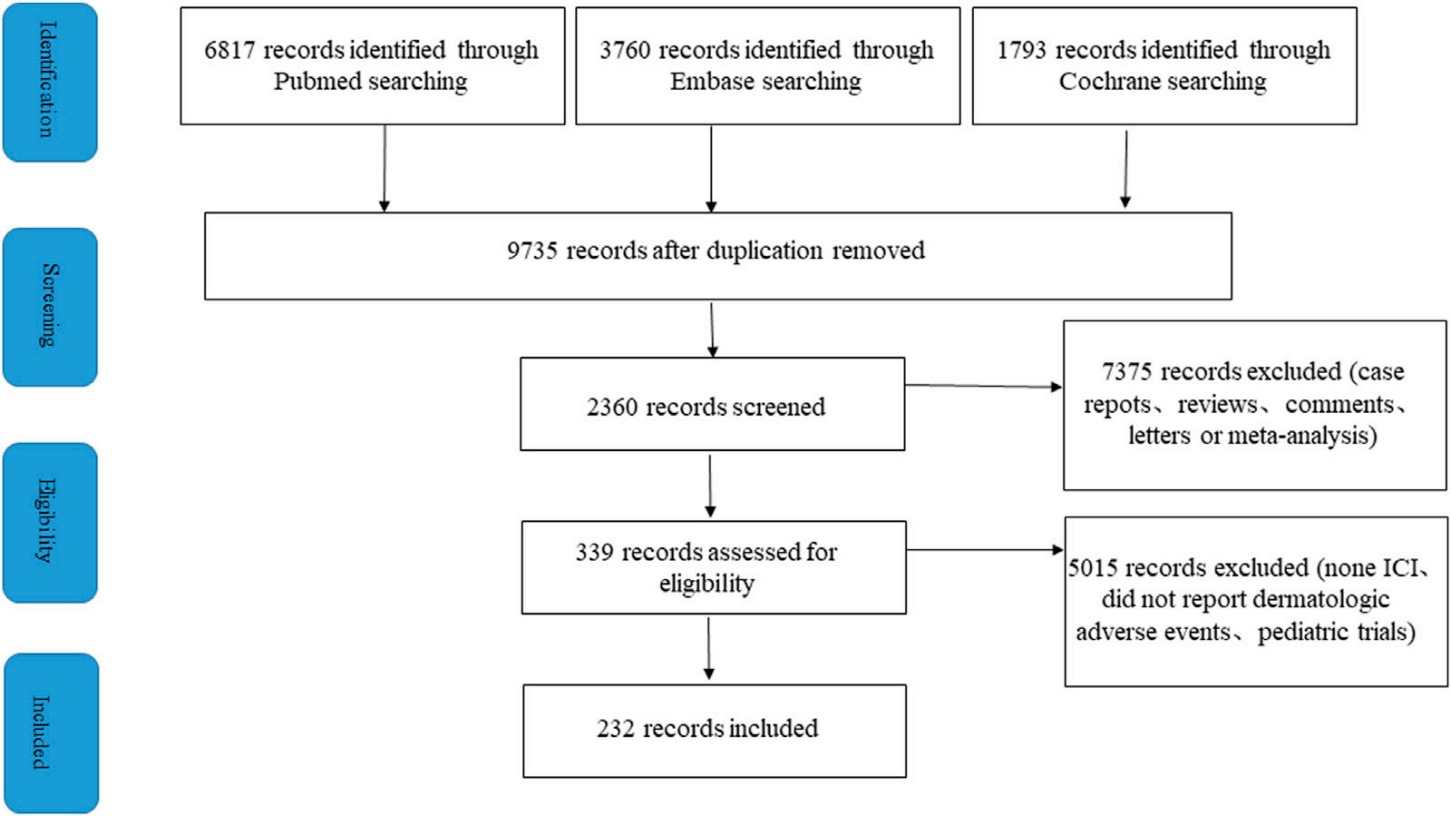
PRISMA flowchart of literature search and study selection.

With only the findings provided by FAERS, it is difficult to define the frequency of selected cutaneous AEs in individual ICI regimens. To estimate their frequency, we evaluated all published clinical trials and studies of anti-PD-1, anti-PD-L1, anti-CTLA-4, and their combination therapies, including chemotherapy and EGF/VEGF targeted therapy. A total of 232 trials with 45,472 patients were identified (Figure 3). Details of cutaneous adverse events identified following different ICI treatment regimens are shown in Supplementary Appendix S5.

In ICI monotherapy, anti-PD-1 therapy was associated with selected cutaneous AEs more frequently than anti-PD-L1 and anti-CTLA-4 therapy (anti-PD-1 therapy: 2.54%, 95% CI: 1.38%–3.93%, I2: 94%, *p* < 0.01; anti-PD-L1 therapy: 0.57%, 95% CI: 0.05%–1.45%, I2: 82%, *p* < 0.01; anti-CTLA-4 therapy: 1.40%, 95% CI: 0.23%–3.18%, I2: 92%, *p* < 0.01). For anti-PD-1/L1 and anti-CTLA-4 combination therapy, the proportion of selected cutaneous AEs was 9.98%, with 95% CI of 6.08%–14.58%, I2 of 94%, and *p* < 0.01. When combined with chemotherapy, anti-PD-1/L1 therapy (3.79%; 95% CI: 1.62%–6.65%; I2: 952%; *p* < 0.01) was estimated to have more frequent cutaneous AEs relative to anti-CTLA-4 therapy (1.91%; 95% CI: 0.00%–5.91%; I2: 83%; *p* < 0.01). The proportion of anti-PD-1/L1 therapy combined with EGF-targeted therapy was 21.01%, with 95% CI of 4.01%–44.82%, I2 of 94%, and *p* < 0.01. As the mechanisms of EGFR TKI and EGF monoclonal antibody are not the same, we evaluated the proportion of cutaneous AEs of both types of treatment. According to our results, the frequency of cutaneous AEs in anti-PD-1/L1 therapy and EGFR TKI combination therapy (22.44%; 95% CI: 0.43%–57.91%; I2: 90%, *p* < 0.01) was similar to anti-PD-1/L1 and EGFR monoclonal antibody therapy (23.17%; 95% CI: 4.78%–41.56%; I2: 97%; *p* < 0.01). The proportion of cutaneous AEs with anti-PD-1/L1 and VEGF targeted therapy was 20.63%, with 95% CI of 13.57%–28.65%, I2 of 96%, and *p* < 0.01. Regarding VEGFR TKI and VEGF monoclonal antibody, the results revealed that VEGFR TKI (41.20%; 95% CI: 33.17%–49.72%; I2: 88%; *p* < 0.01) was estimated to have more frequent cutaneous AEs compared to VEGF monoclonal antibody (4.23%; 95% CI: 1.09%–8.79%; I2: 88%; *p* < 0.01) when combined with anti-PD-1/L1 therapy (Supplementary Appendix S3).

To provide additional information on the different spectra of selected cutaneous adverse events with the ICI treatment regimen, we evaluated the proportion of each selected cutaneous adverse event. Similar to data in the FAERS database, maculopapular rash was the most common cutaneous adverse event with both ICI monotherapy and combination therapy. Vitiligo and PPES were suggested to be relatively common in ICI combination therapy. It should be noted that the occurrence of PPES for PD-1/L1 and VEGFR targeted therapy combination therapy was 33.36%, with 95% CI of 24.04%–42.68%, I2 of 96%, and *p* < 0.01, which made it the most frequent cutaneous adverse event in this treatment regimen. Unlike the FAERS database, the rest of the cutaneous adverse events evaluated were rare, especially SJS and TEN (Supplementary Appendix S4). The proportions for each selected cutaneous adverse event associated with different treatment regimens are summarized in Supplementary Table S1.

### 3.3 Case report

As the oncology department of a general hospital during the COVID-19 outbreak in Beijing, we were responsible for treating nearby residents diagnosed with solid tumors. We have been paying close attention to the cutaneous adverse events of ICIs in our department since the widespread application of immunotherapy. A renal cancer patient with severe cutaneous and mucosal side effects after the application of anti-PD-1 combined with VEGFR TKI came to our hospital and attracted our attention.

On 30 March 2021, a 70-year-old man was admitted to the ER of our hospital for having oral mucosa ulcers and rash for more than 2 weeks, which had become progressively exacerbated. He had a personal history of left kidney malignancy (lung metastasis) and had been treated with axitinib (5 mg twice a day for 6 months) and tislelizumab (200 mg every 3 weeks for four cycles). No personal or family history of drug allergies or autoimmune diseases was reported. After the dermatology consultation, a physical examination revealed large areas of oral ulcers, bleeding, and scabs; scattered erythema of different sizes on the trunk and limbs; white scales attached to the surface; easy to scrape, visible film phenomenon; and scattered edema, erythema, and miliary-sized blisters on the hands and palms, some of which were accompanied by blood blisters. Scabs were formed, and large areas of erythema, papules, and desquamation could be seen on the forearm, scrotum, and perianal area, accounting for more than 30% of the body surface area (Supplementary Figure A). Blood tests showed a high erythrocyte sedimentation rate (ESR) of 48 mm/h (2–15), C-reactive protein (CRP) of 5.10 mg/dL (0–0.8), and immunoglobulin E (IgE) of 1,030.00 IU/ml (0–100). Microscopic analysis showed five erythrocytes for each HPF. A histological examination could not be performed because the patient was from the COVID-19 high-risk area and had to be isolated in a special ward. We consulted an experienced dermatologist. Based on his medical history and clinical presentation, the diagnosis of this case was a drug-related cutaneous side effect, SJS (grade 3). Treatment included methylprednisolone 60 mg intravenous injection each day (1 mg/kg), intravenous nutritional support, prophylactic antibiotics, and monitoring blood sugar and food intake. The topical drugs used included human granulocyte-stimulating factor and lidocaine mouthwash three times a day before meals, halometasone and urea cream mixed externally used for trunk and extremities twice a day, triamcinolone acetonide and econazole nitrate cream, and mupirocin and zinc oxide externally used for perineum and perianal regions twice a day. Five days later, his oral mucosa ulcers and rash improved, and he could eat orally. Methylprednisolone was adjusted to 40 mg intravenous injection each day for another 3 days. Blood tests were repeated, his CRP and ESR returned to normal, and IgE dropped to 907 IU/ml. After 8 days of methylprednisolone and other supportive care, the patient’s symptoms were significantly controlled (Supplementary Figure A), and he was prescribed prednisone acetate 50 mg per day and discharged from the hospital.

## 4 Discussion

To the best of our knowledge, the present study is the largest and most extensive analysis of cutaneous adverse events associated with ICIs using data from both clinical trials and a worldwide pharmacovigilance database. In our study, the systematic review and meta-analysis included 232 studies of combination therapies based on PD-1 and PD-L1 inhibitors and 1,882 cases related to ICI-based regimen-induced cutaneous adverse events from the pharmacovigilance data. Based on a thorough review of current studies, we surmised that PD-1 or anti-PD-L1 inhibitors are mainly combined with immunotherapy, chemotherapy, and VEGF(R) inhibitors, and identified 10 cutaneous adverse events: vitiligo, bullous dermatitis, PPE syndrome, maculopapular rash, drug eruption, erythema multiform, acneiform rash, cutaneous exfoliation, cutaneous ulceration, urticarial, SJS, and TEN.

### 4.1 The overview of the result of this study

In this study, among the PD-1 and PD-L1 inhibitor-based combinations and monotherapy, anti-PD-1/L1 combined with VEGF(R) inhibitor showed the highest risk of selected cutaneous adverse events, which was identified in meta-analysis and FAERS analysis. However, in terms of VEGF(R) combinations, the incidence of cutaneous AEs for VEGF(R)-TKI or VEGF(R)-mAbs showed a wide variation, with 41.20% for VEGF(R)-TKI and 4.23% for VEGF(R)-mab, and our pharmacovigilance analysis study also confirmed the difference. Furthermore, previous studies showed that severe AEs associated with ICIs plus VEGF(R)-mAbs were lower than those of ICIs plus VEGF(R)-TKIs (Ara and Pastushenko, 2014; Gao et al., 2019). As for EGFR combination immunotherapy, meta-analysis also showed a high incidence of cutaneous toxicities. However, the results were similar between EGFR-TKIs and EGFR-mAbs. Consistent with previous studies (Le et al., 2022; Wongvibulsin et al., 2022), both meta-analysis and FAERS analysis showed that anti-CTLA-4 combined with anti-PD/L-1 exhibited a much higher risk than monotherapy for most cutaneous adverse events, such as vitiligo, maculopapular rash, and erythema multiforme. However, as for SJS/TEN, anti-PD-1 monotherapy appeared with a higher risk, which was consistent with a previous pharmacovigilance study (Zhu et al., 2021). Consistent with the previous literature (Le et al., 2022; Wongvibulsin et al., 2022), our analysis also found that patients receiving anti-CTLA-4 monotherapy were less likely to develop the selected cutaneous adverse events compared to PD-1 monotherapy, such as vitiligo, PPE, and SJS/TEN. Notably, as for anti-PD/L-1 combined VEGF(R) inhibitors, PPE showed the highest risk among the selected cutaneous adverse events. This result corroborated the findings of previous work (Ara and Pastushenko, 2014; Ishak et al., 2014). The number of maculopapular rash cases accounted for the largest proportion in our study, which represented the most common cutaneous irAEs observed with PD-1/PD-L1- and CTLA-4-related regimens (Geisler et al., 2020).

### 4.2 ICI combination therapy with VEGF/EGF targeted therapy

Previous research has pointed out that ICI combination strategies inducing vascular normalization may resume immune cell functions and contribute to the attenuation of immunosuppression caused by the tumor microenvironment (TME), thereby improving the activity of immunotherapy (Gao et al., 2019). Such a combination strategy has been approved worldwide to be utilized among different tumors, such as liver and kidney cancers. The current studies suggest that anti-PD-1/L1 therapy and VEGF-targeted therapy may cause increased cutaneous AEs, but the spectrum and characteristics of their combinations remain unclear. As mentioned previously, our results demonstrated that anti-PD-1/L1 combined with VEGF(R) inhibitor was associated with the most frequently selected cutaneous AEs among different ICI treatment regiments. Detailed data suggested that PPE represented a large proportion of identified skin AEs associated with combined anti-PD-1/L1 and VEGF(R) inhibitors, and most of them were the anti-PD-1/L1 and sunitinib/axitinib combination. The most commonly reported cutaneous AEs associated with anti-PD-1/L1 therapy include psoriasiform, eczematous, lichenoid, and morbilliform drug eruptions (Chen et al., 2022). PPE was observed in 48% of patients treated with sorafenib and 36% of those treated with sunitinib (Lee et al., 2009). The exact mechanism of PPE pathogenesis remained unknown. It has been hypothesized that cytotoxic agents may be excreted in sweat, making palms and soles more prone to HFSR due to the increased numbers of eccrine sweat glands in the extremities (Lee et al., 2009). Moreover, the inhibition of VEGF(R) could hypothetically affect the vascular repair mechanisms in the body, resulting in marked inflammation after any vascular damage, and this effect could be highlighted in areas with high-pressure and repeated subclinical trauma, such as on the palms and soles (Ishak et al., 2014). Axitinib was launched later than sorafenib and sunitinib, and there is a lack of detailed investigation of relative cutaneous AEs, but all three are VEGF inhibitors. Therefore, axitinib may share the same mechanism of PPE pathogenesis. Furthermore, our results revealed that VEGF-mAbs were associated with fewer cutaneous AEs compared to VEGFR-TKI, which was consistent with previous studies (Ishak et al., 2014). In our detailed data, PPE still accounted for the majority of reported cutaneous AEs among anti-PD-1/L1 and VEGF-mAbs combinations, and most were bevacizumab-related. Few cutaneous AEs were reported among the combination of anti-PD-1/L1 and ranibizumab, which may be because the application of ranibizumab is not yet widespread. Our meta-analysis also suggested that combined anti-PD-1/L1 and EGFR inhibitors were associated with higher cutaneous AEs, and there was no significant difference between EGFR monoclonal antibodies and TKIs. However, this result was not verified in FAERS due to insufficient data. At present, combination therapy with ICIs and EGFR inhibitors has not been widely applied, but relevant clinical trials are investigating these strategies. EGFR-TKI monotherapy has been utilized for EGFR mutant lung cancer, and EGF monoclonal antibodies, together with chemotherapy, have been used for breast cancer, gastric cancer, and colon cancer, among others. Cutaneous AEs are common side effects of EGFR inhibitors because EGFR is expressed in the basal layer of epidermal keratinocytes and in hair follicles, where it plays a key role in regulating cell proliferation and differentiation (Gisondi et al., 2021). Maculopapular rash accounted for the majority of reported cutaneous AEs among anti-PD-1/L1 and EGFR inhibitor combinations, according to our results. Other selected cutaneous AEs, such as erythema multiform, maculopapular rash, and skin exfoliation, were more frequent among anti-PD-1/L1 plus VEGFR/EGFR inhibitor combination therapy than other ICI combination regimens and required more attention from oncologists and dermatologists.

### 4.3 ICI combination therapy with chemotherapy and other ICIs

To date, ICIs combined with chemotherapy have been approved regimens in numerous indications and have been particularly successful in the treatment of non-small cell lung cancer (NSCLC) (Yap et al., 2021). Our results suggest that anti-PD-1/L1 combined chemotherapy is associated with increased cutaneous AEs compared with anti-PD-1/L1 monotherapy, but anti-CTLA-4 combined chemotherapy did not significantly increase cutaneous AEs compared with anti-CTLA-4 monotherapy. This result may be due to the higher toxicity of CTLA-4, and the maintenance cycle of anti-CTLA-4 plus chemotherapy in the RCT design is generally less than that of anti-PD-1/L1 plus chemotherapy (Yamazaki et al., 2015). Conversely, anti-PD-1/PD-L1 plus anti-CTLA-4 therapy is associated with increased selected cutaneous AEs compared with either anti-PD-1/L1 or anti-CTLA-4 monotherapy. According to detailed meta-analysis data, maculopapular rash accounted for the majority of selected cutaneous AEs among anti-PD-1/PD-L1 plus anti-CTLA-4 therapy, which was one of the most frequent cutaneous irAEs observed with ICIs (Sibaud, 2018).

### 4.4 Case report

We reported a case of a 70-year-old man with kidney malignancy who developed SJS after receiving the combination therapy of axitinib (anti-VEGFR TKI) and tislelizumab (anti-PD-1 monoclonal antibody). For the treatment of SJS, in addition to supportive care (maintaining body fluids and electrolyte balance, nutritional support, and skin/oral mucosal wound care to prevent infection), the use of systemic steroids remains controversial. Short-term, high-dose corticosteroids are believed to be helpful for patients’ prognoses (Charlton et al., 2020). We immediately administered intravenous corticosteroids to this patient as we believed that the patient’s symptoms were secondary to the combination of ICI and anti-VEGF therapy. In addition to providing supportive care and discontinuing the use of the relevant medications as soon as possible, it is necessary to inhibit inflammation. SJS has a reported mortality rate of 30%. Therefore, timely and appropriate treatment is essential. Whether the patient should continue treatment with ICI/VEGF after full recovery needs further discussion. This case reminded us that in addition to appropriate treatment, early detection and intervention of cutaneous AEs are also very important for patient outcomes. Therefore, it is necessary to investigate the characteristics and spectrums of cutaneous AEs of different ICI treatment regimens and educate oncologists, dermatologists, and emergency physicians.

### 4.5 Limitations

Our results suffered from confounders and limitations. First, we included an expert dermatologist in our data analysis process. However, the data source in the FAERS database and meta-analysis could be reported by different clinicians, pharmacists, or even pharmaceutical manufacturers. Thus, it is difficult to control for heterogeneity in the diagnosis and definition of cutaneous irAEs, compared to professional dermatologic diagnosis. Notably, SJS, TEN, EM, and bullous pemphigoid are difficult to distinguish clinically. Second, most AEs are time-dependent, so the incidence is associated with the length of time on treatment. However, due to severe toxicity, anti-CTLA4 therapies are used for a very limited time, whereas anti-PD1 therapies are used for a longer time, which may affect the incidence of AEs in the meta-analysis. Third, meta-analysis is based on clinical trials that have strict inclusion criteria. Most patients were in good health condition prior to treatment, which means fewer comorbidities and fewer concomitant drugs, making the occurrence of adverse events underestimated compared to real-world data. Finally, cases are voluntarily reported to the FAERS database. Therefore, the relationship between the target drugs and suspected adverse events may be affected by other biases, and further studies are needed.

## 5 Conclusion

For most selected cutaneous AEs, compared with either anti-PD-1/L1 or CTLA-4 monotherapy, ICI-based combination therapies were associated with more frequent cutaneous AEs. For example, vitiligo showed the strongest signal in anti-PD-1/L1 combined with anti-CTLA-4 therapy, and PPE was reported to have the most significant association with anti-PD-1/L1 combined with VEGF(R)-TKIs. However, SJS/TEN was found to be the strongest signal in anti-PD-1 inhibitor monotherapy rather than combination therapies. In addition, the time to the appearance of these cutaneous AEs is highly variable, such as vitiligo and SJS/TEN. Therefore, it is important to pay more attention to these cutaneous toxicities, realize their specific characteristics, and appropriately intervene in patients prescribed different regimens.

## Novelty and impact

We selected some specific cutaneous AEs to compare their risks and clinical features in ICI monotherapy and ICI-based combination therapies. For most cutaneous AEs, ICI-based combination therapies were associated with more frequent reports than ICI monotherapy. Vitiligo showed the strongest signal in anti-PD-1/L1 combined anti-CTLA-4 therapy. Palmar-plantar erythrodysesthesia (PPE) had the most significant association with anti-PD-1/L1 combined with VEGF(R)-TKIs. Furthermore, SJS/TEN had the strongest association with anti-PD-1 inhibitor monotherapy. The time to onset of these cutaneous AEs showed wide differences. These findings suggest it is important to pay more attention to these cutaneous toxicities and make appropriate interventions in different regimens.

## Data Availability

The original contributions presented in the study are included in the article/Supplementary Material. Further inquiries can be directed to the corresponding author.
