# Supplementary material for: Selected cutaneous adverse events in patients treated with ICI monotherapy and combination therapy: a retrospective pharmacovigilance study and meta-analysis
Source: Front Pharmacol. 2023 Jun 2;14:1076473. doi: 10.3389/fphar.2023.1076473 (PMC10272362; doi:10.3389/fphar.2023.1076473)

Figure 1 Forest plot of proportion of selected cutaneous adverse events with anti-PD-1 therapy


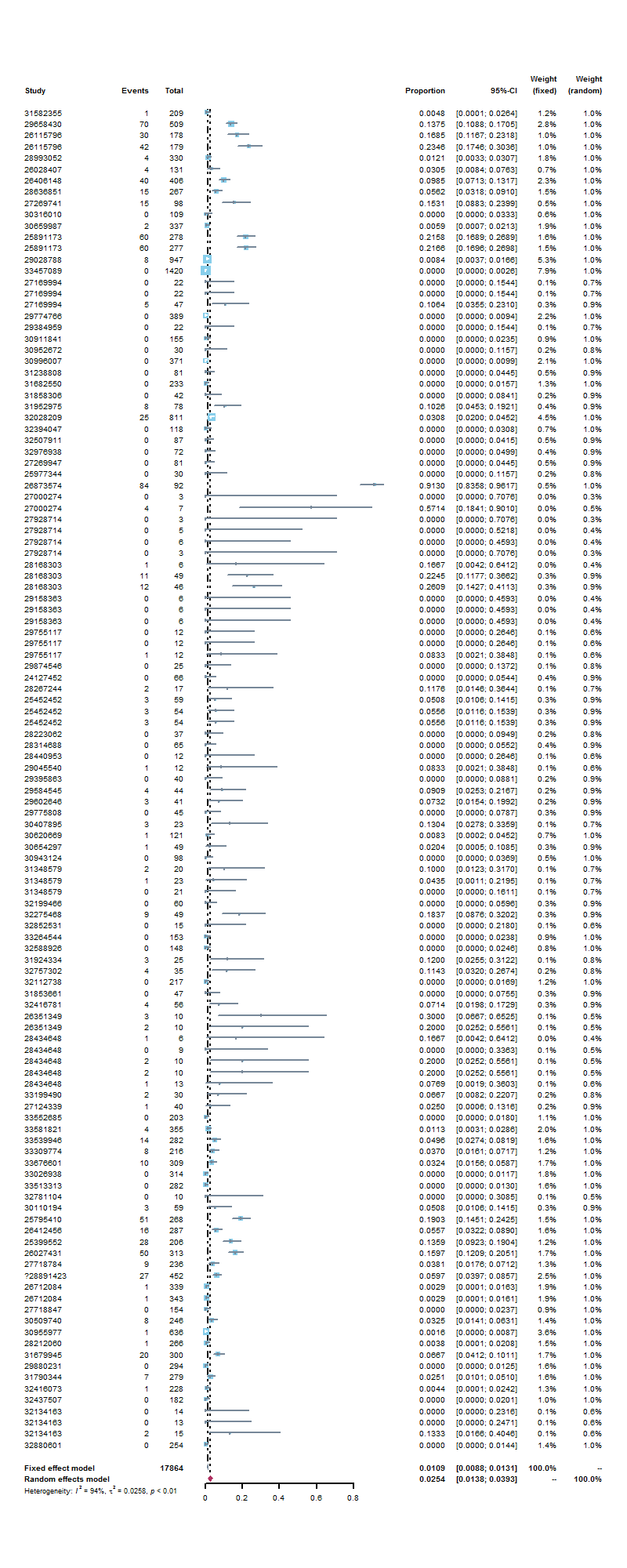


Figure 2 Forest plot of proportion of selected cutaneous adverse events with anti-PD-L1 therapy


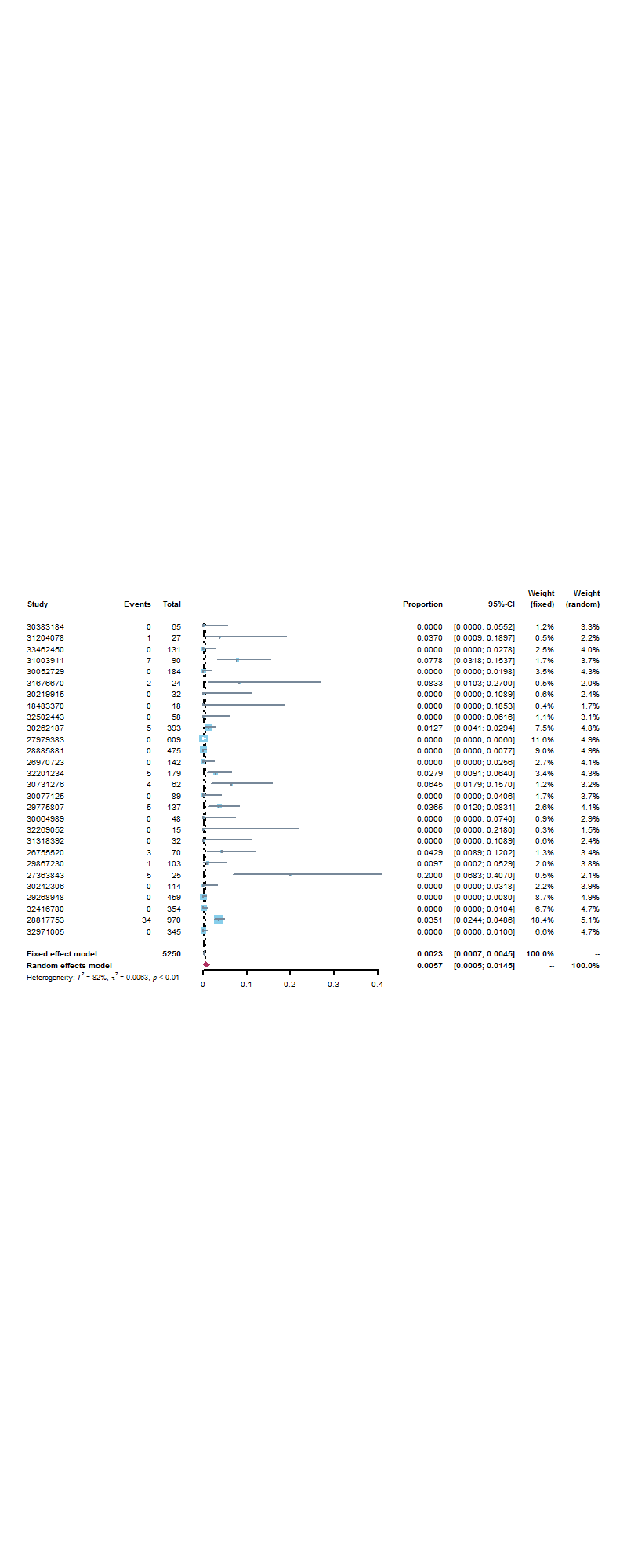


Figure 3 Forest plot of proportion of selected cutaneous adverse events with anti-CTLA-4 therapy


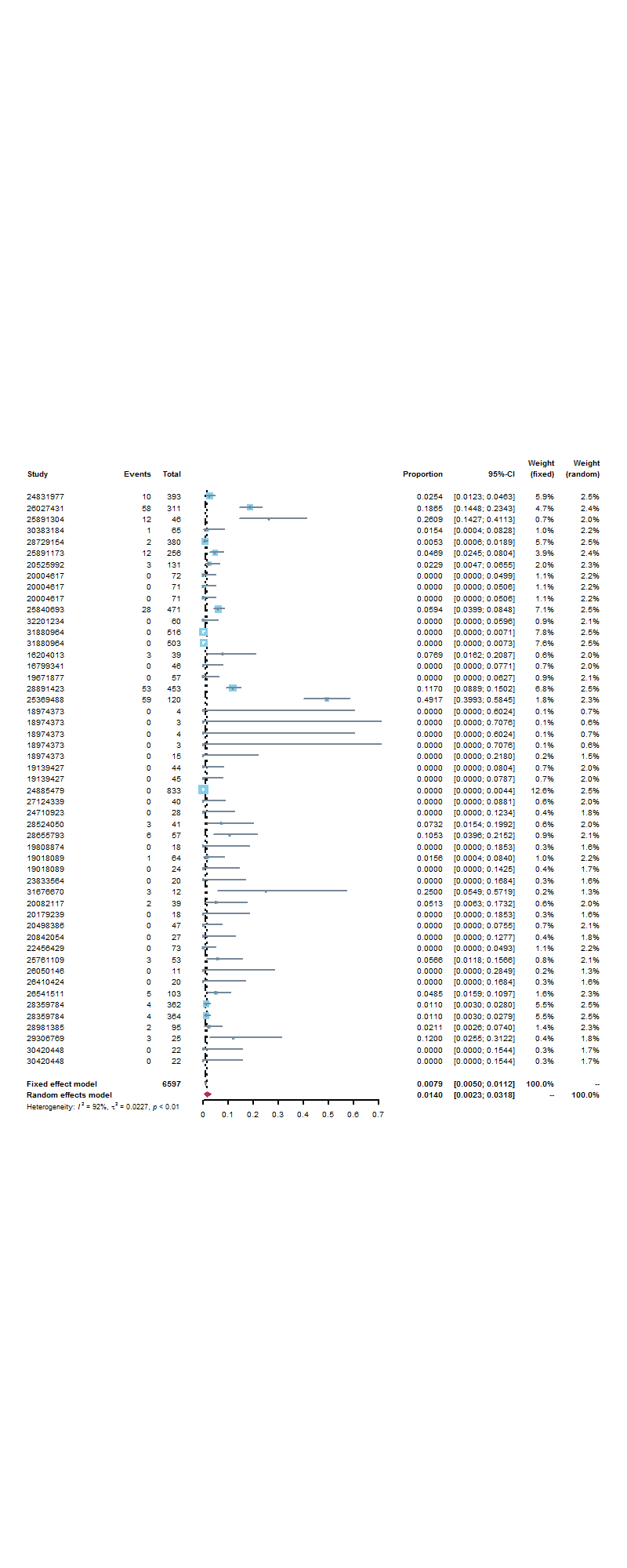


Figure 4 Forest plot of proportion of selected cutaneous adverse events with anti-PD-1/L1 plus CTLA-4 therapy


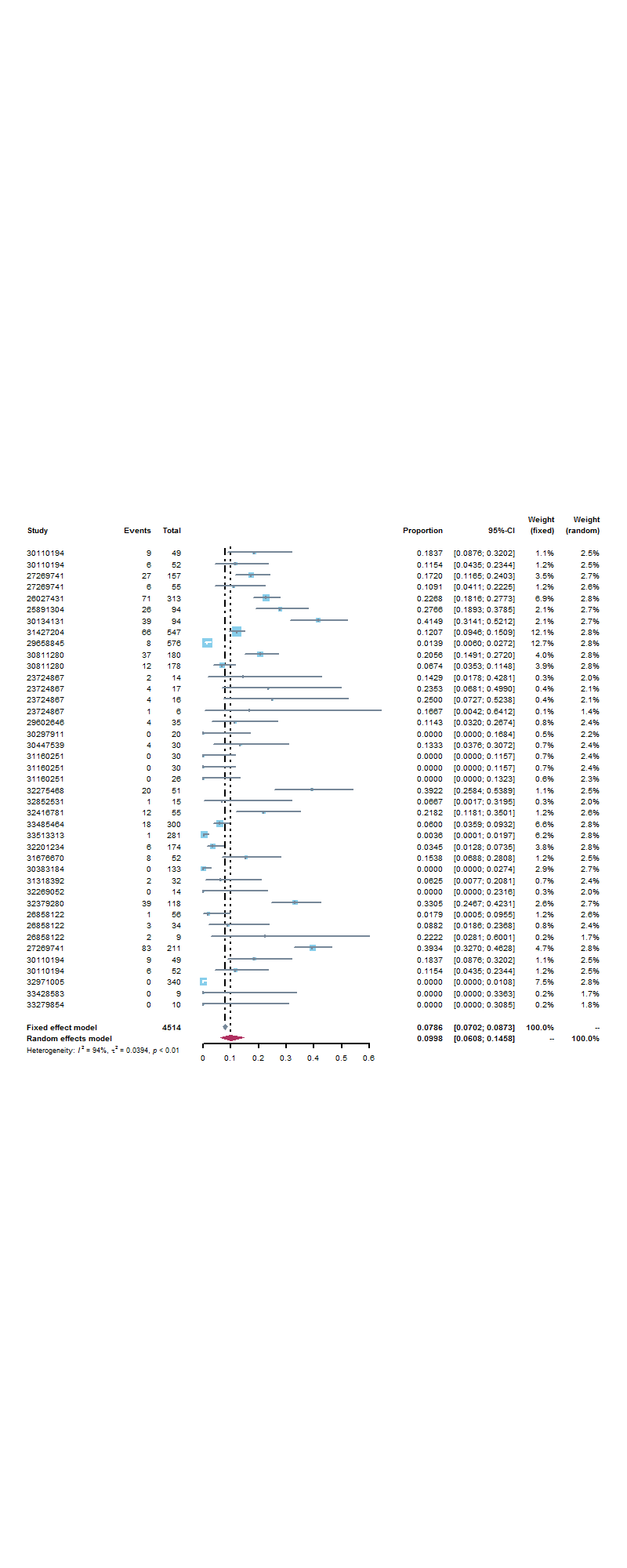


Figure 5 Forest plot of proportion of selected cutaneous adverse events with anti-PD-1/L1 plus Chemotherapy


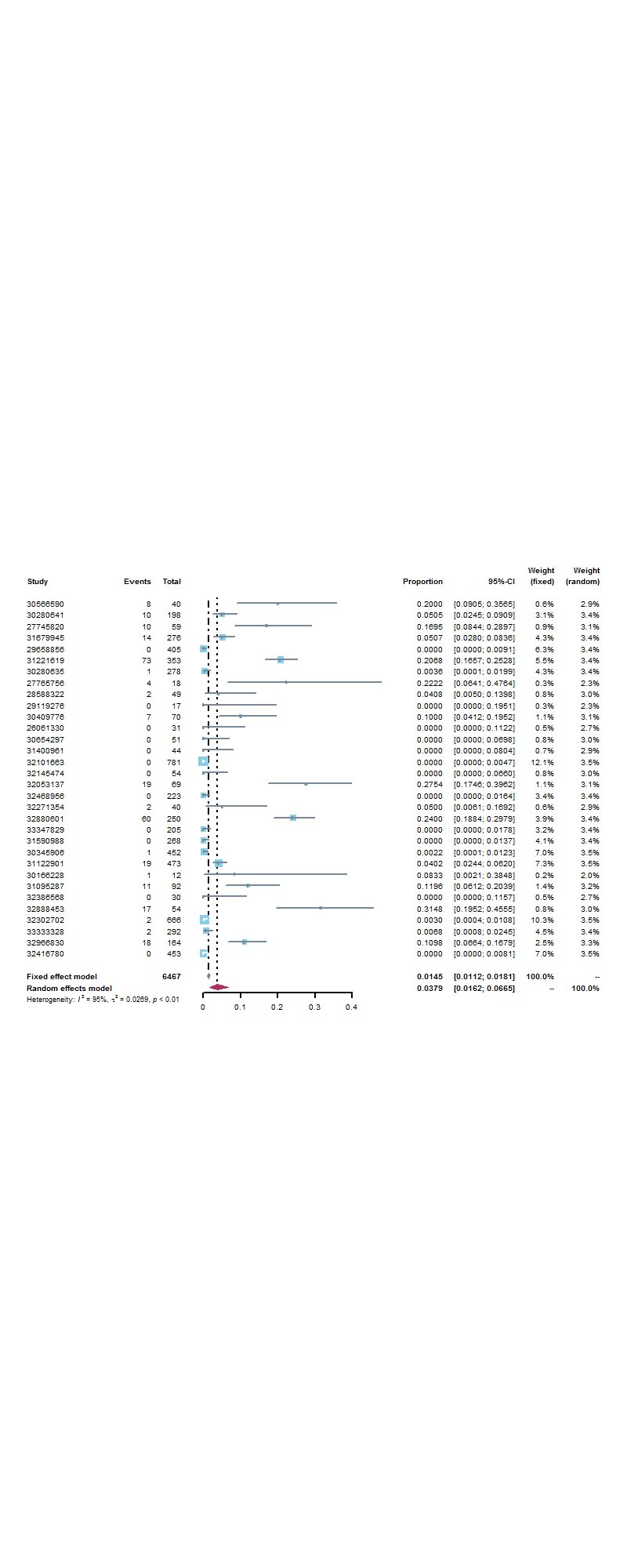


Figure 6 Forest plot of proportion of selected cutaneous adverse events with anti-CTLA-4 plus Chemotherapy


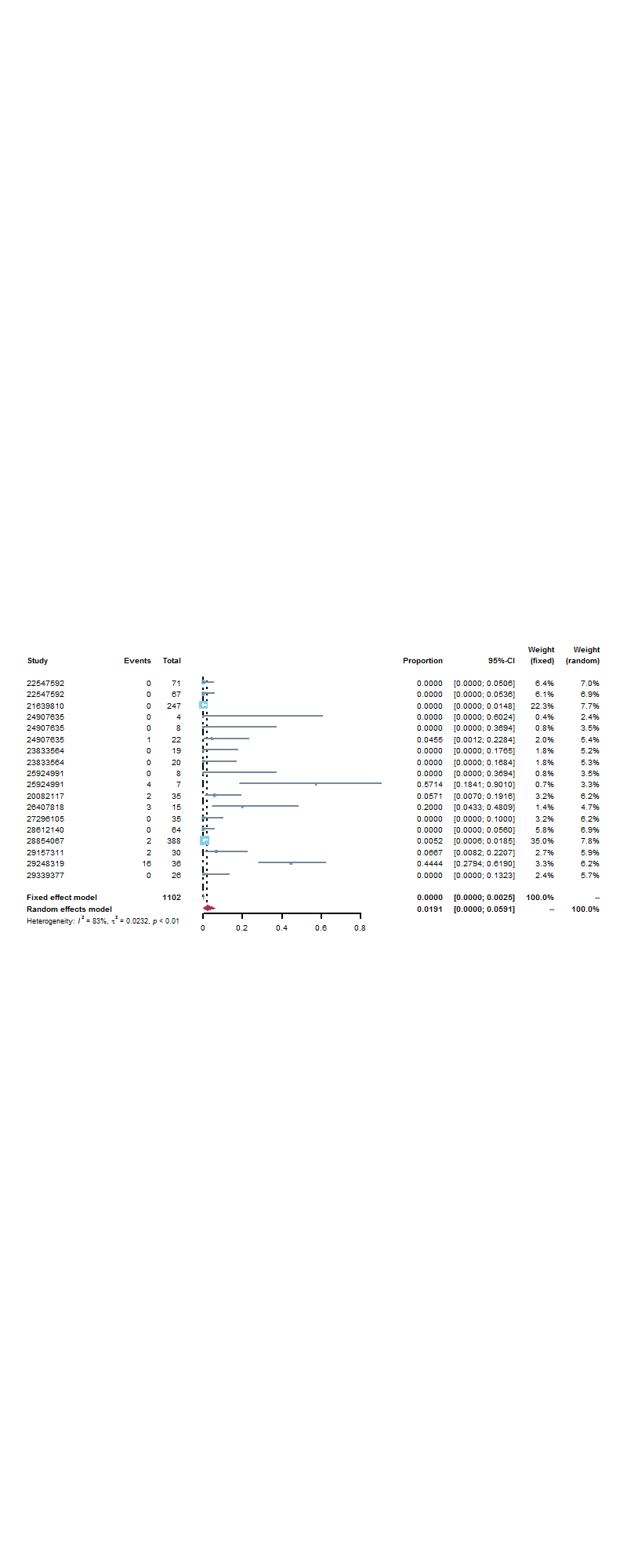


Figure 7 Forest plot of proportion of selected cutaneous adverse events with anti-PD-1/L1 plus EGF targeted therapy


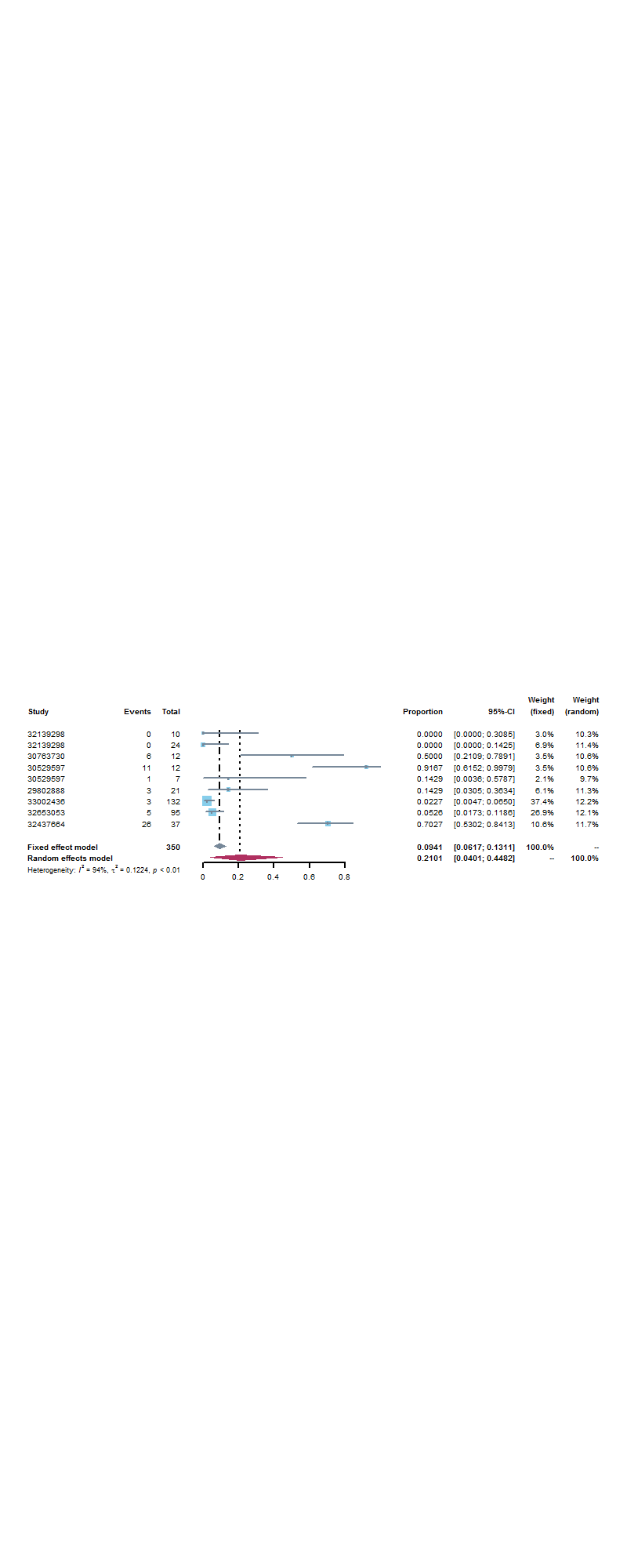


Figure 8 Forest plot of proportion of selected cutaneous adverse events with anti-PD-1/L1 plus EGFR tyrosine kinase inhibitor


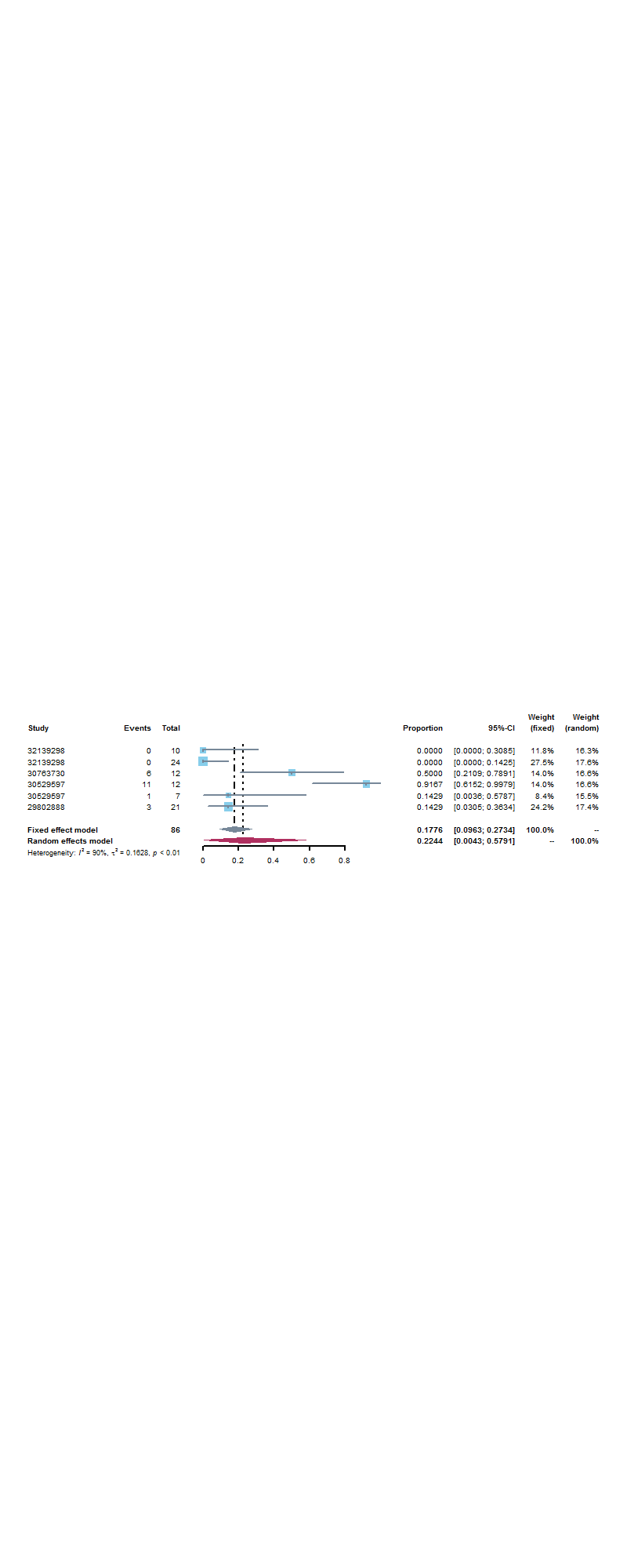


Figure 9 Forest plot of proportion of selected cutaneous adverse events with anti-PD-1/L1 plus EGF monoclonal antibodies


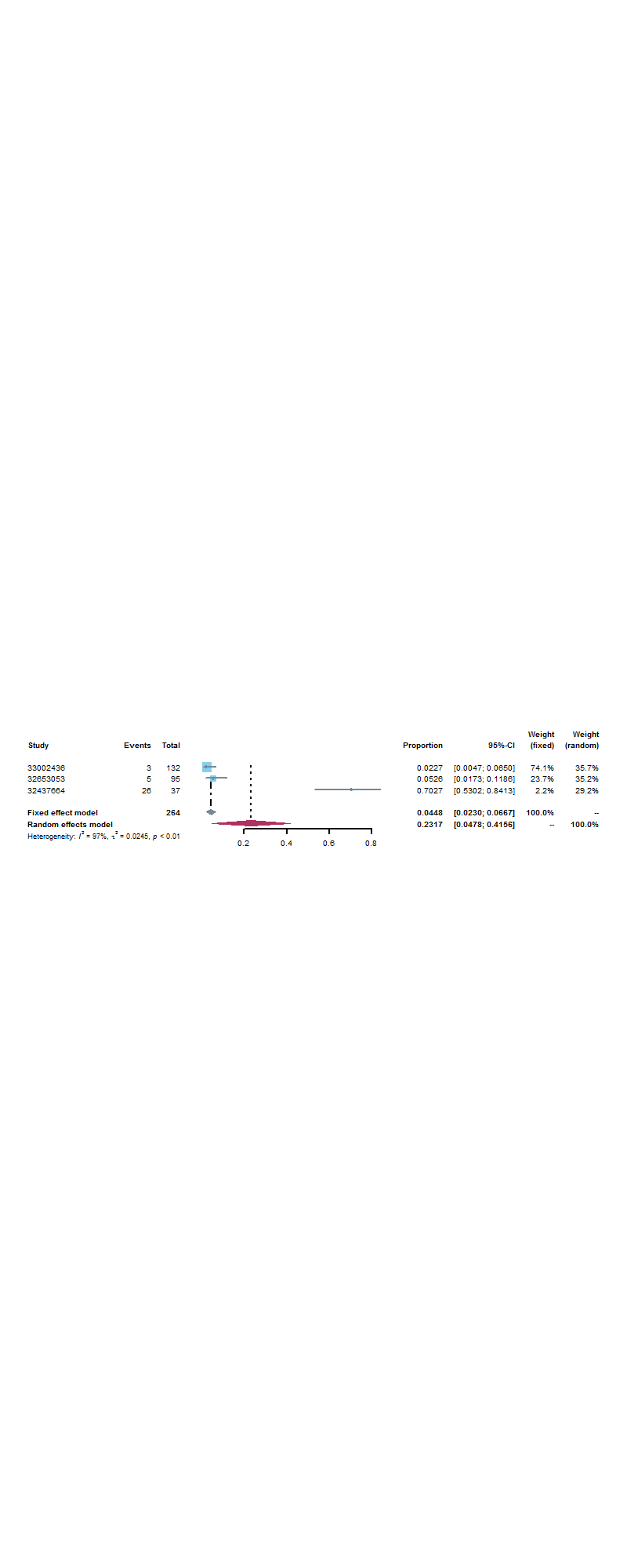


Figure 10 Forest plot of proportion of selected cutaneous adverse events with anti-PD-1/L1 plus VEGF targeted therapy


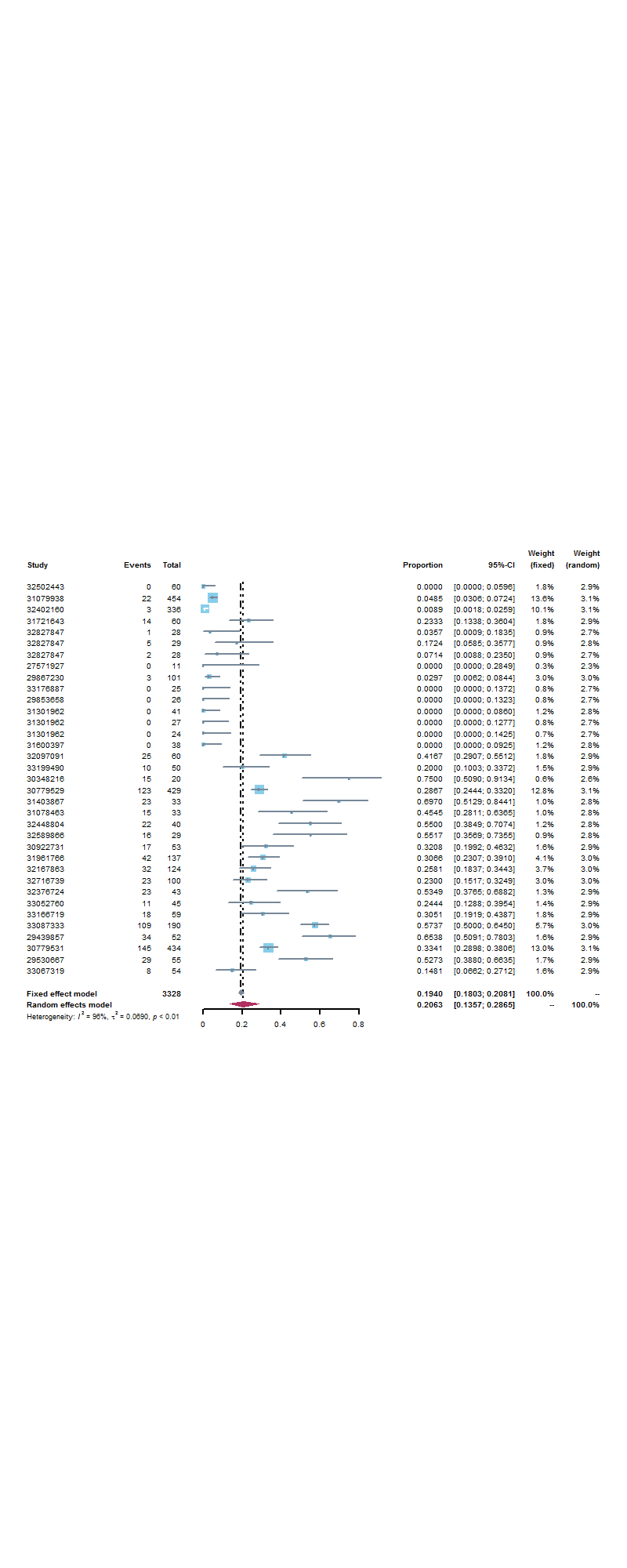


Figure 11 Forest plot of proportion of selected cutaneous adverse events with anti-PD-1/L1 plus VEGFR tyrosine kinase inhibitor


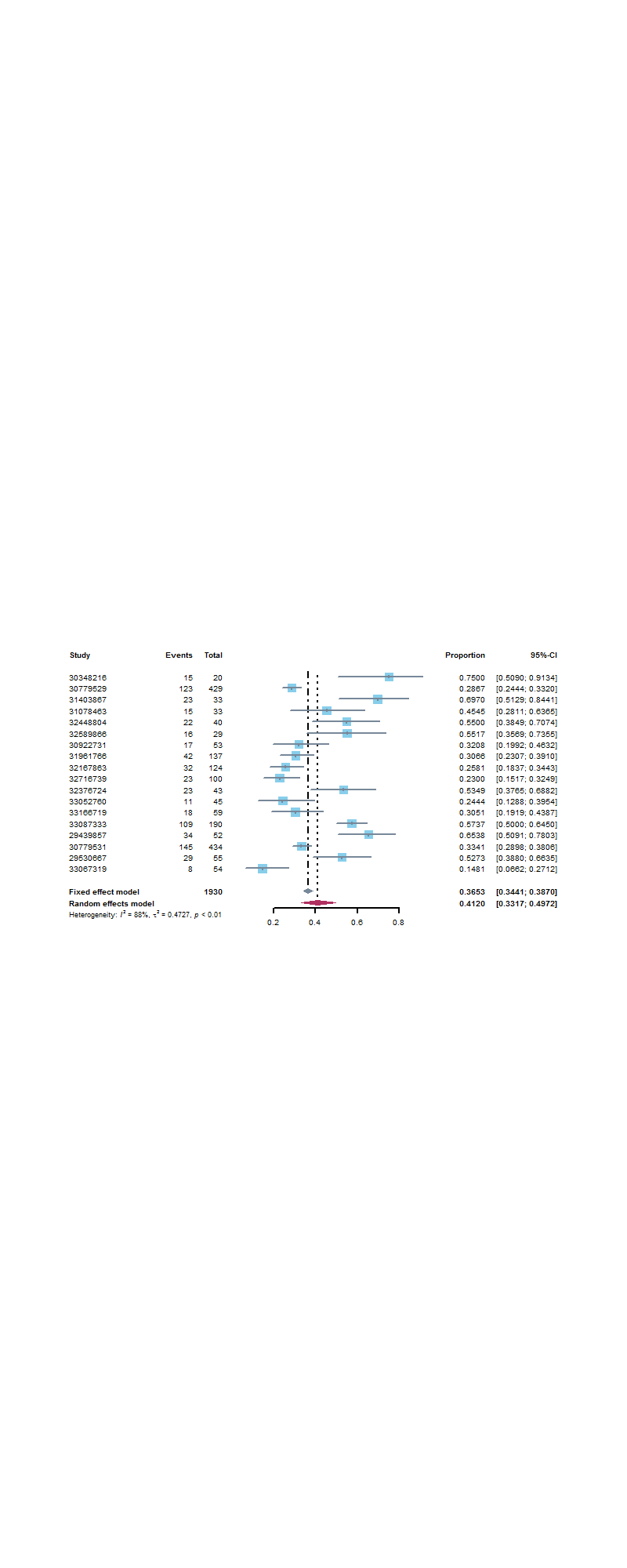


Figure 12 Forest plot of proportion of selected cutaneous adverse events with anti-PD-1/L1 plus VEGF monoclonal antibodies


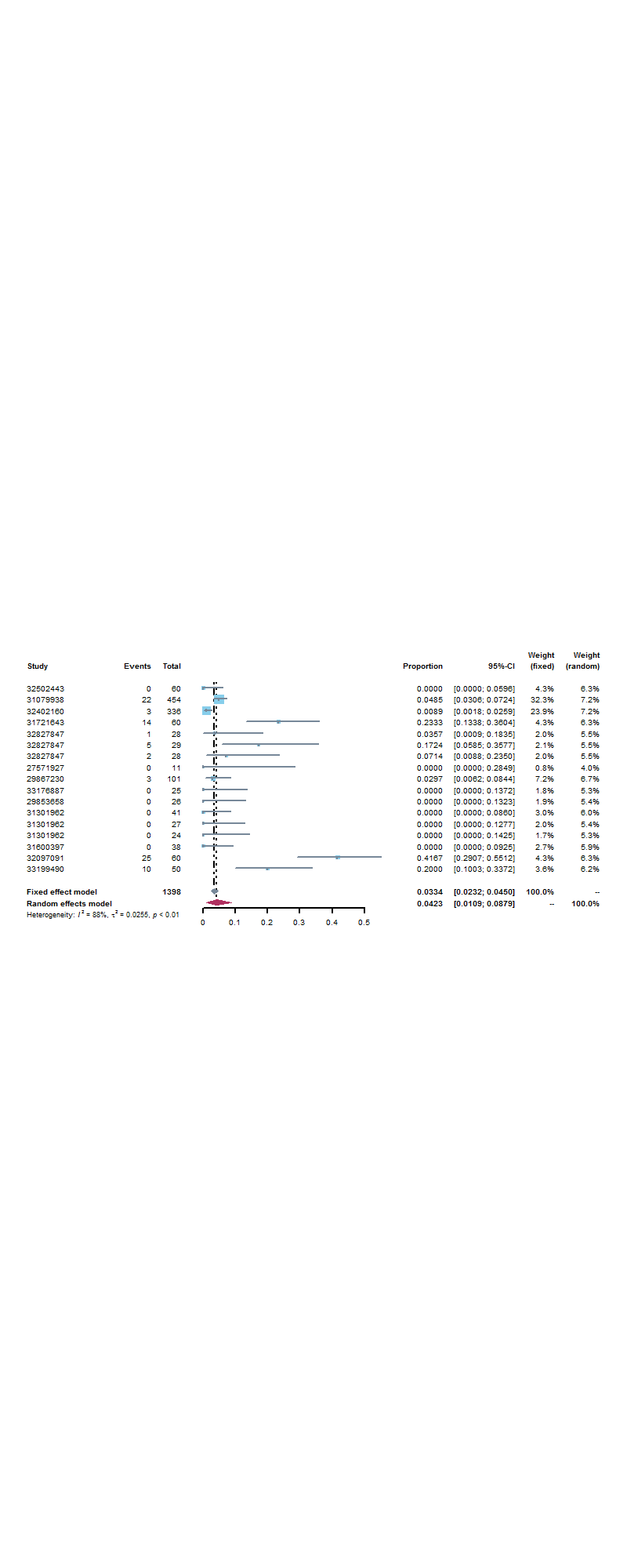

Supplement: Supplementary file 1 [file DataSheet4.docx]
