# Supplementary material for: Selected cutaneous adverse events in patients treated with ICI monotherapy and combination therapy: a retrospective pharmacovigilance study and meta-analysis
Source: Front Pharmacol. 2023 Jun 2;14:1076473. doi: 10.3389/fphar.2023.1076473 (PMC10272362; doi:10.3389/fphar.2023.1076473)
Supplement: Supplementary file 2 [file Table1.docx]

Appendix Table 1. Proportion and Types of Selected Immune Checkpoint Inhibitor-Related Dermatologic adverse events From Systematic Review and Meta-analysis.

| **Variable** | **Anti-PD-1**  **(n=17864）** | **Anti-PD-L1**  **(n=5250)** | **Anti-CTLA-4**  **(n=6597）** | **Anti-PD-1/L1+CTLA-4**  **(n=4514）** | **Anti-PD-1/L1+Chemotherapy**  **(n=6467）** | **Anti-CTLA-4+Chemotherapy**  **(n=1102）** | **Anti-PD-1/L1+EGF TKI**  **(n=86）** | **Anti-PD-1/L1+VEGF TKI**  **(n=1963）** | **Anti-PD-1/L1+EGF Monoclonal antibodies**  **(n=264）** | **Anti-PD-1/L1+VEGF Monoclonal antibodies**  **(n=1398)** |
| --- | --- | --- | --- | --- | --- | --- | --- | --- | --- | --- |
| Total | 0.0254(0.0138;0.0393) | 0.0057(0.0005;0.0145) | 0.0140（0.0023;0.0318） | 0.0998(0.0608;0.1458)) | 0.0379(0.0162;0.0665) | 0.0191(0.0000;0.0591) | 0.2244(0.0043;0.5791) | 0.4120(0.3317;0.4972) | 0.2317(0.0478;0.4156) | 0.0423(0.0109;0.0879) |
| Vitiligo | <0.0001(0.0000;0.0005) | <0.0001(0.0000;0.0000) | <0.0001(0.0000;0.0000) | 0.0046(0.0000;0.0152) | <0.0001(0.0000;0.0015) | <0.0001(0.0000;0.0000) | / | 0.0013(0.0000;0.0083) | / | / |
| PPES | <0.0001(0.0000;0.0000) | <0.0001(0.0000;0.0000) | <0.0001(0.0000;0.0000) | <0.0001(0.0000;0.0000) | 0.0020(0.0000;0.0107) | / | / | 0.3336(0.2404;0.4268) | 0.0273(0.0000;0.1562) | 0.0075(0.0013;0.0170) |
| Bullous dermatitis | <0.0001(0.0000;0.0000) | <0.0001(0.0000;0.0000) | / | / | / | / | / | / | / | <0.0001(0.0000;0.0000) |
| Drug eruption | <0.0001(0.0000;0.0000) | <0.0001(0.0000;0.0000) | <0.0001(0.0000;0.0000) | / | <0.0001(0.0000;0.0000) | <0.0001(0.0000;0.0000) | / | <0.0001(0.0000;0.0003) | / | / |
| Erythema multiform | <0.0001(0.0000;0.0000) | / | <0.0001(0.0000;0.0000) | / | <0.0001(0.0000;0.0000) | <0.0001(0.0000;0.0000) | / | 0.0001(0.0000;0.0038) | 0.0052(0.0000;0.0262) | <0.0001(0.0000;0.0013) |
| Acneiform rash | <0.0001(0.0000;0.0000) | <0.0001(0.0000;0.0000) | <0.0001(0.0000;0.0000) | / | <0.0001(0.0000;0.0000) | 0.0002(0.0000;0.0112) | / | / | <0.0001(0.0000;0.0000) | <0.0001(0.0000;0.0031) |
| Skin exfoliation | <0.0001(0.0000;0.0000) | <0.0001(0.0000;0.0000) | / | <0.0001(0.0000;0.0000) | <0.0001(0.0000;0.0000) | / | 0.0335(0.0000;0.1112) | 0.0007(0.0000;0.0063) | / | / |
| Maculopapular rash | 0.0099(0.0036;0.0182) | <0.0001(0.0000;0.0000) | 0.0015(0.0000;0.0112) | 0.055(0.030;0.087) | 0.0097(0.0014;0.0225) | <0.0001(0.0000;0.0062) | 0.0438(0.0000;0.1401) | 0.0185(0.0019;0.0459) | 0.1040(0.0000;0.4196) | 0.0164(0.0003;0.0471) |
| Skin ulceration | <0.0001(0.0000;0.0000) | / | <0.0001(0.0000;0.0000) | / | / | <0.0001(0.0000;0.0000) | / | 0.0002(0.0000;0.0038) | / | <0.0001(0.0000;0.0000) |
| Urticaria | <0.0001(0.0000;0.0000) | / | <0.0001(0.0000;0.0000) | <0.0001(0.0000;0.0000) | <0.0001(0.0000;0.0000) | <0.0001(0.0000;0.0000) | 0.0123(0.0000;0.0688) | <0.0001(0.0000;0.0017) | / | <0.0001(0.0000;0.0000) |
| Stevens-Johnson syndrome | <0.0001(0.0000;0.0000) | <0.0001(0.0000;0.0000) | <0.0001(0.0000;0.0000) | <0.0001(0.0000;0.0000) | / | / | / | / | / | / |
| Toxic epidermal necrolysis | / | <0.0001(0.0000;0.0000) | / | / | <0.0001(0.0000;0.0000) | / | / | / | / | <0.0001(0.0000;0.0000) |
