# Supplementary material for: Selected cutaneous adverse events in patients treated with ICI monotherapy and combination therapy: a retrospective pharmacovigilance study and meta-analysis
Source: Front Pharmacol. 2023 Jun 2;14:1076473. doi: 10.3389/fphar.2023.1076473 (PMC10272362; doi:10.3389/fphar.2023.1076473)

Forest plot of proportion of PPES with anti-PD-1 therapy


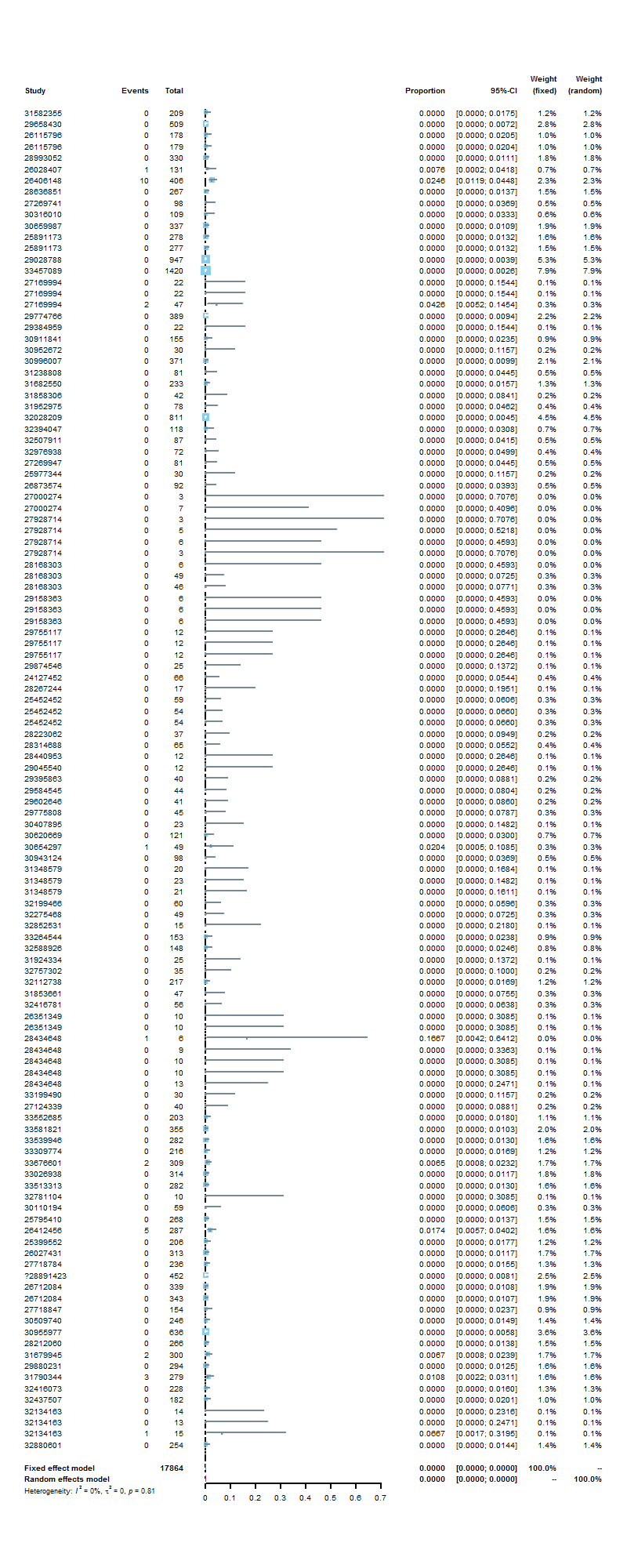


Forest plot of proportion of PPES with anti-PD-L1 therapy


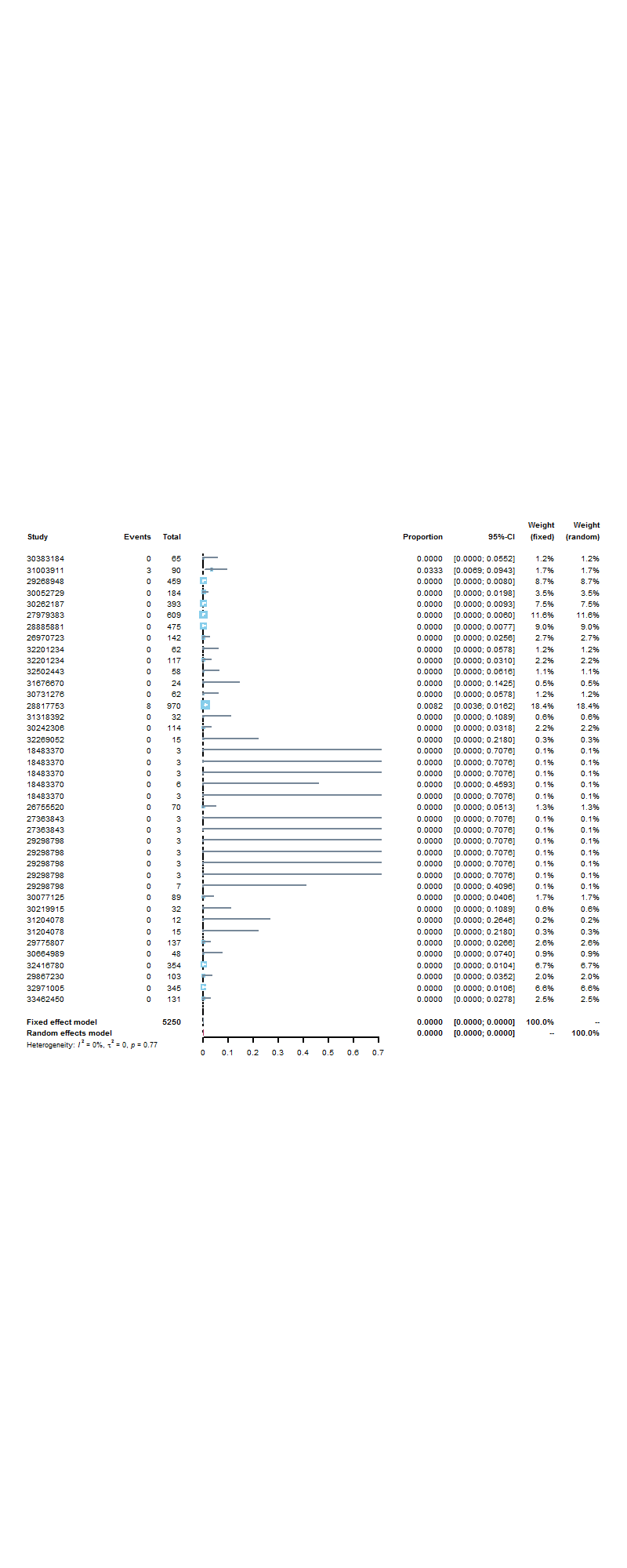


Forest plot of proportion of PPES with anti-CTLA-4 therapy


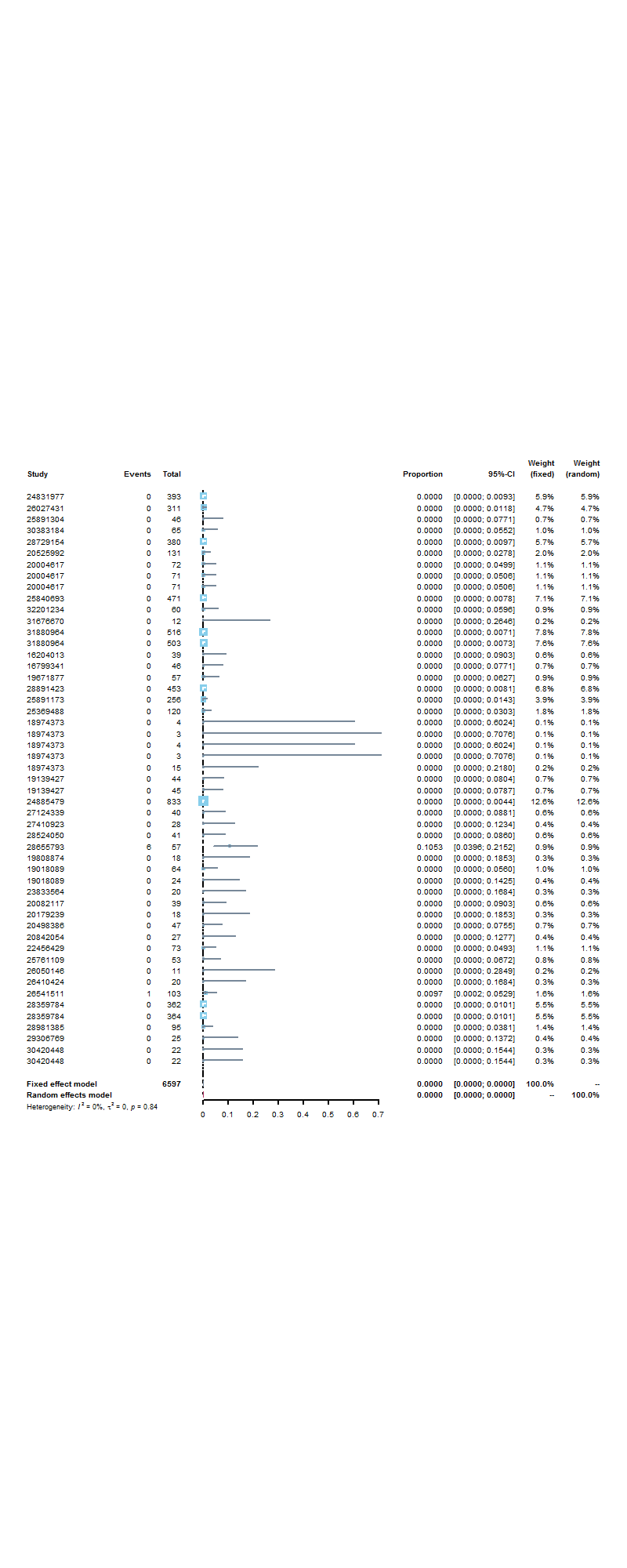


Forest plot of proportion of PPES with anti-PD-1/L1 plus chemotherapy


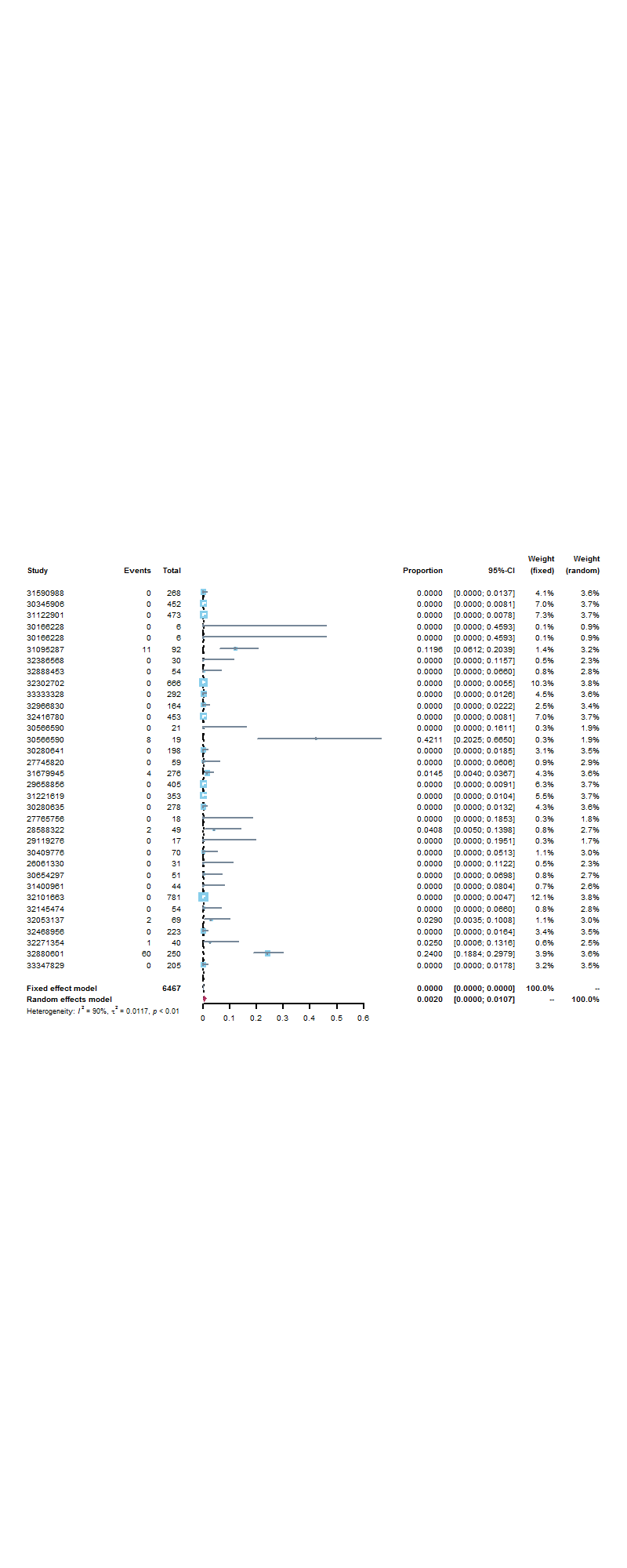


Forest plot of proportion of PPES with anti-PD-1/L1 plus EGFR MA


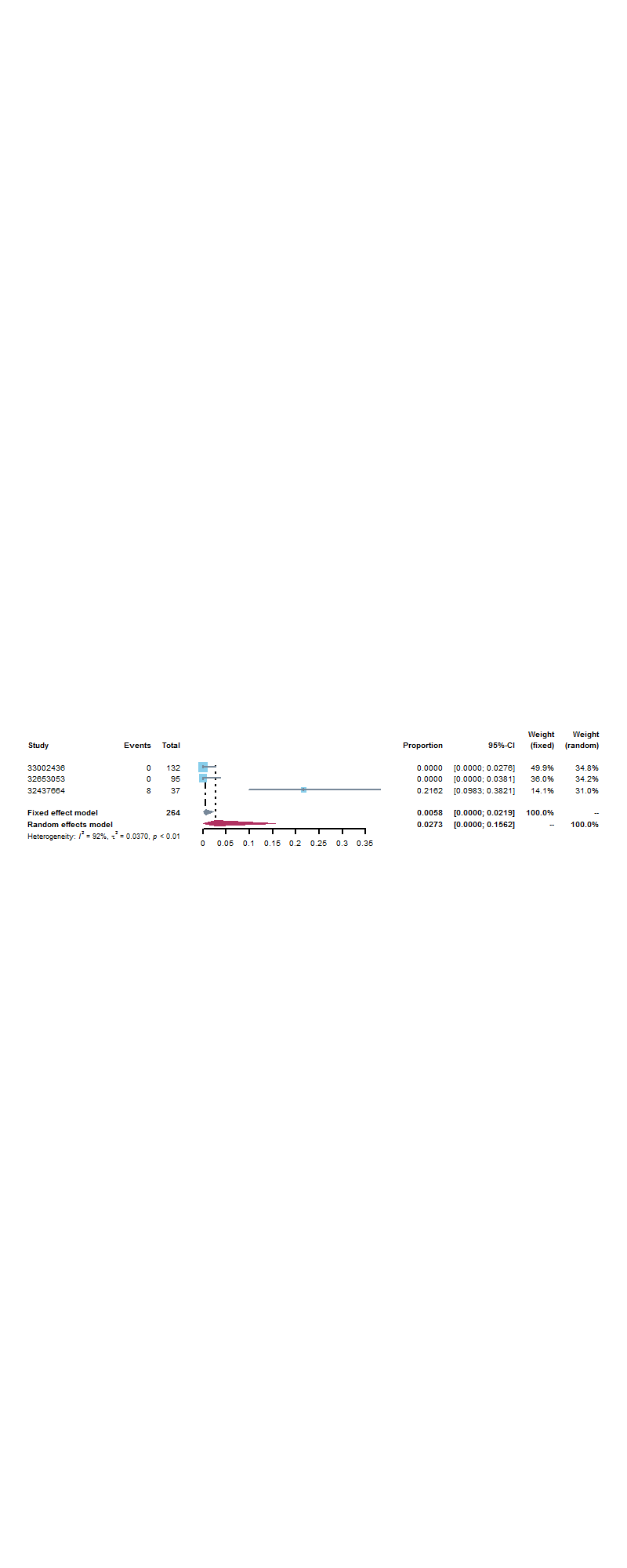


Forest plot of proportion of PPES with anti-PD-1/L1 plus VEGFR TKI


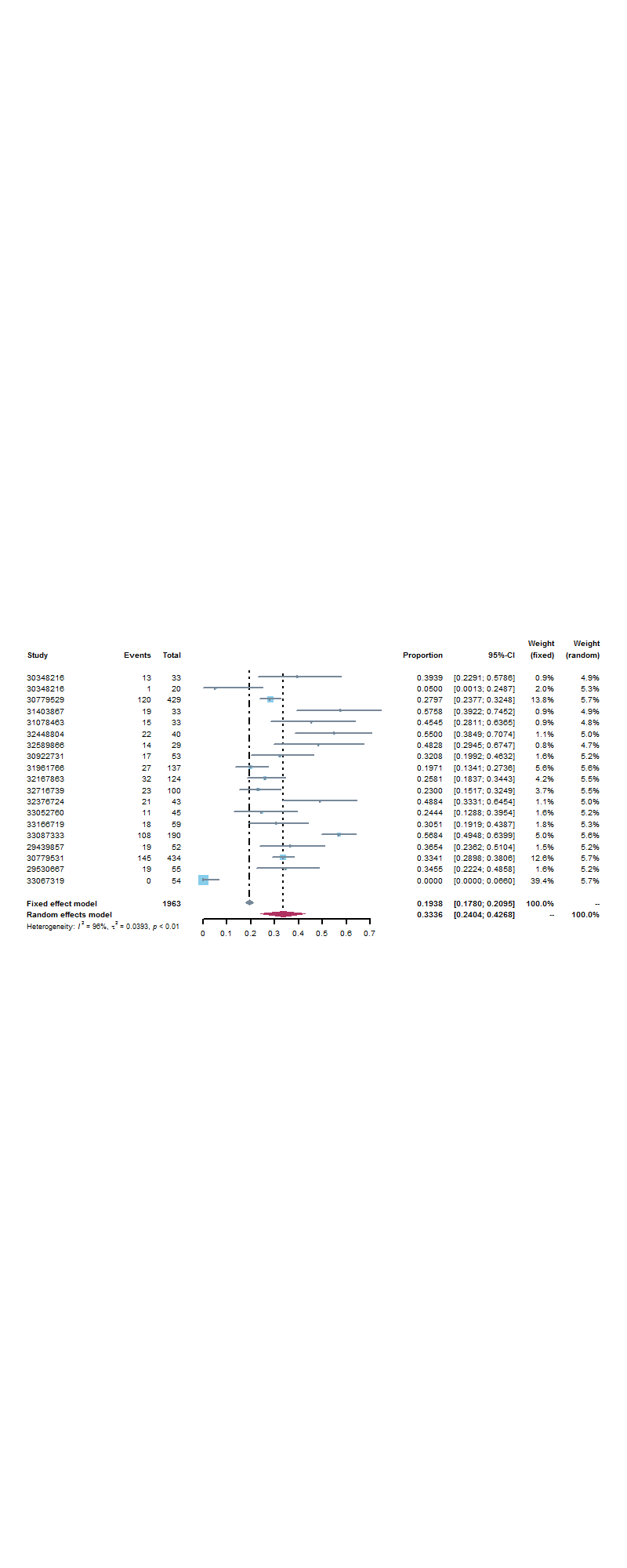


Forest plot of proportion of PPES with anti-PD-1/L1 plus VEGFR MA


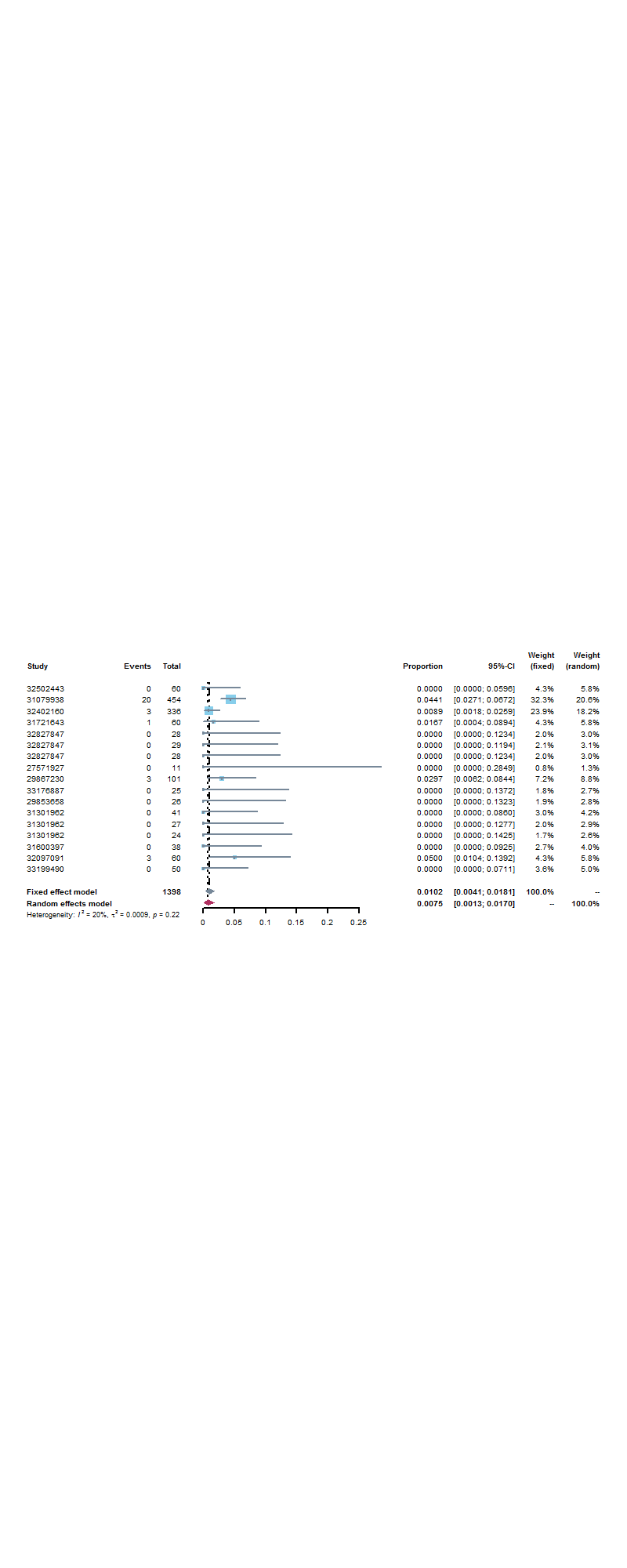


Forest plot of proportion of Vitiligo with anti-PD-1 therapy


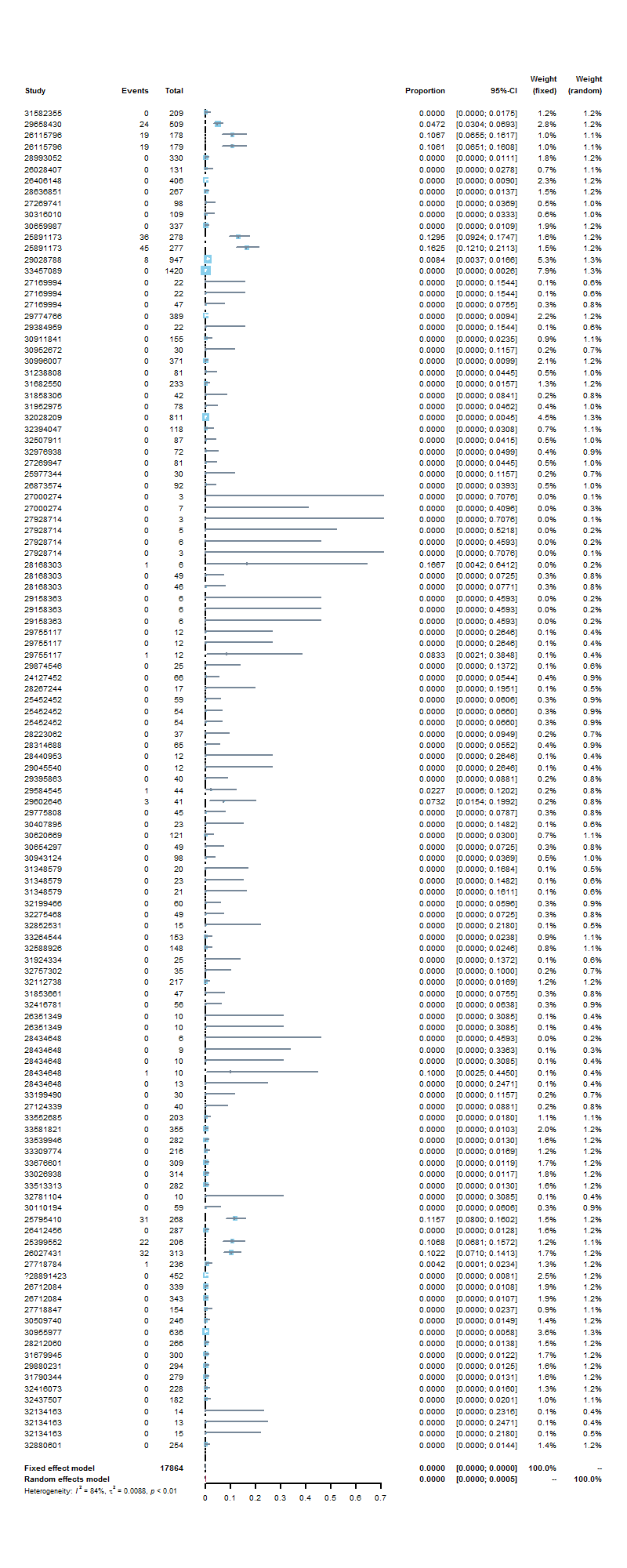


Forest plot of proportion of Vitiligo with anti-PD-L1 therapy


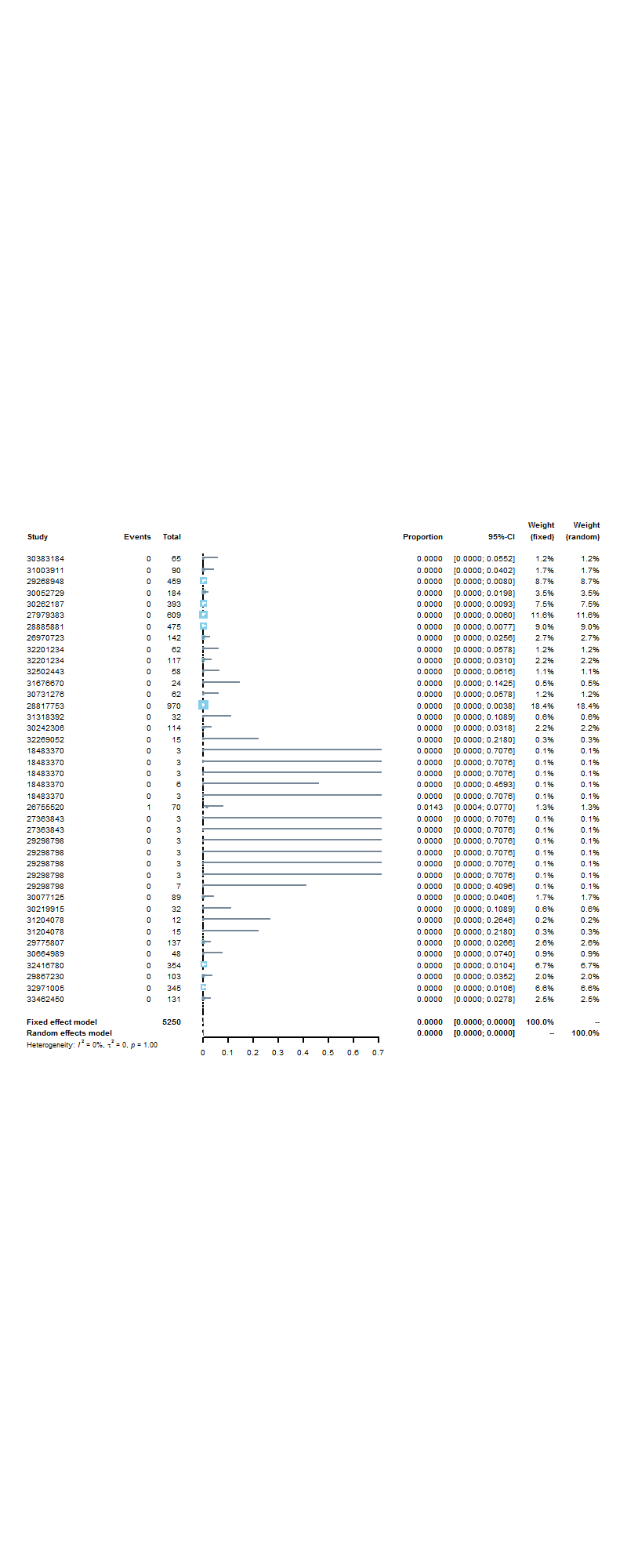


Forest plot of proportion of Vitiligo with anti-CTLA-4 therapy


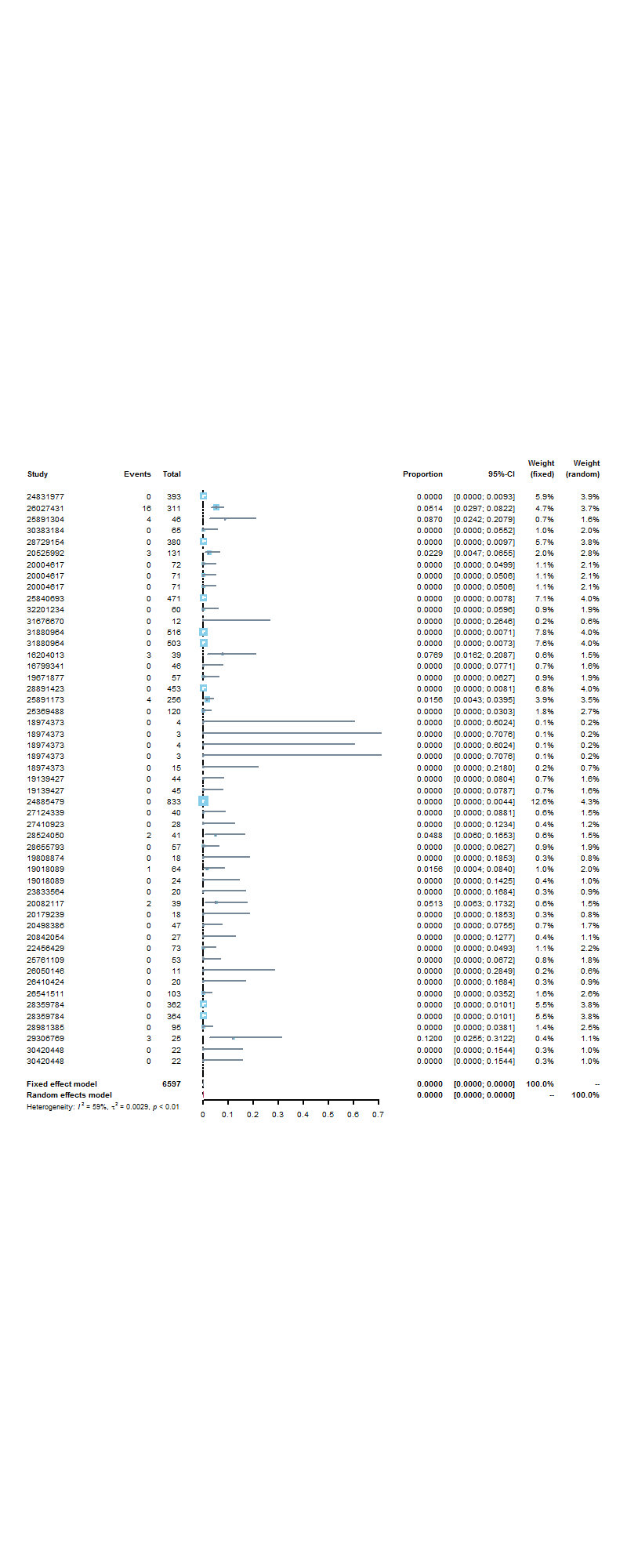


Forest plot of proportion of Vitiligo with anti-PD-1/L1 plus anti-CTLA-4 therapy


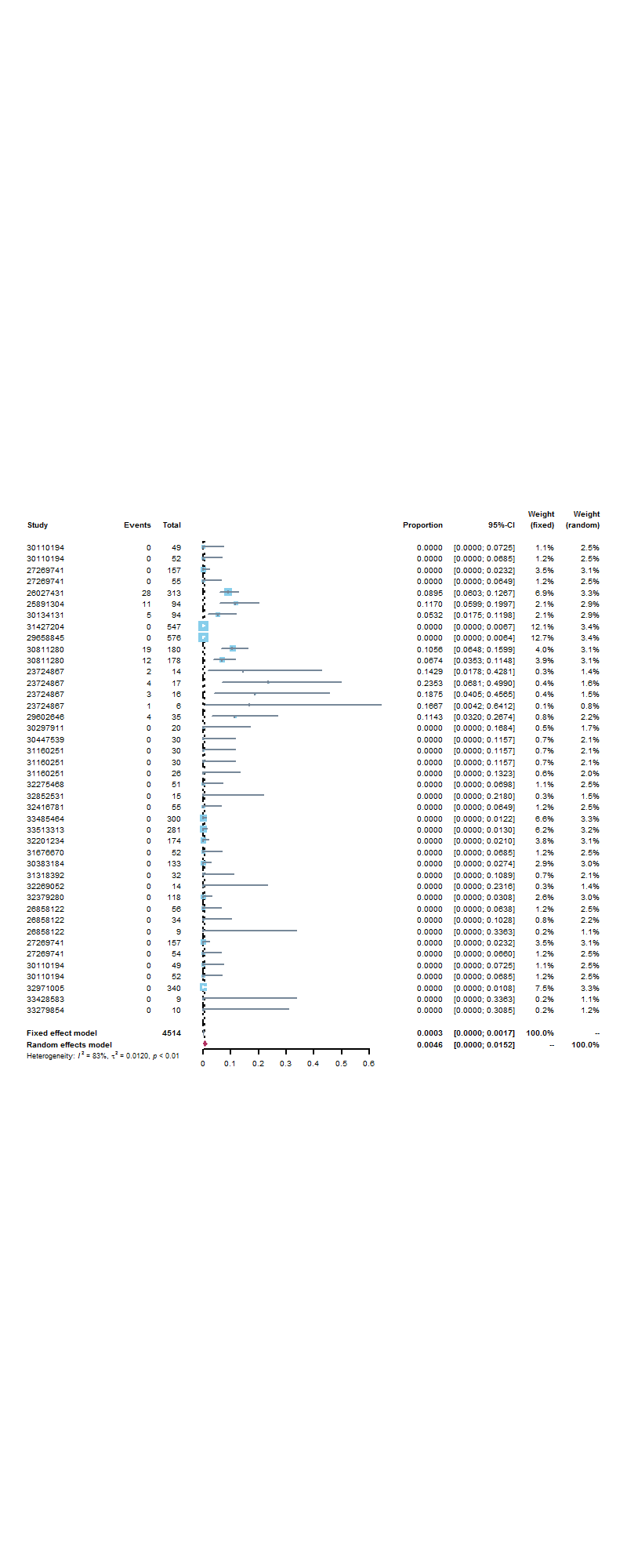


Forest plot of proportion of Vitiligo with anti-PD-1/L1 plus chemotherapy


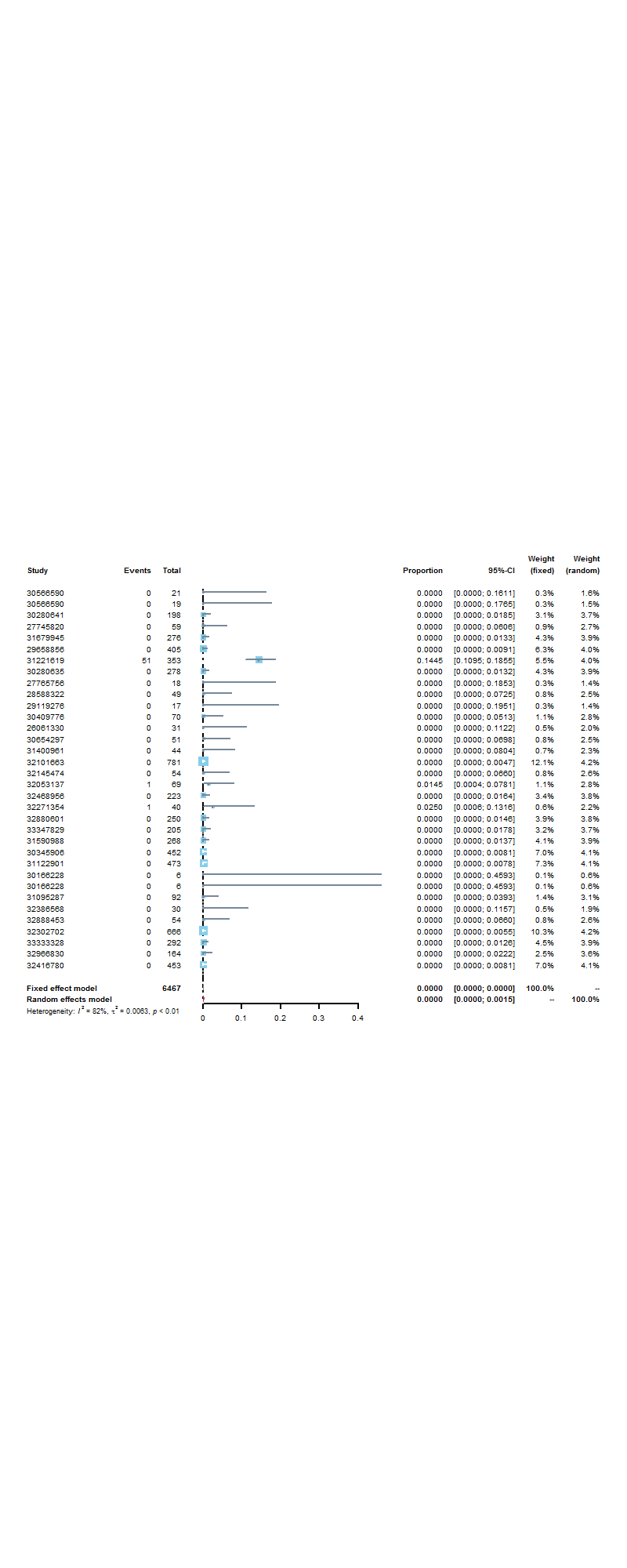


Forest plot of proportion of Vitiligo with anti-CTLA-4 plus chemotherapy


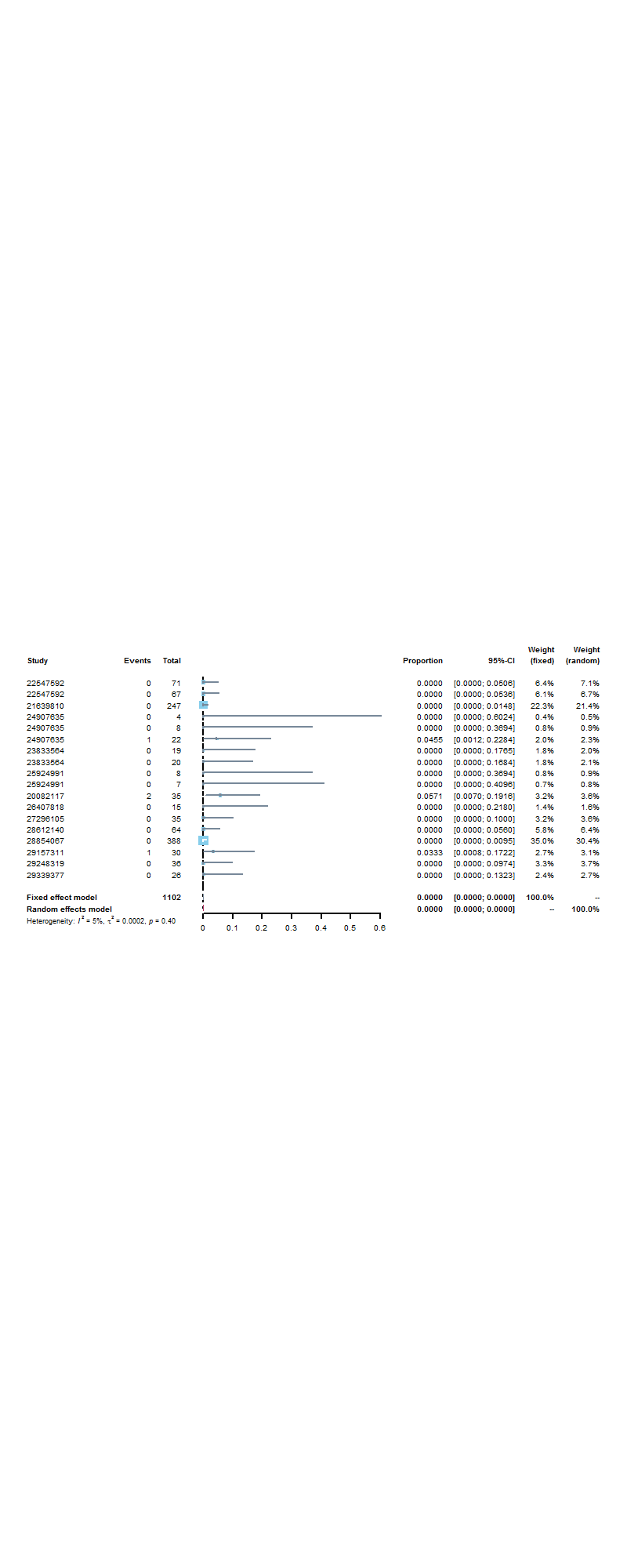


Forest plot of proportion of Vitiligo with anti-PD-1/L1 plus VEGFR TKI


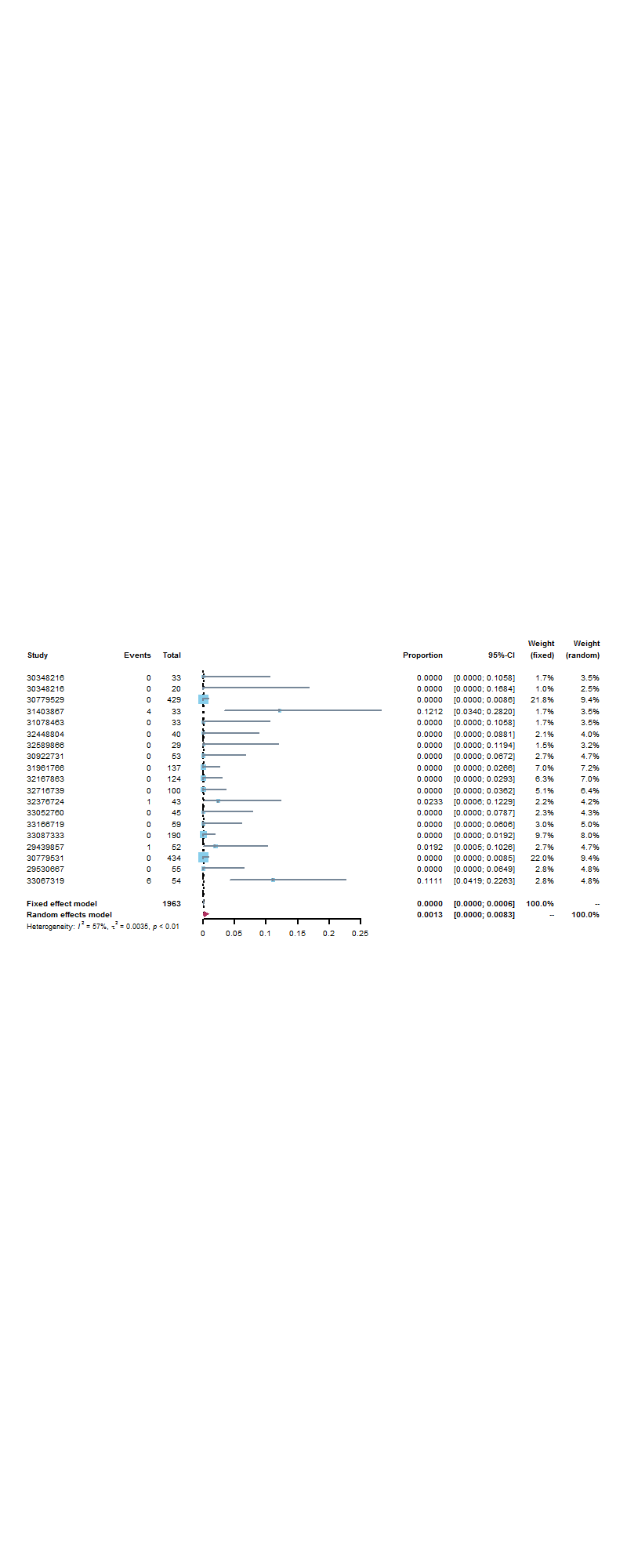


Forest plot of proportion of Maculopapular rash with anti-PD-1 therapy


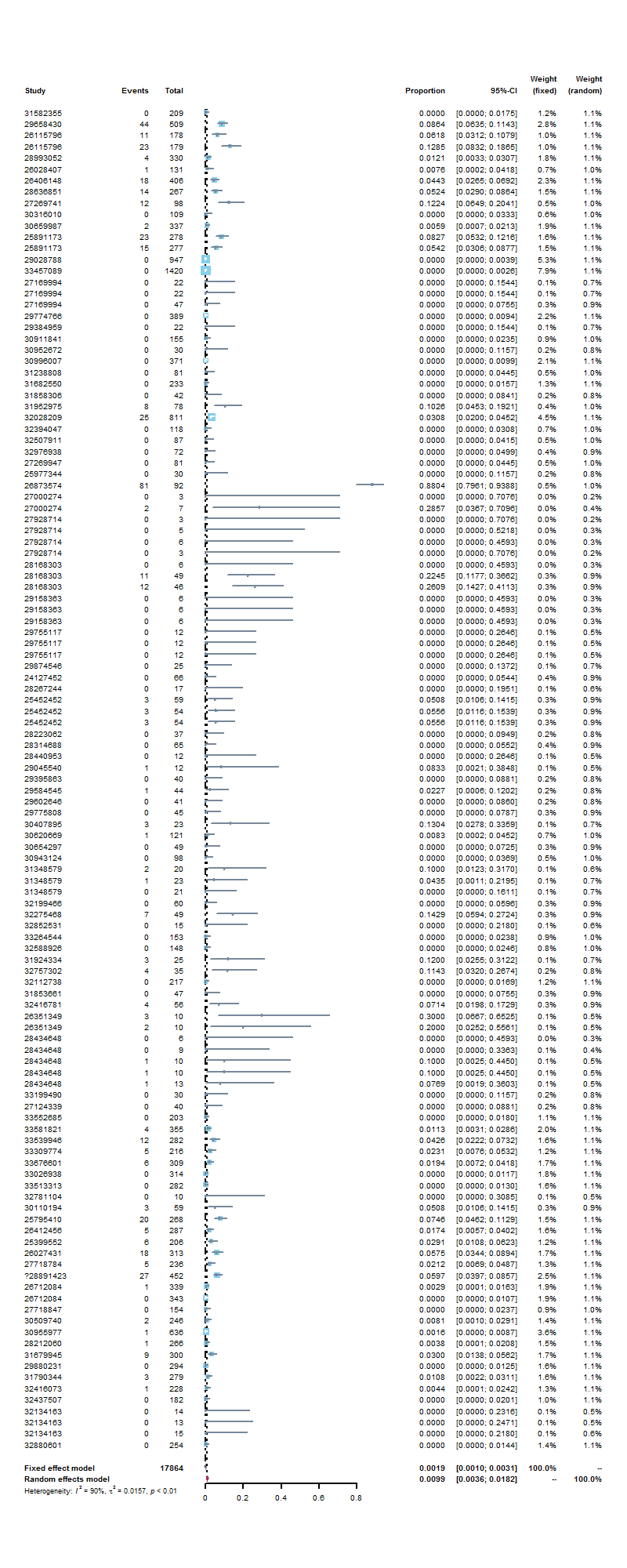


Forest plot of proportion of Maculopapular rash with anti-PD-L1 therapy


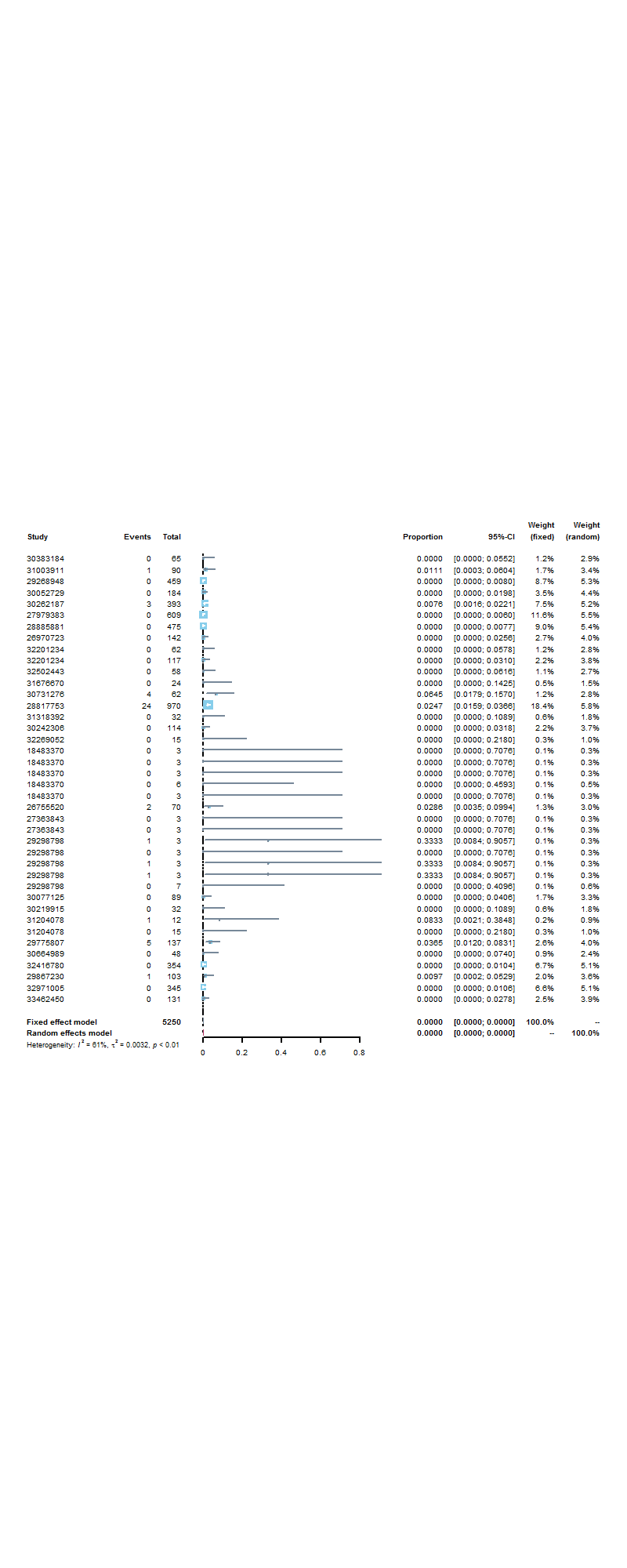


Forest plot of proportion of Maculopapular rash with anti-CTLA-4 therapy


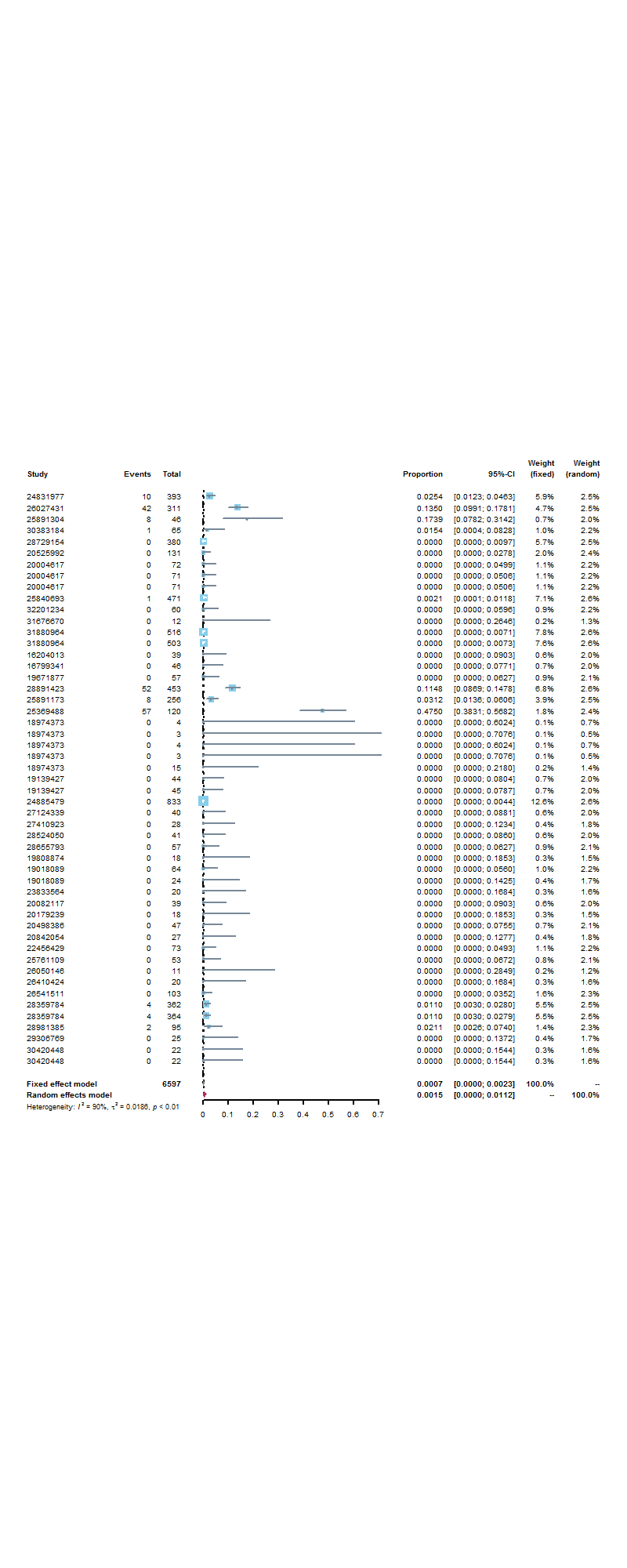


Forest plot of proportion of Maculopapular rash with anti-PD-1/L1 plus anti-CTLA-4 therapy


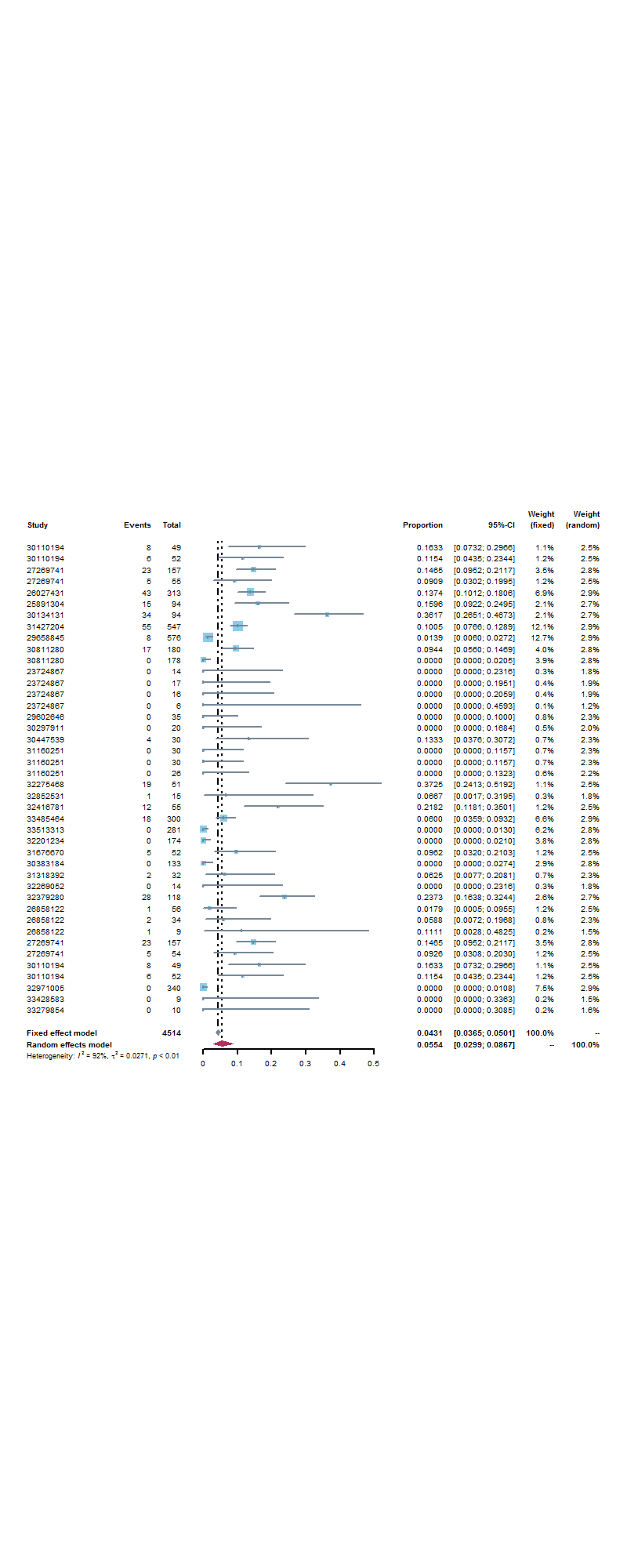


Forest plot of proportion of Maculopapular rash with anti-PD-1/L1 plus chemotherapy


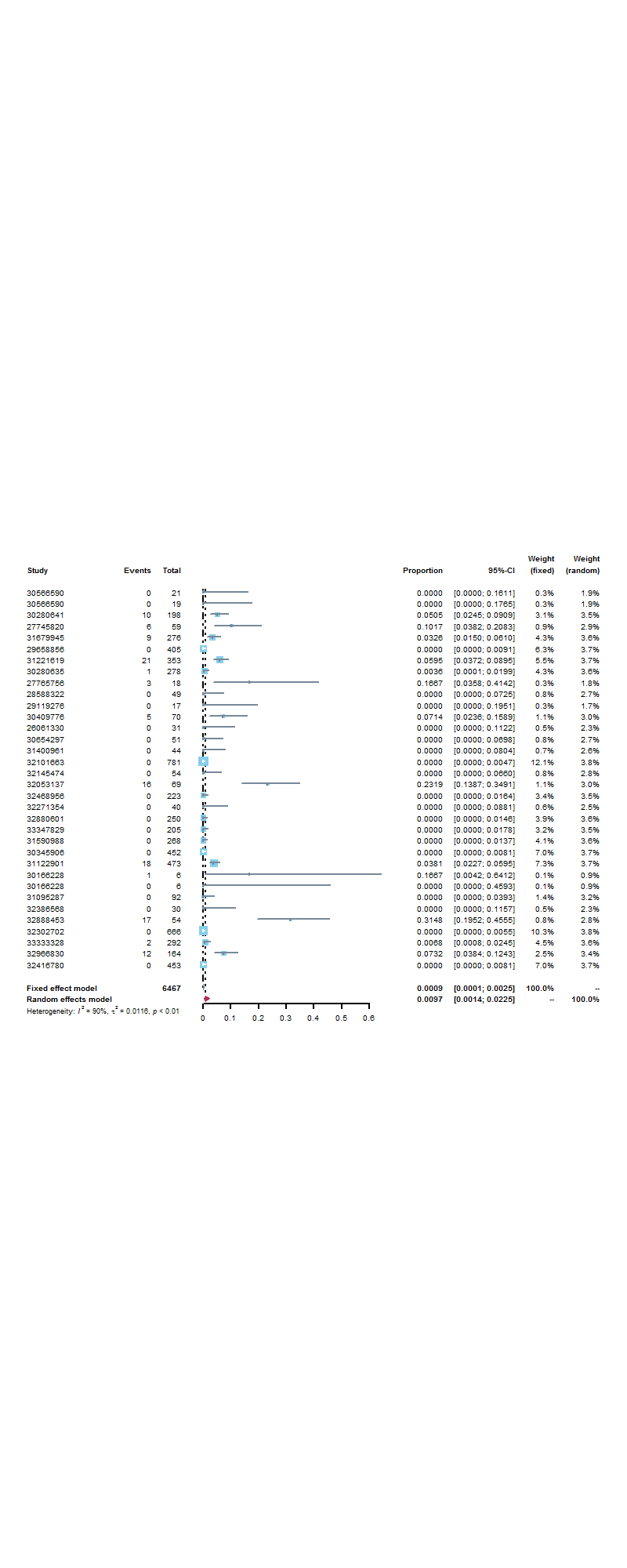


Forest plot of proportion of Maculopapular rash with anti-CTLA-4 plus chemotherapy


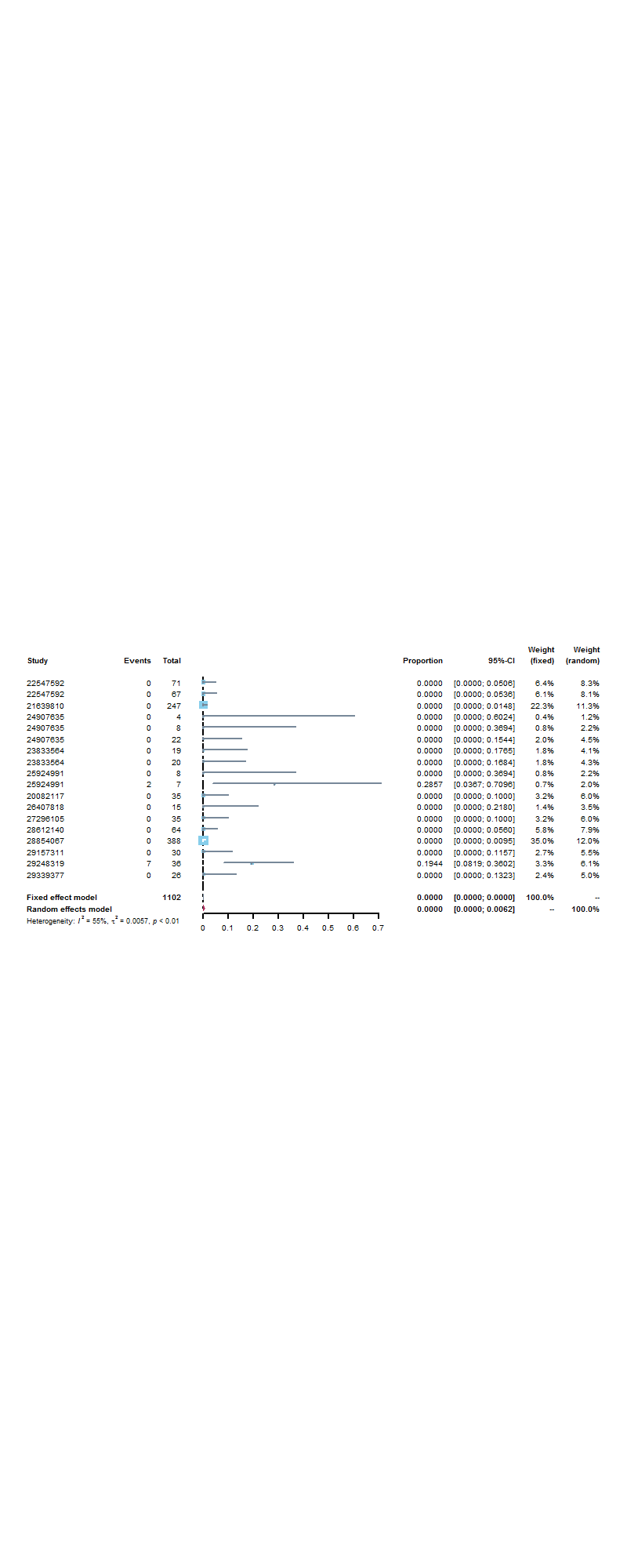


Forest plot of proportion of Maculopapular rash with anti-PD-1/L1 plus EGFR monoclonal antibody


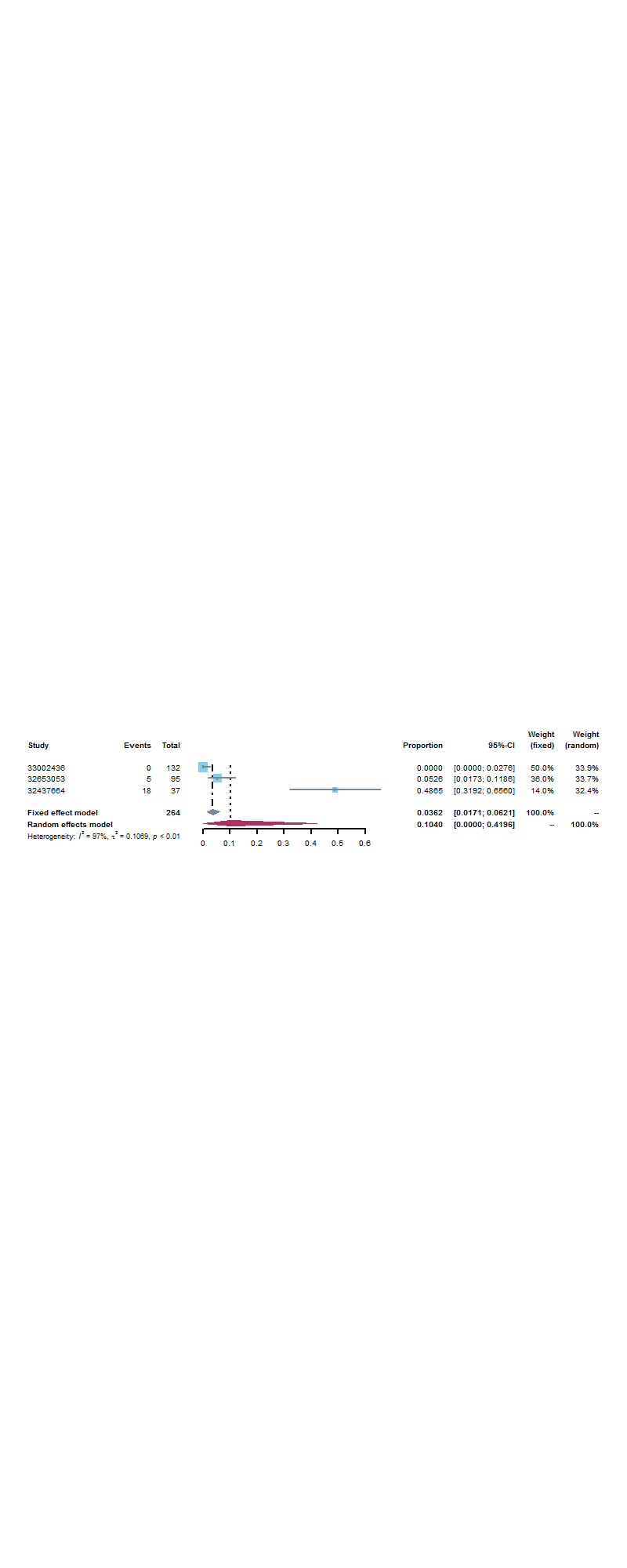


Forest plot of proportion of Maculopapular rash with anti-PD-1/L1 plus EGFR TKI


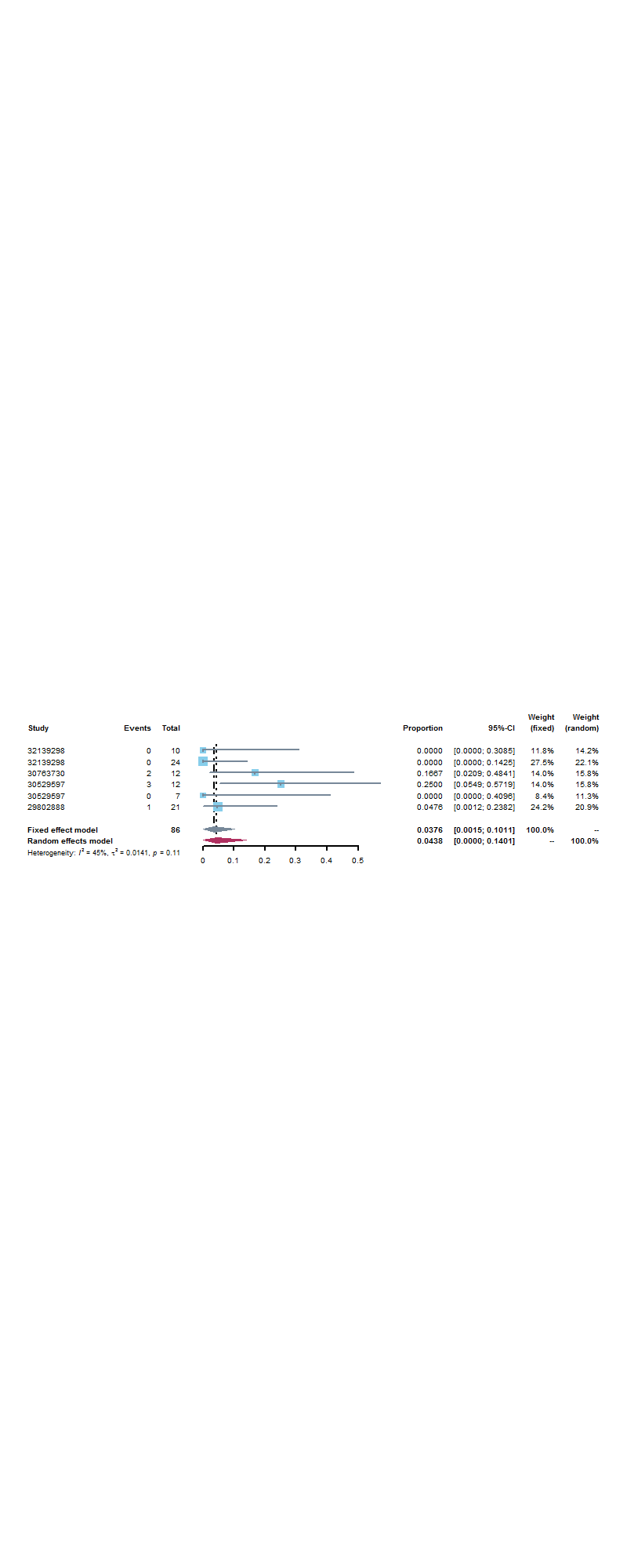


Forest plot of proportion of Maculopapular rash with anti-PD-1/L1 plus VEGFR monoclonal antibody


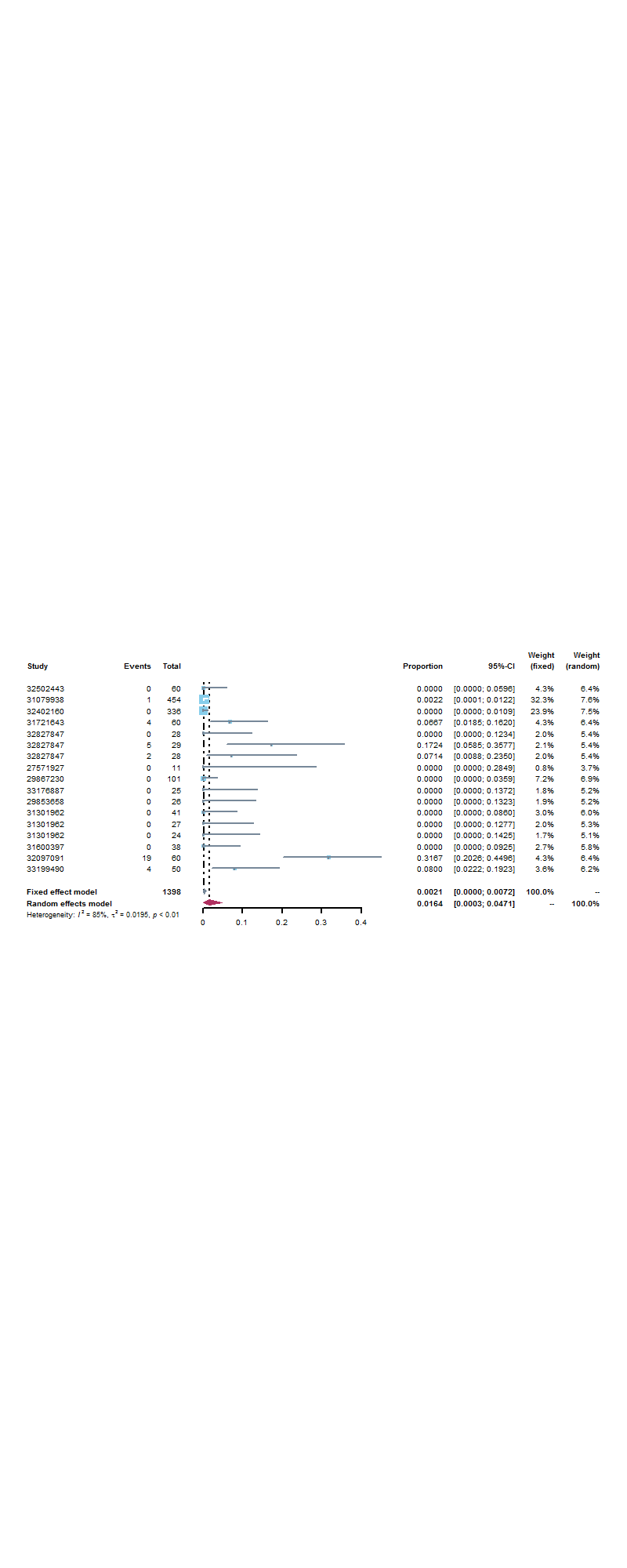


Forest plot of proportion of Maculopapular rash with anti-PD-1/L1 plus VEGFR TKI


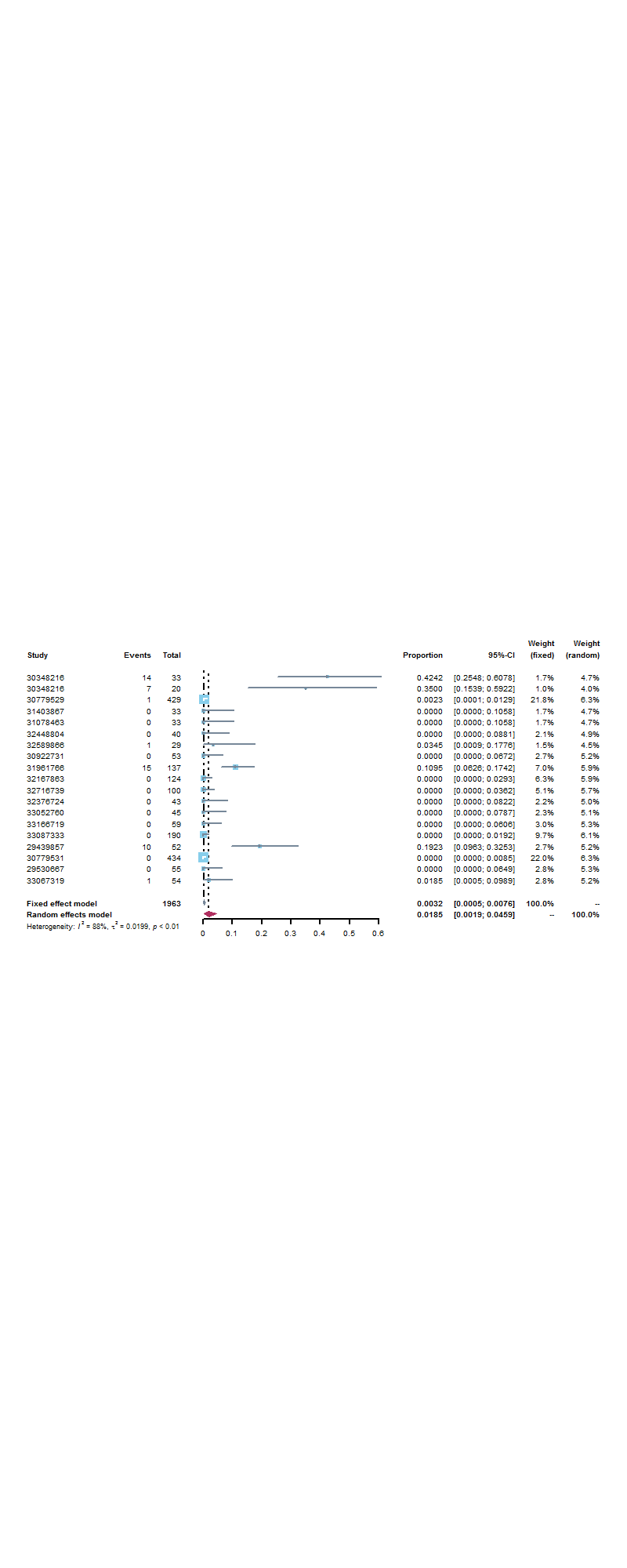


Forest plot of proportion of Erythema multiform with anti-PD-1 therapy


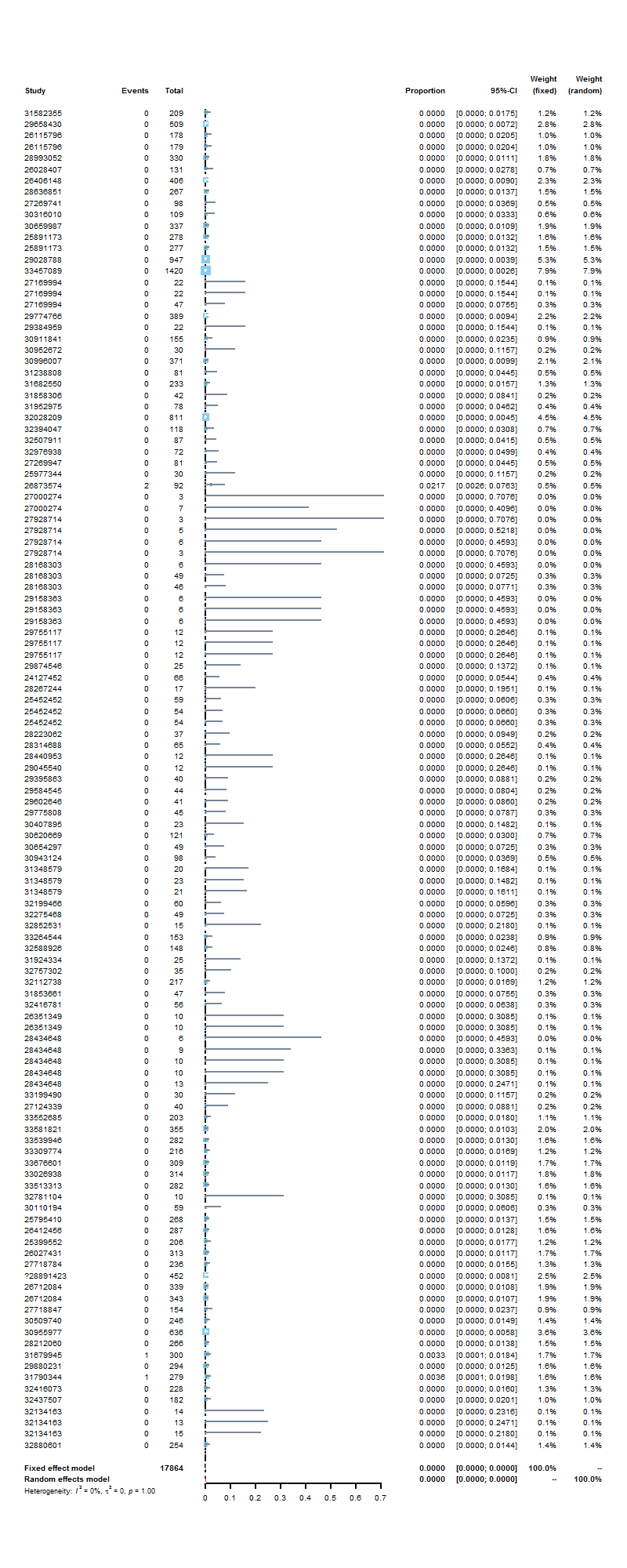


Forest plot of proportion of Erythema multiform with anti-CTLA-4 therapy


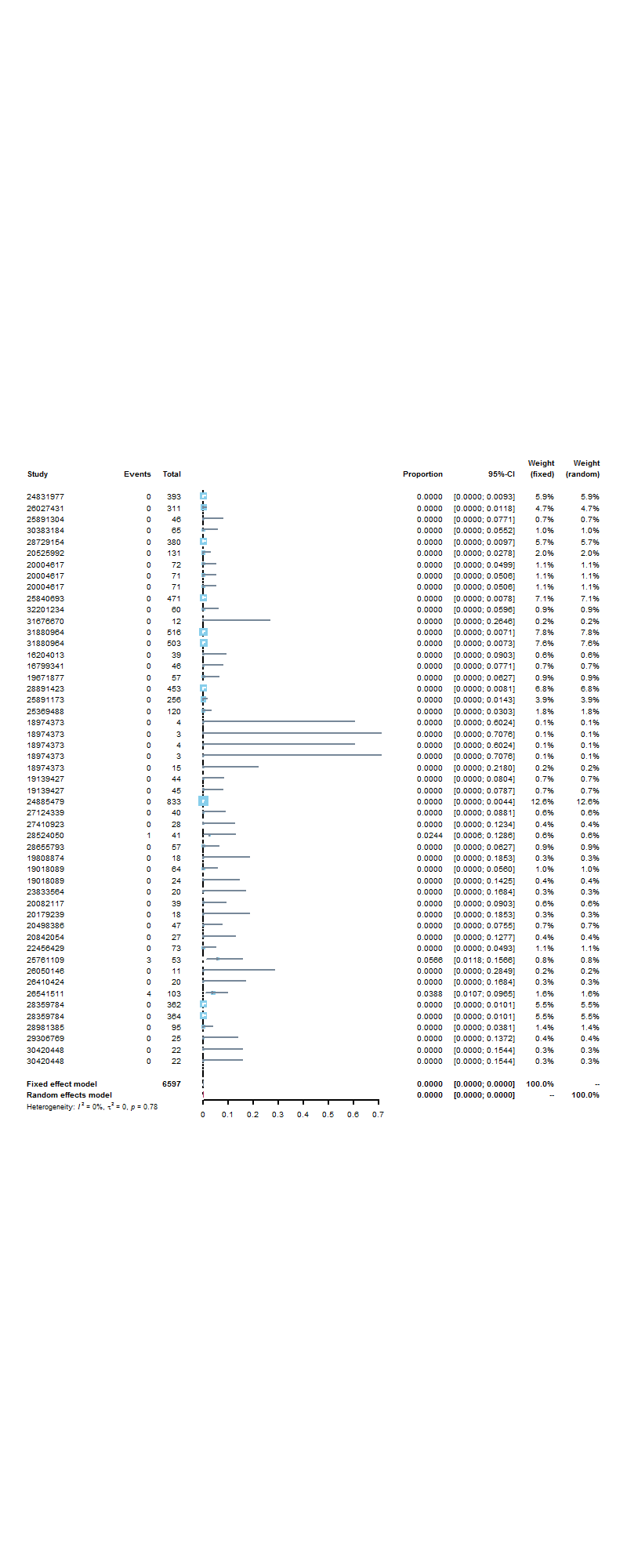


Forest plot of proportion of Erythema multiform with anti-PD-1 plus chemotherapy


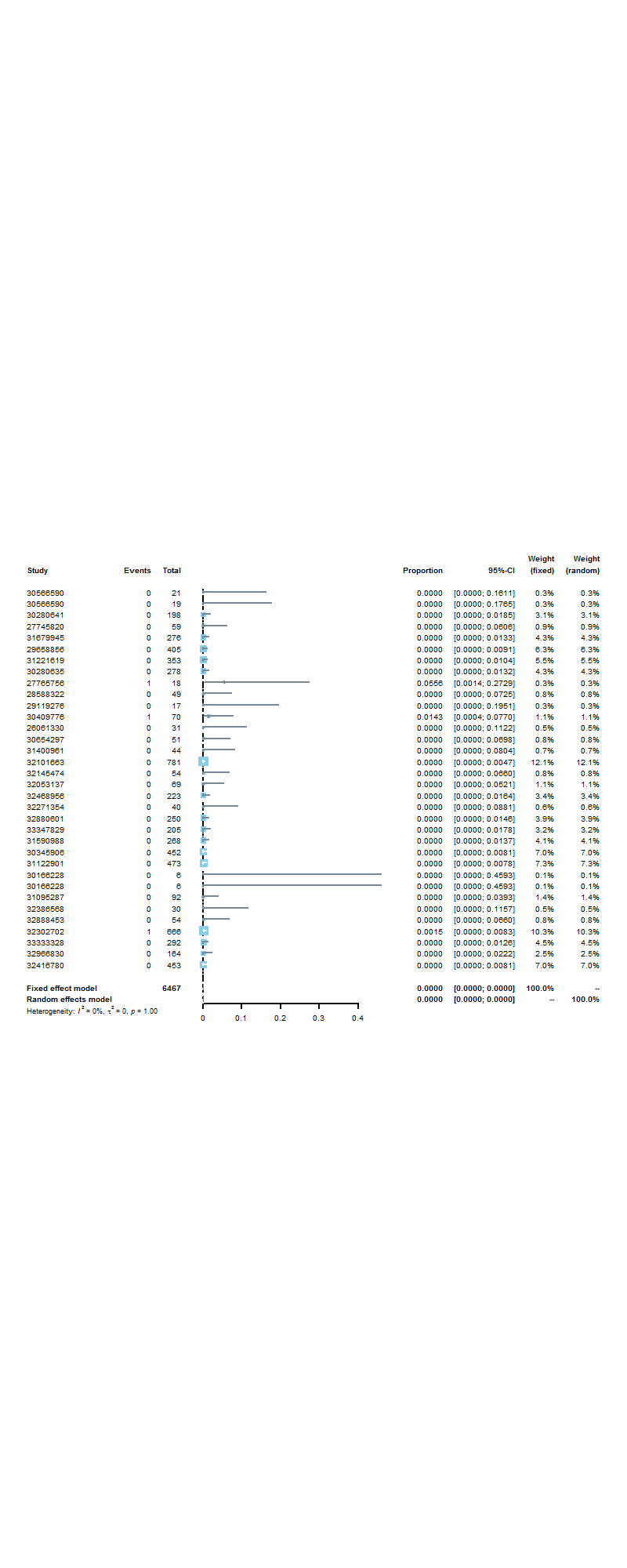


Forest plot of proportion of Erythema multiform with anti-CTLA-4 plus chemotherapy


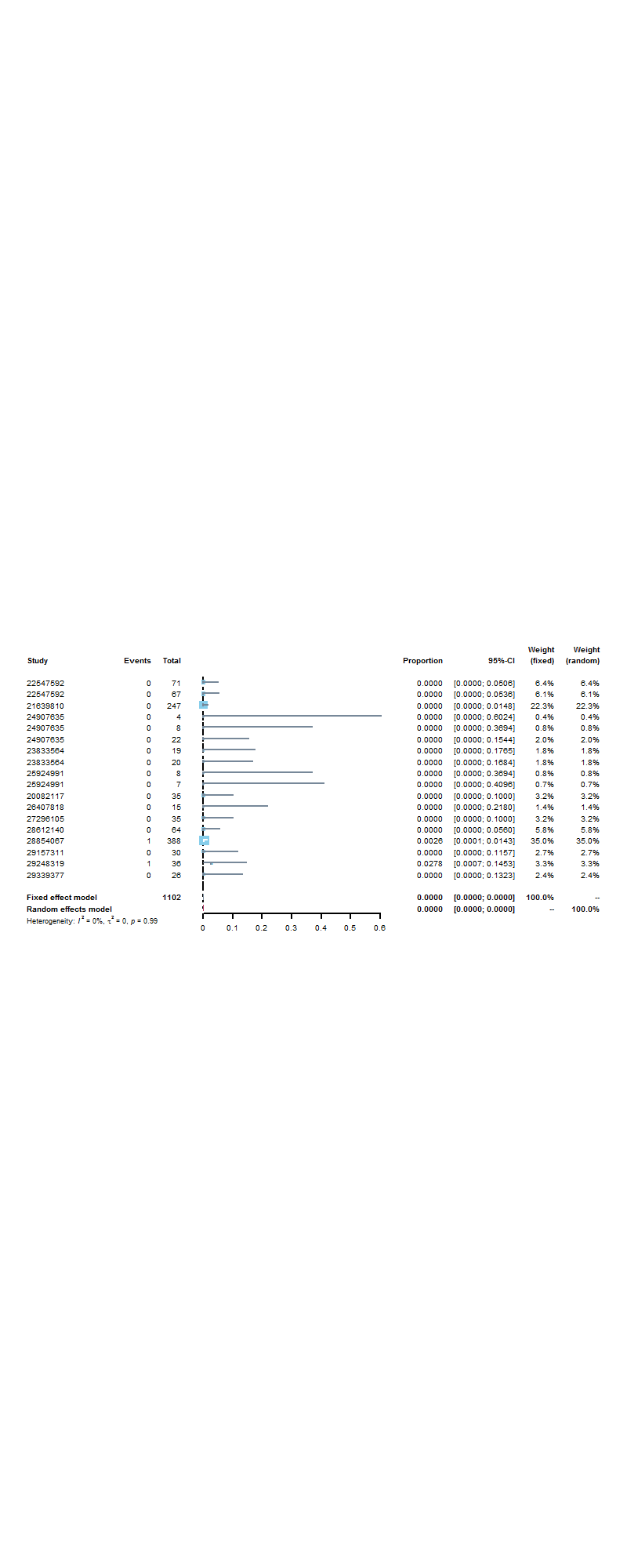


Forest plot of proportion of Erythema multiform with anti-PD-1/L1 plus EGFR monoclonal antibody


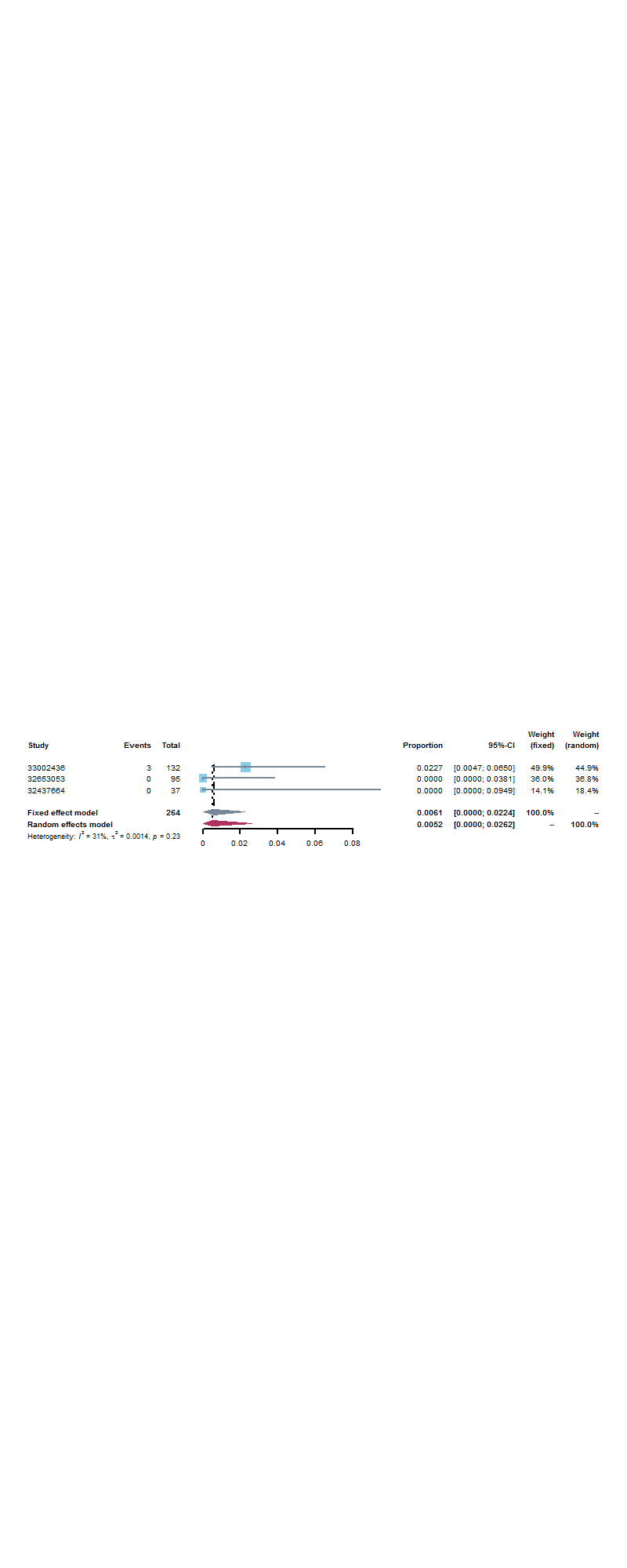


Forest plot of proportion of Erythema multiform with anti-PD-1/L1 plus VEGFR monoclonal antibody


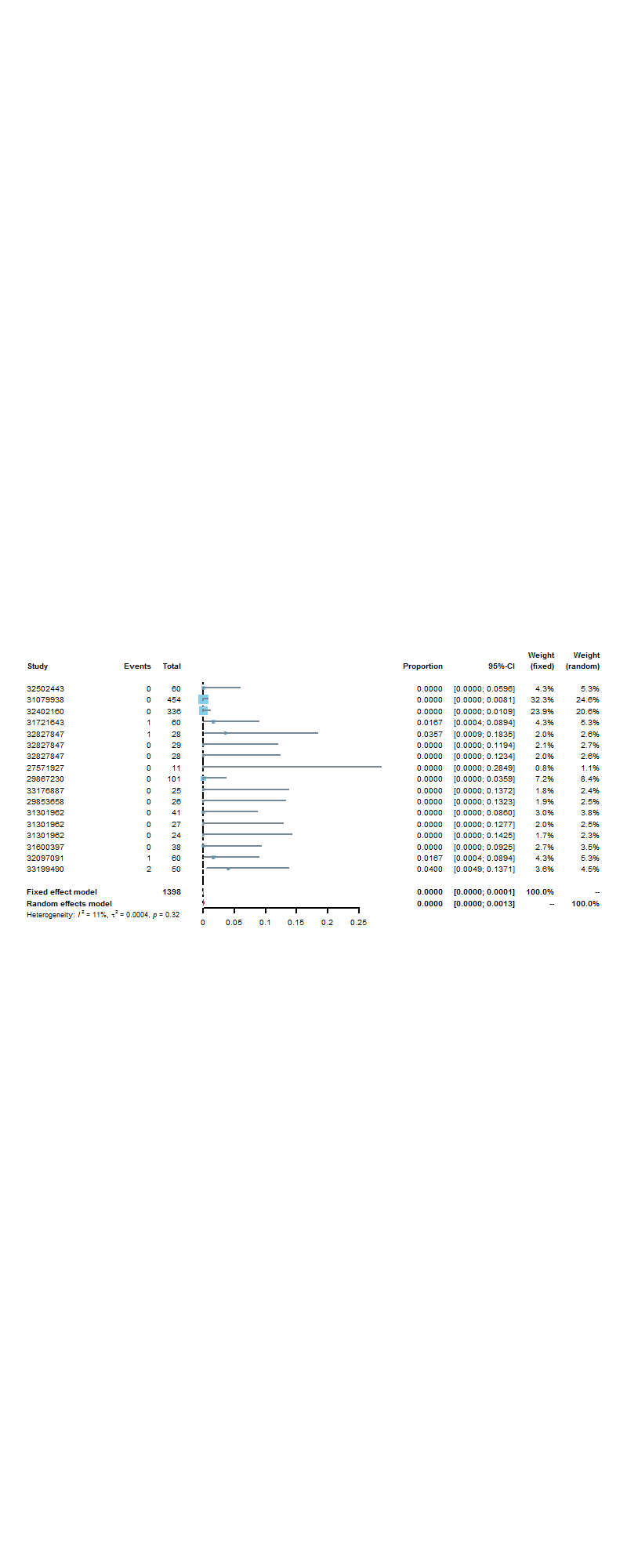


Forest plot of proportion of Erythema multiform with anti-PD-1/L1 plus VEGFR TKI


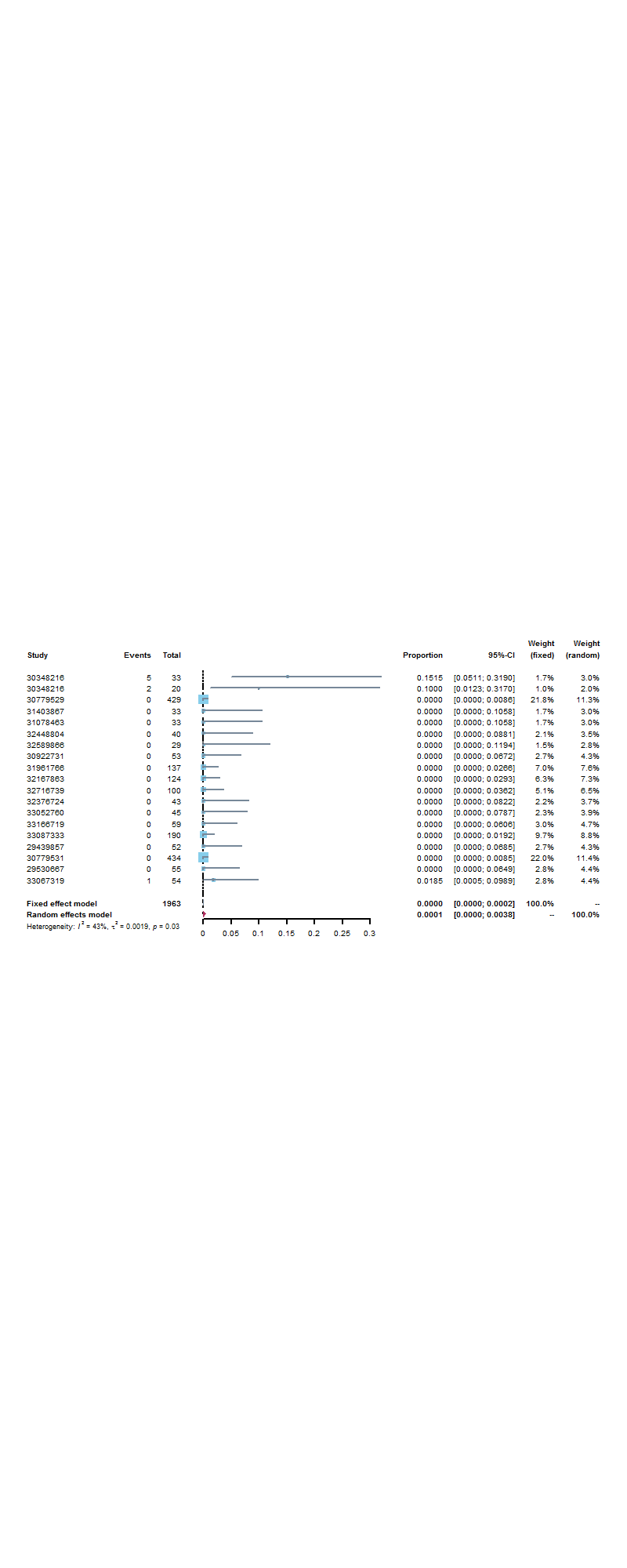


Forest plot of proportion of Bullous dermatitis with anti-PD-1 therapy


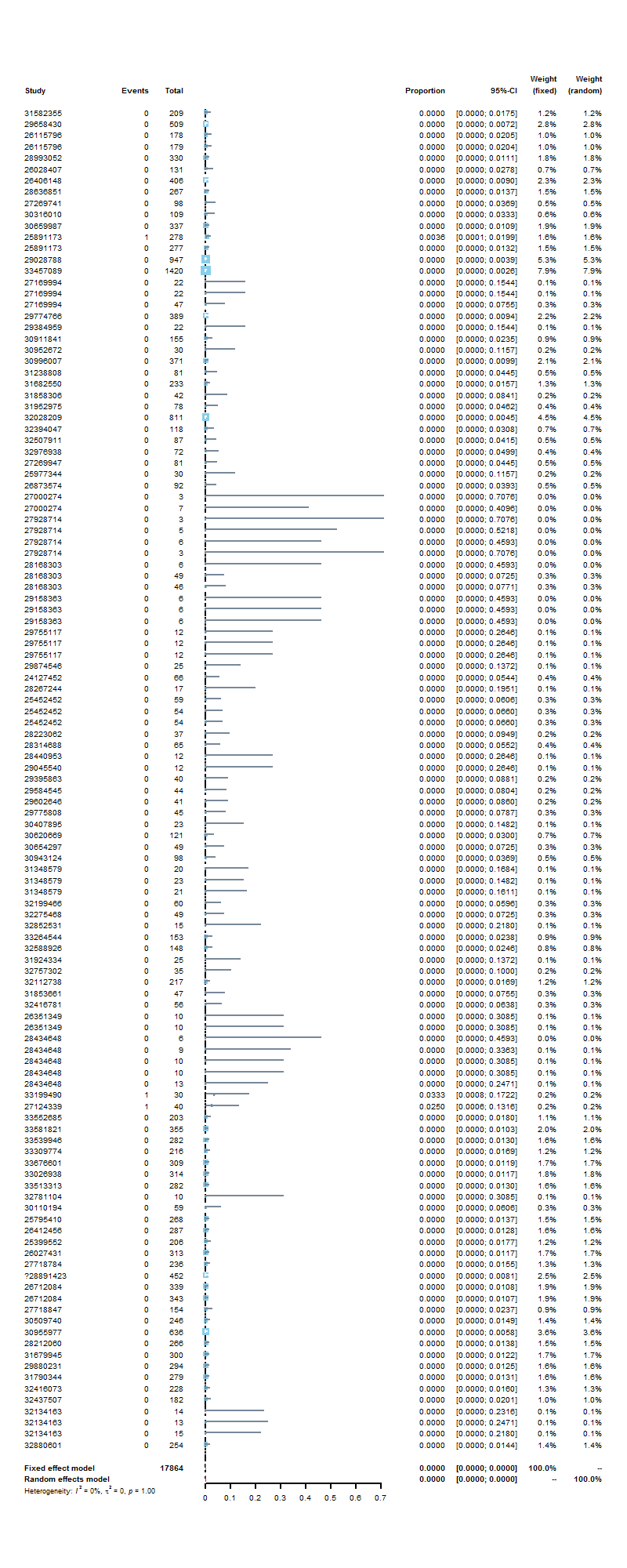


Forest plot of proportion of Bullous dermatitis with anti-PD-L1 therapy


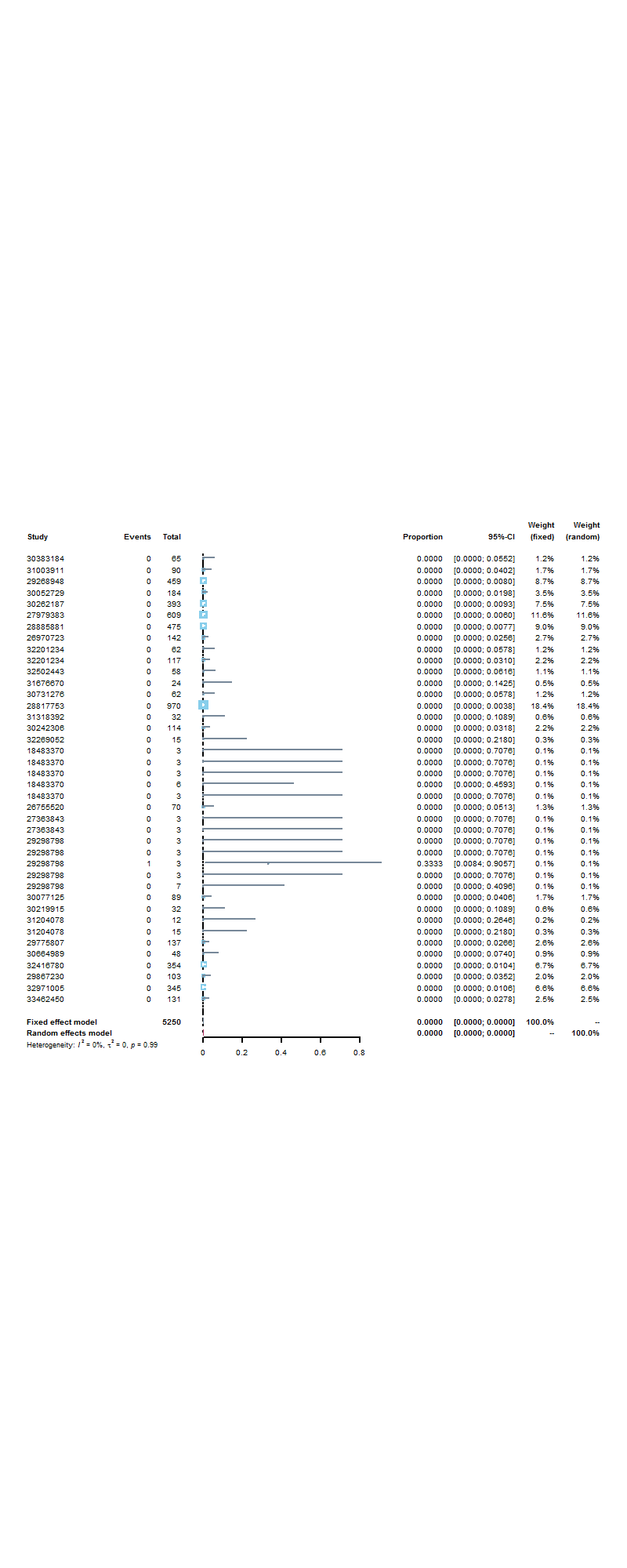


Forest plot of proportion of Bullous dermatitis with anti-PD-1/L1 plus VEGFR monoclonal antibody


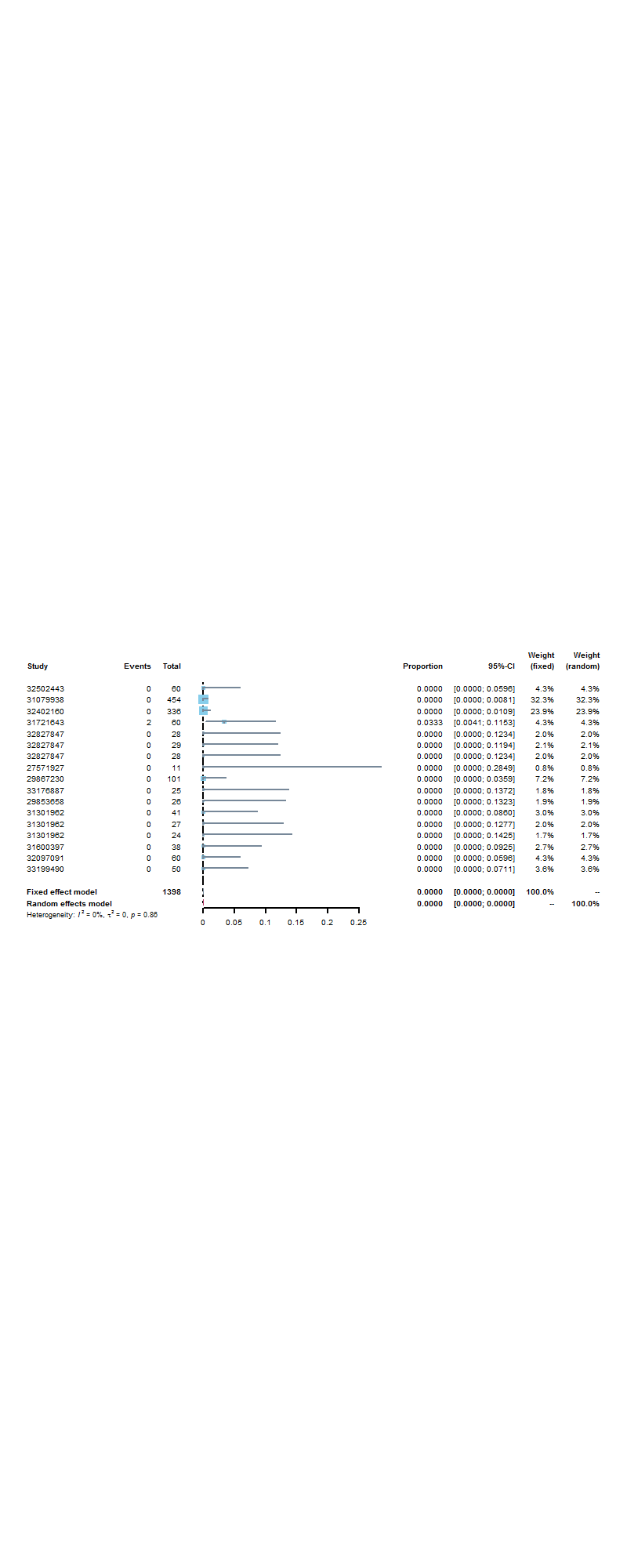


Forest plot of proportion of Drug eruption with anti-PD-1 therapy


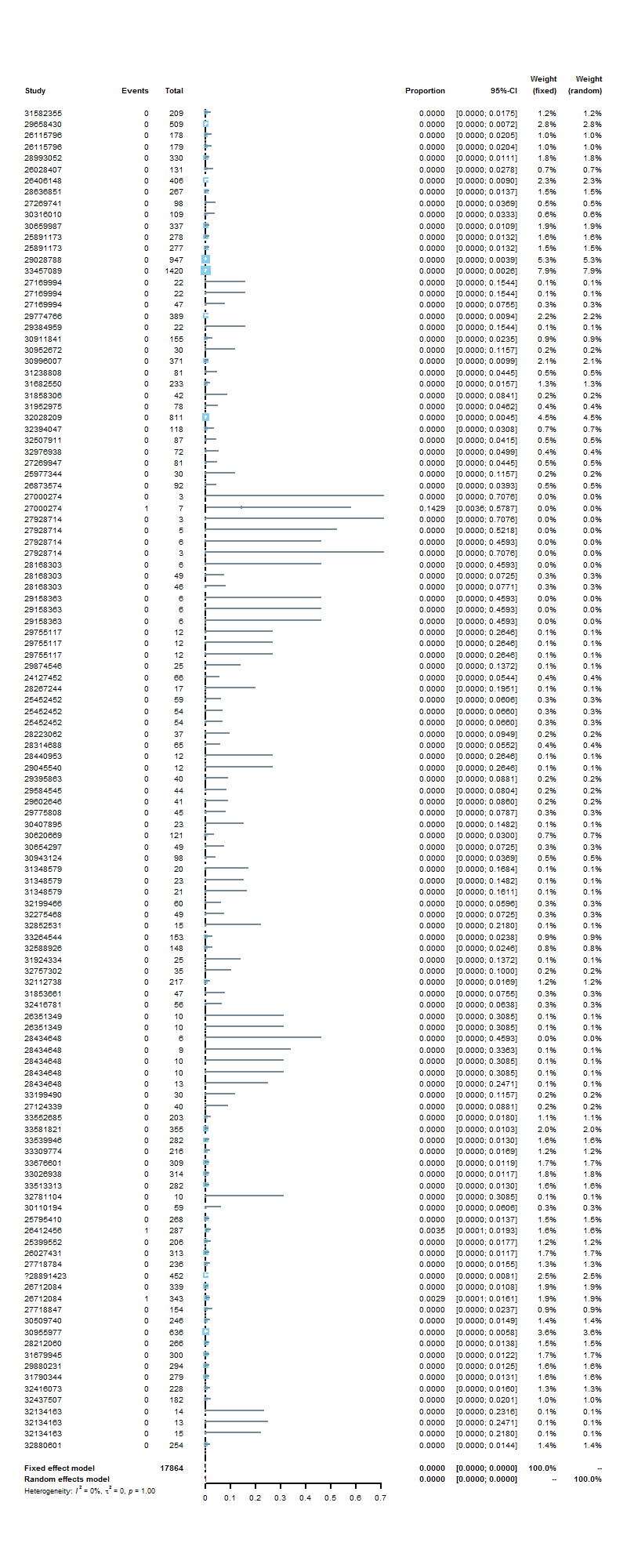


Forest plot of proportion of Drug eruption with anti-PD-L1 therapy


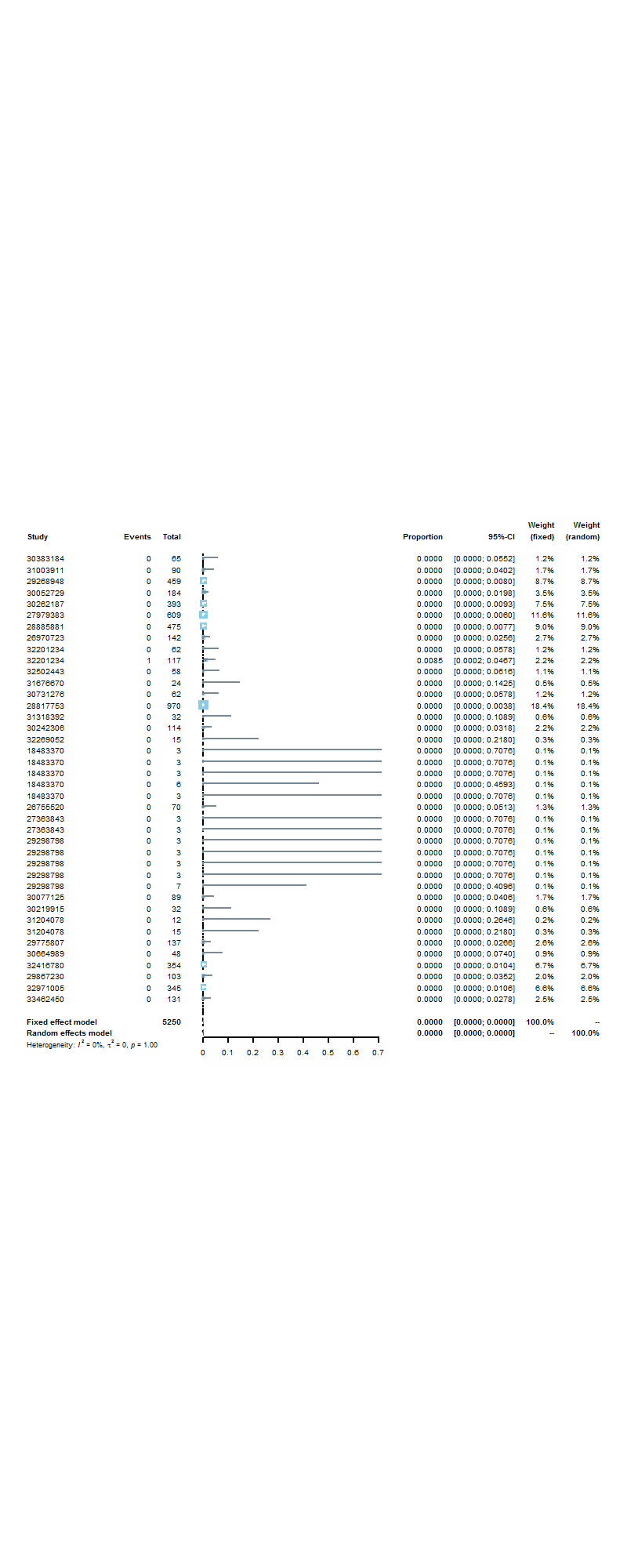


Forest plot of proportion of Drug eruption with anti-CTLA-4 therapy


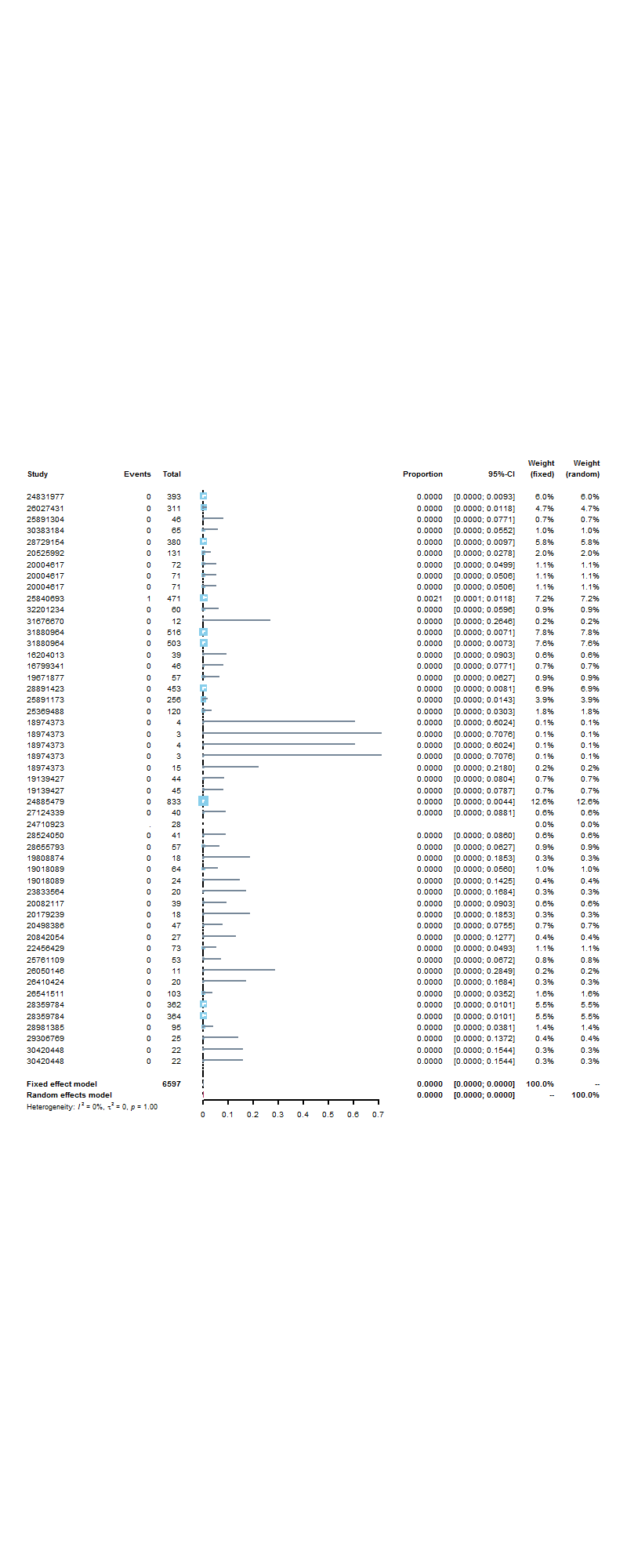


Forest plot of proportion of Drug eruption with anti-PD-1/L1 plus chemotherapy


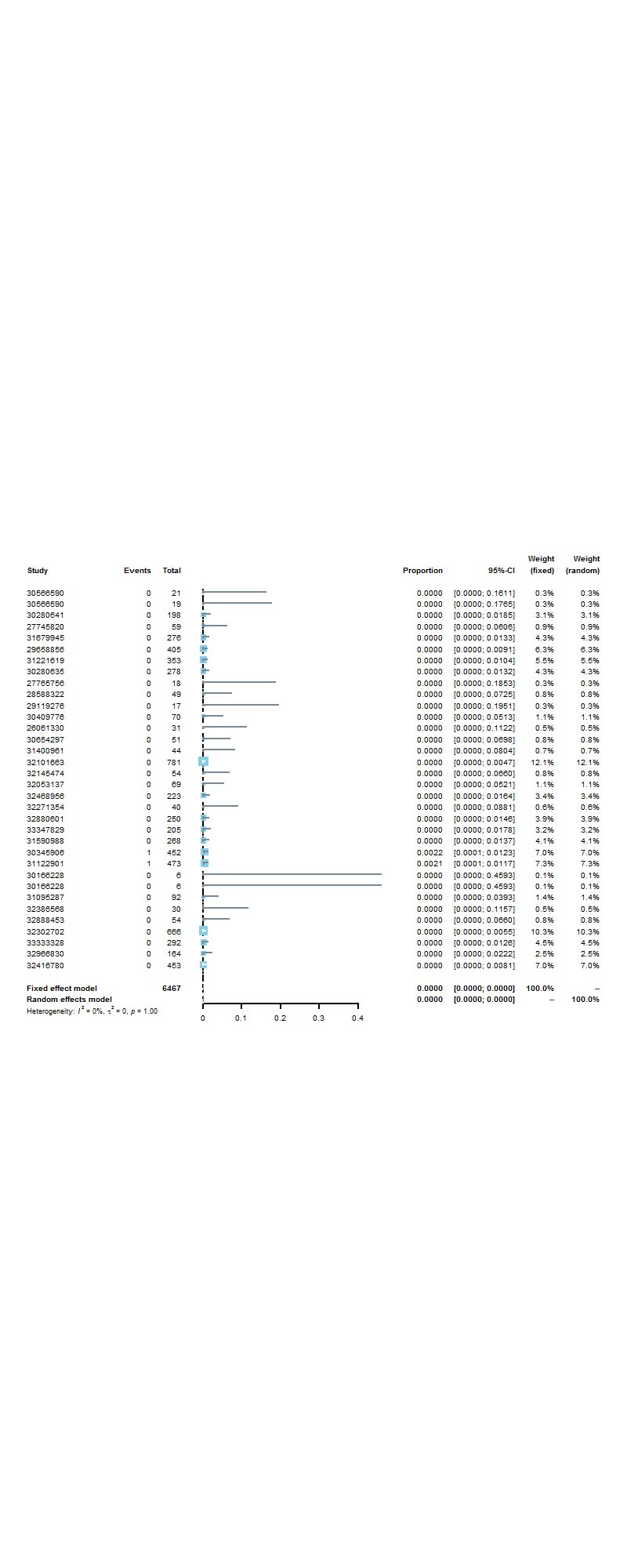


Forest plot of proportion of Drug eruption with anti-CTLA-4 plus chemotherapy


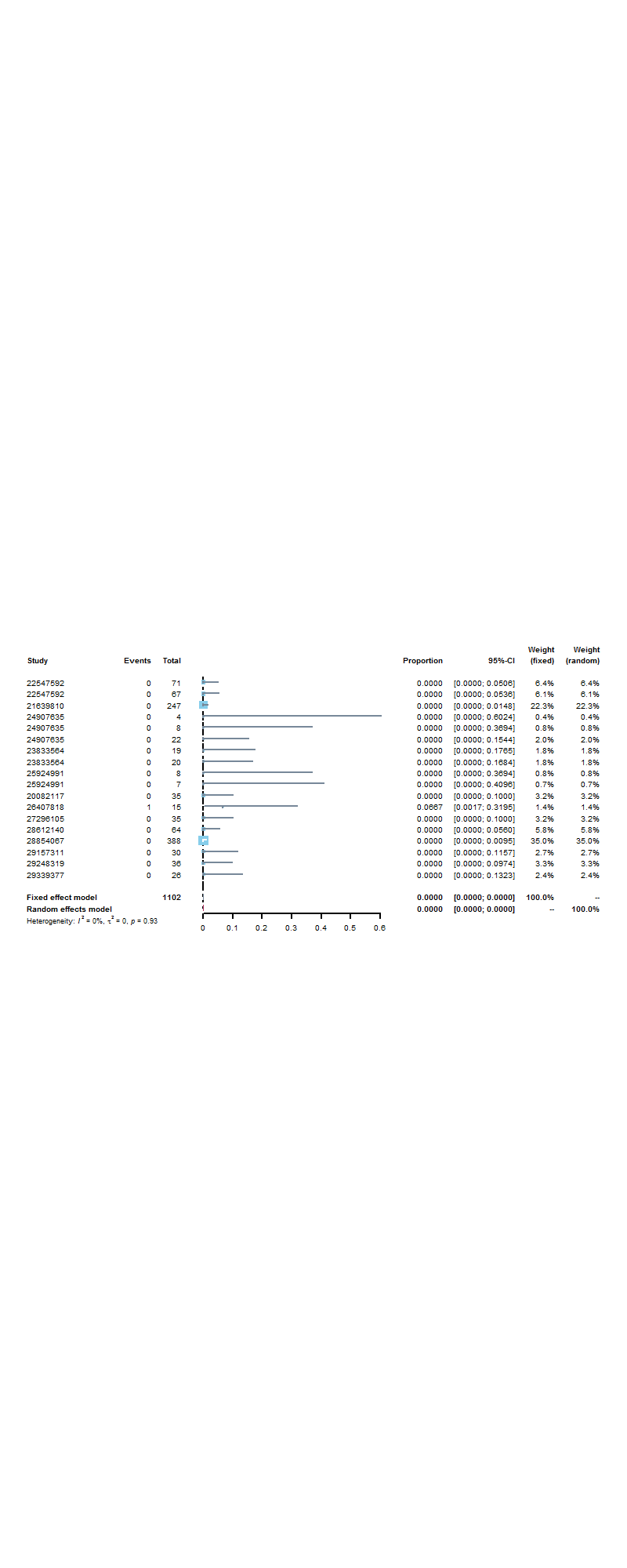


Forest plot of proportion of Drug eruption with anti-PD-1/L1 plus VEGFR TKI


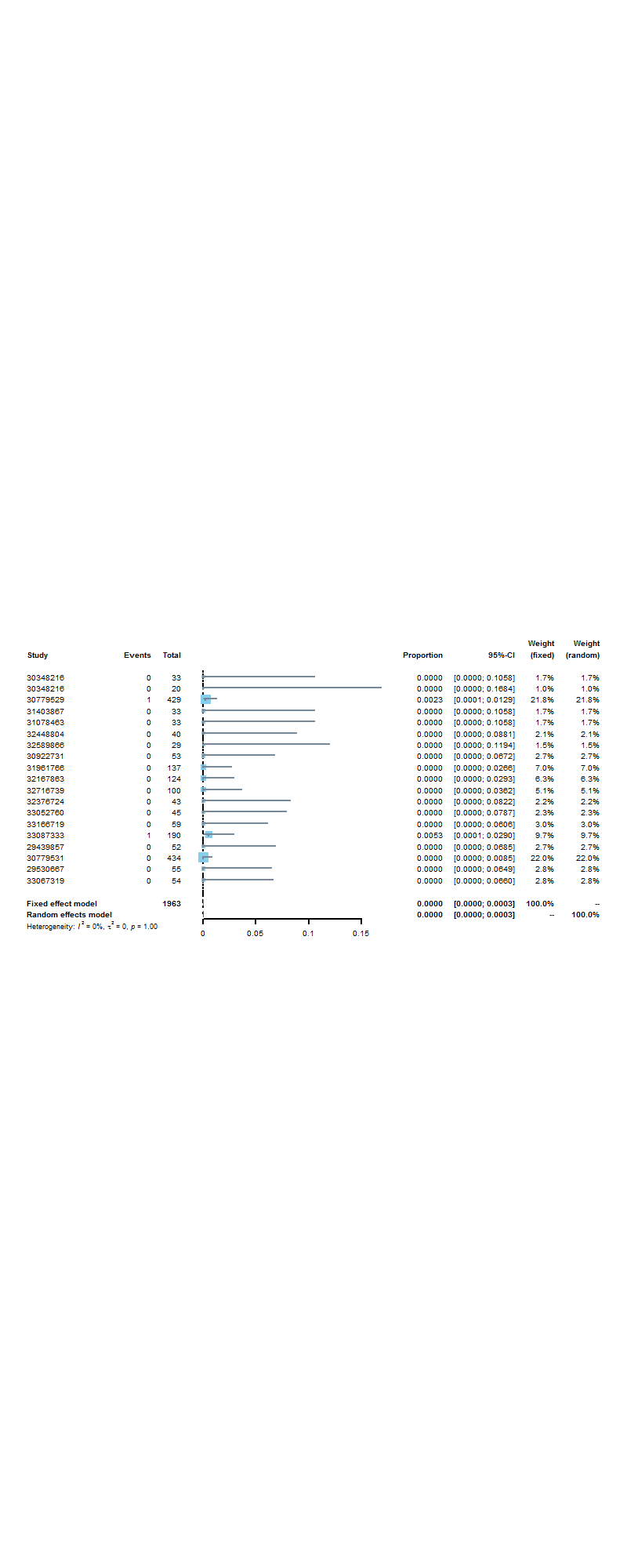


Forest plot of proportion of Acneiform rash with anti-PD-1 therapy


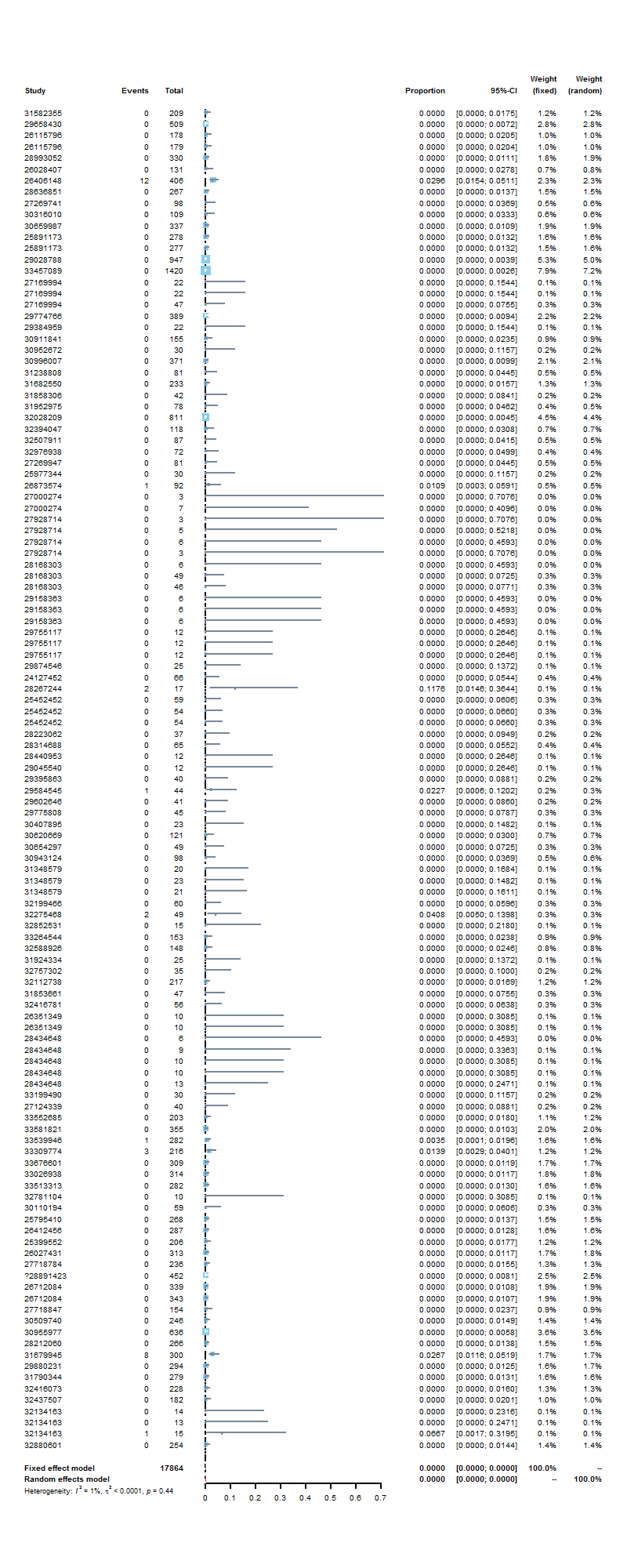


Forest plot of proportion of Acneiform rash with anti-PD-L1 therapy


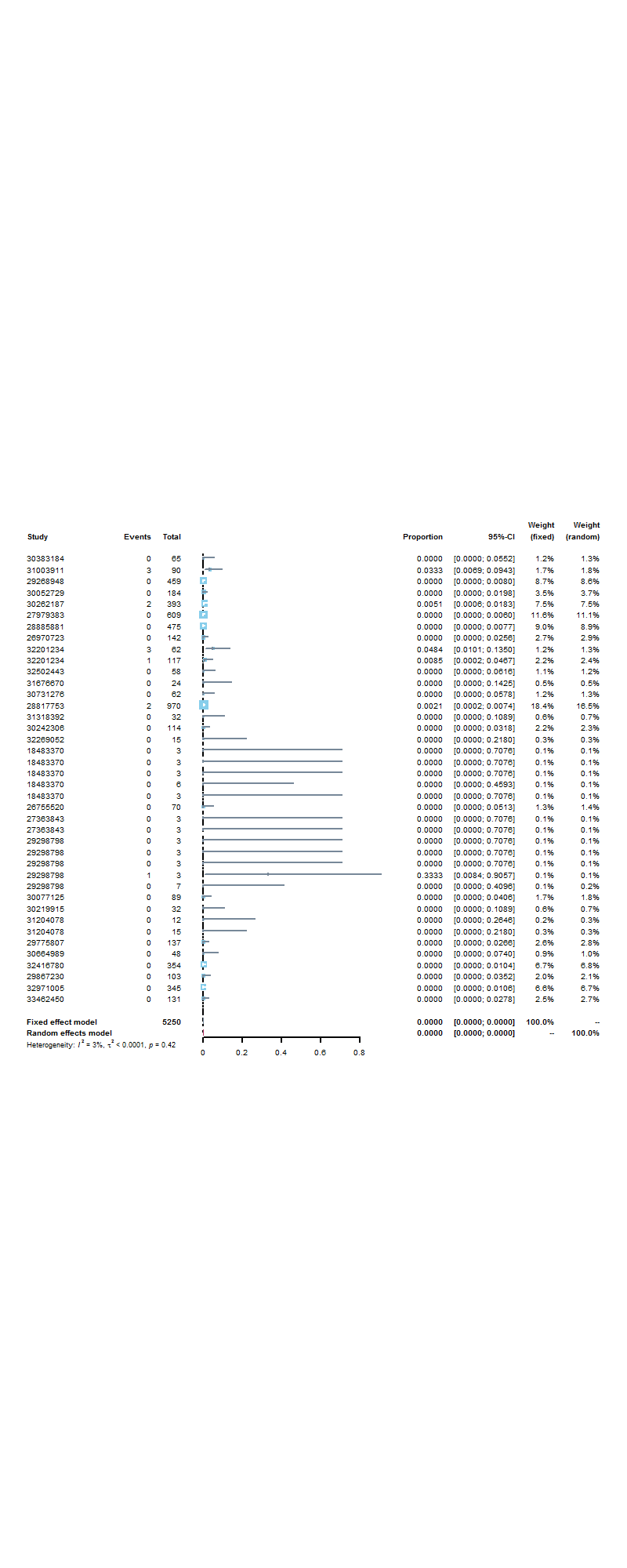


Forest plot of proportion of Acneiform rash with anti-CTLA-4 therapy


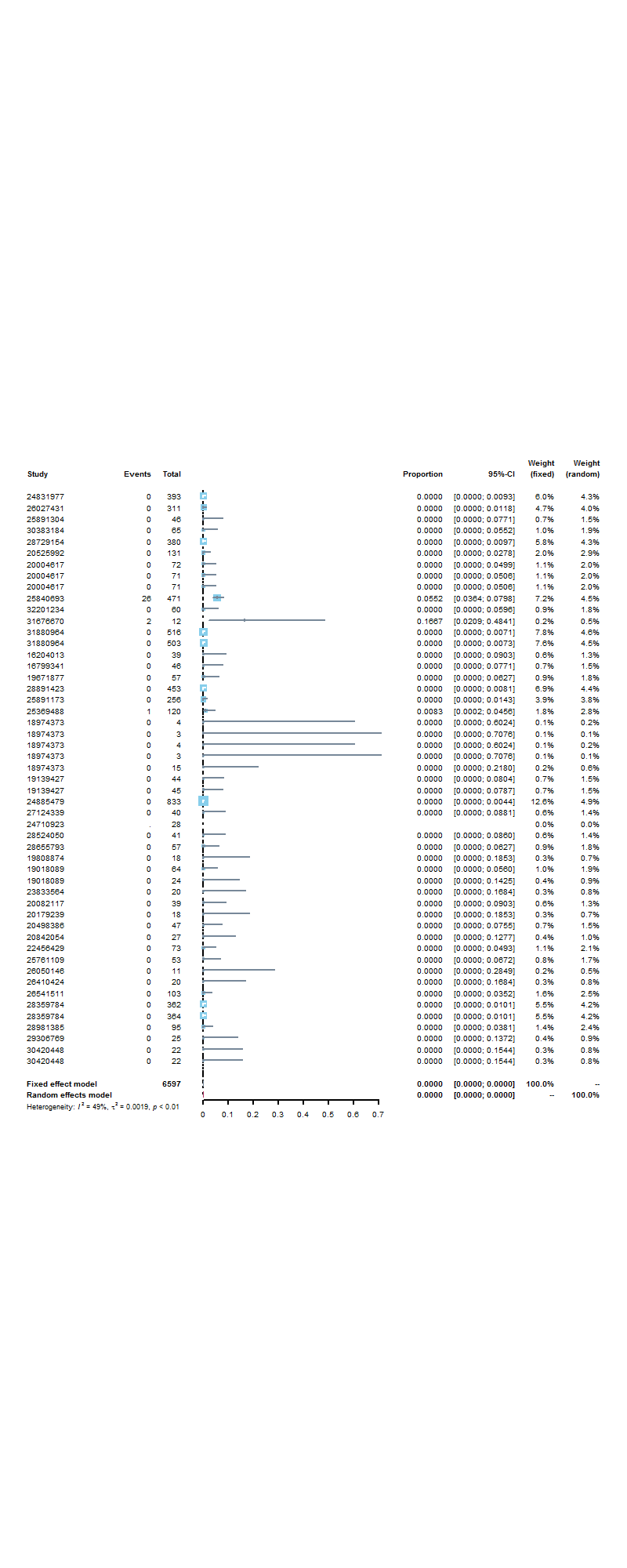


Forest plot of proportion of Acneiform rash with anti-PD-1/L1 plus chemotherapy


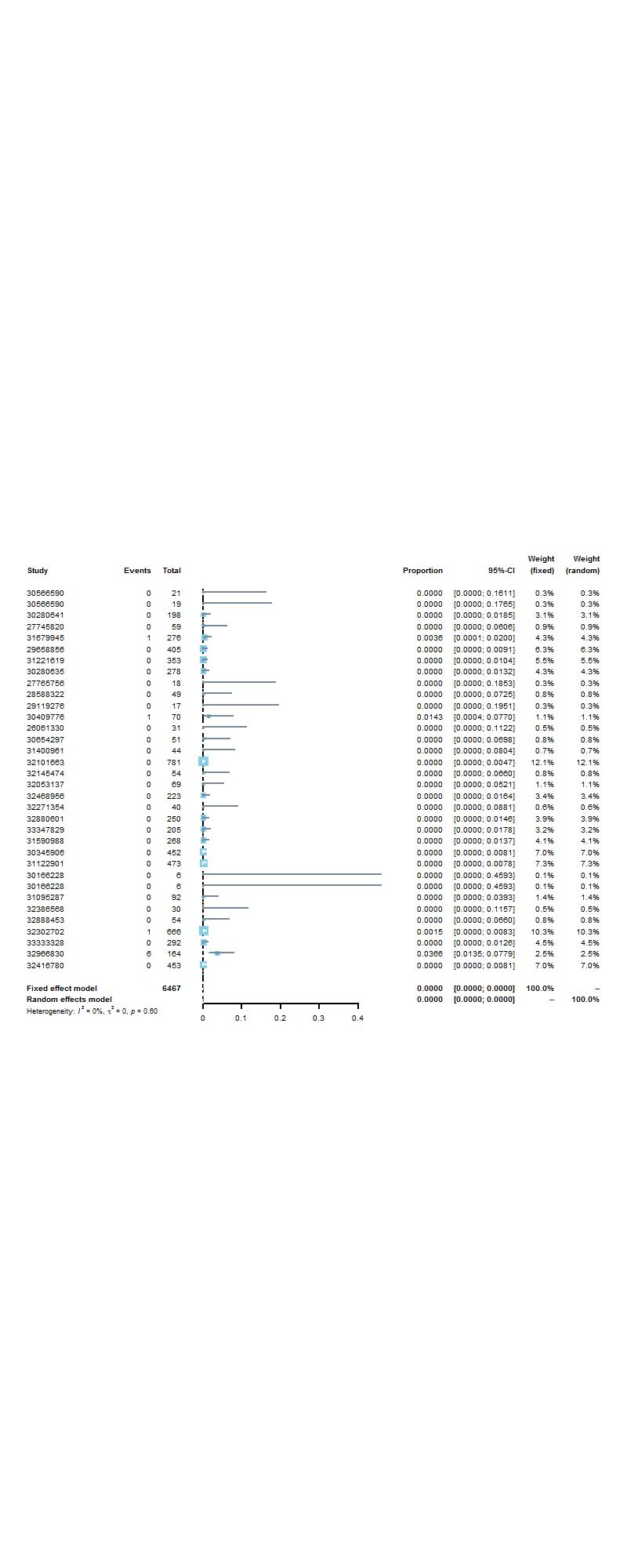


Forest plot of proportion of Acneiform rash with anti-CTLA-4 plus chemotherapy


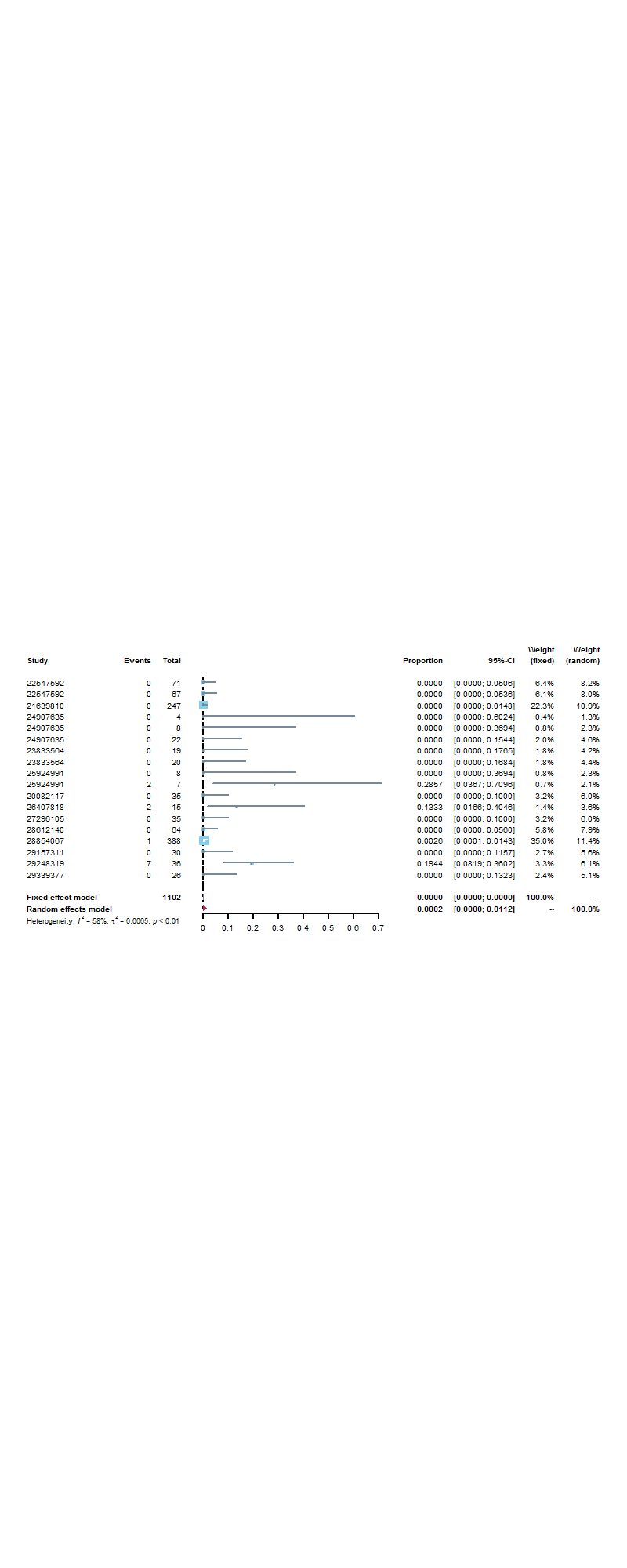


Forest plot of proportion of Acneiform rash with anti-PD-1/L1 plus VEGFR monoclonal antibody


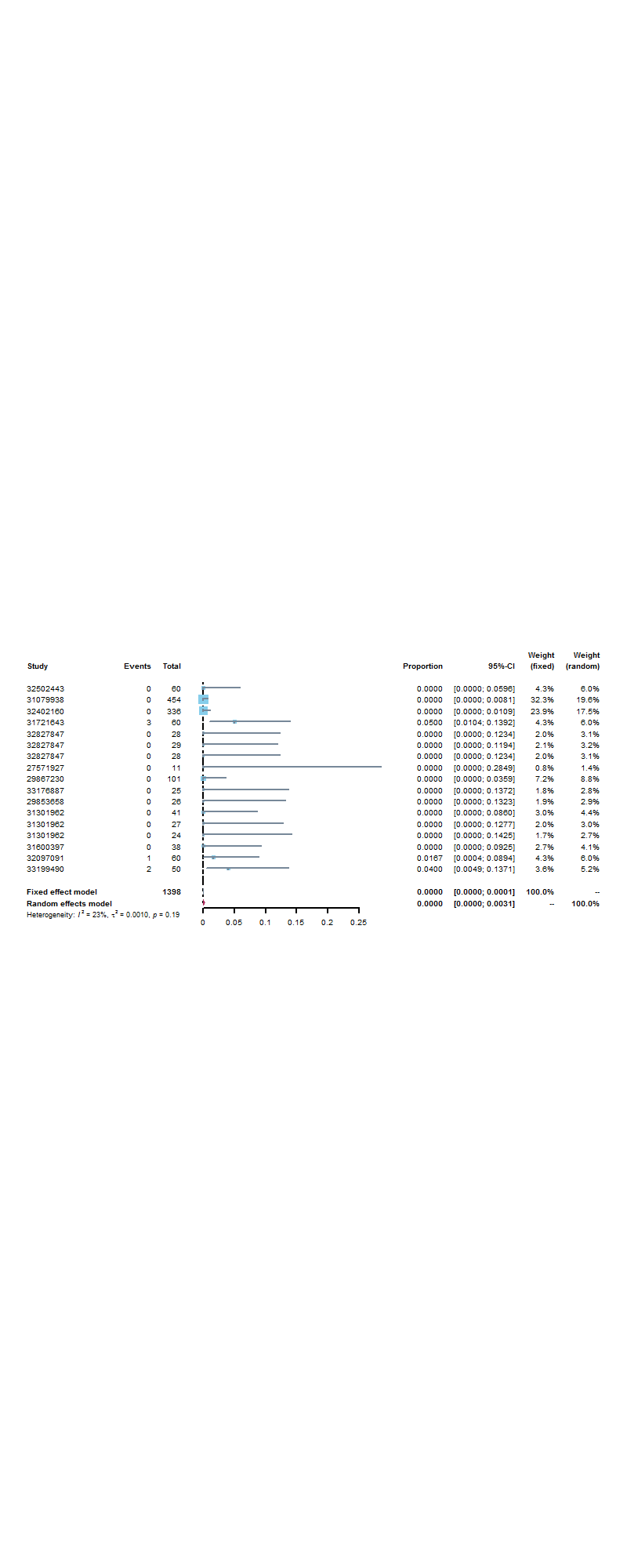


Forest plot of proportion of Acneiform rash with anti-PD-1/L1 plus VEGFR TKI


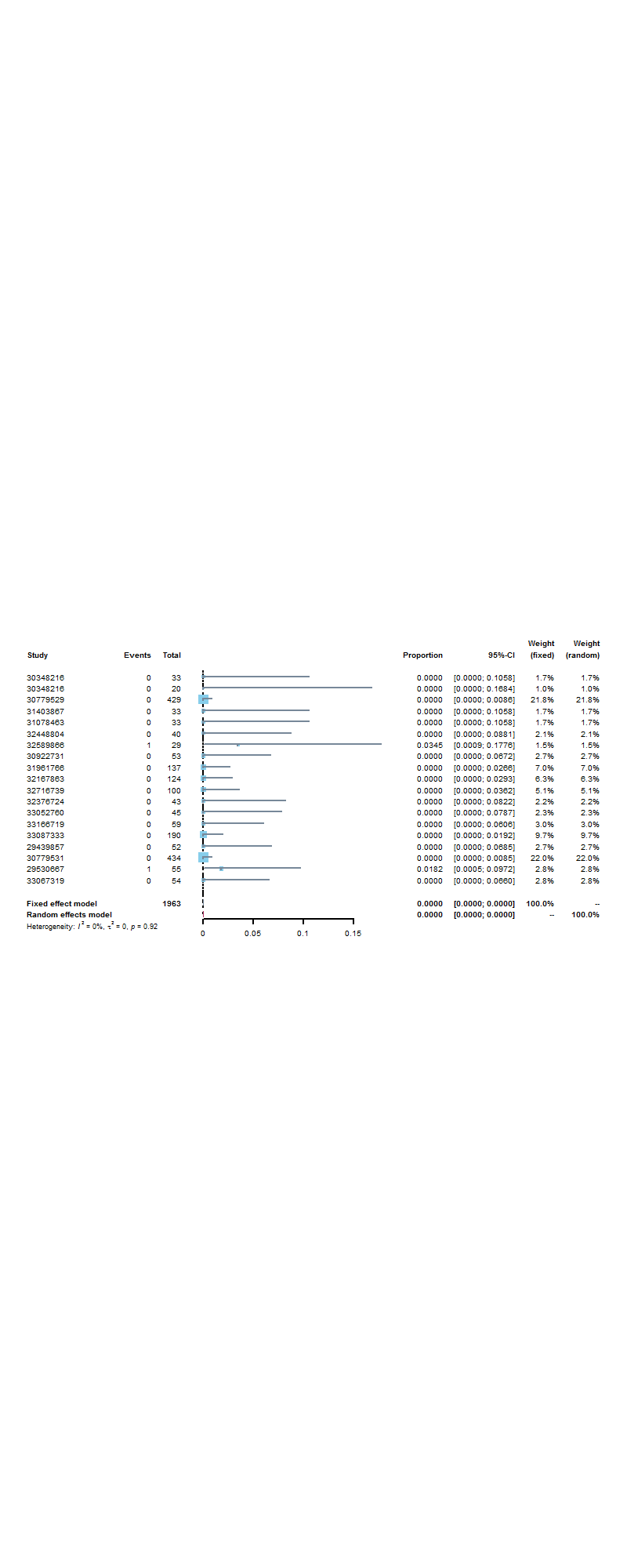


Forest plot of proportion of Skin exfoliation with anti-PD-1 therapy


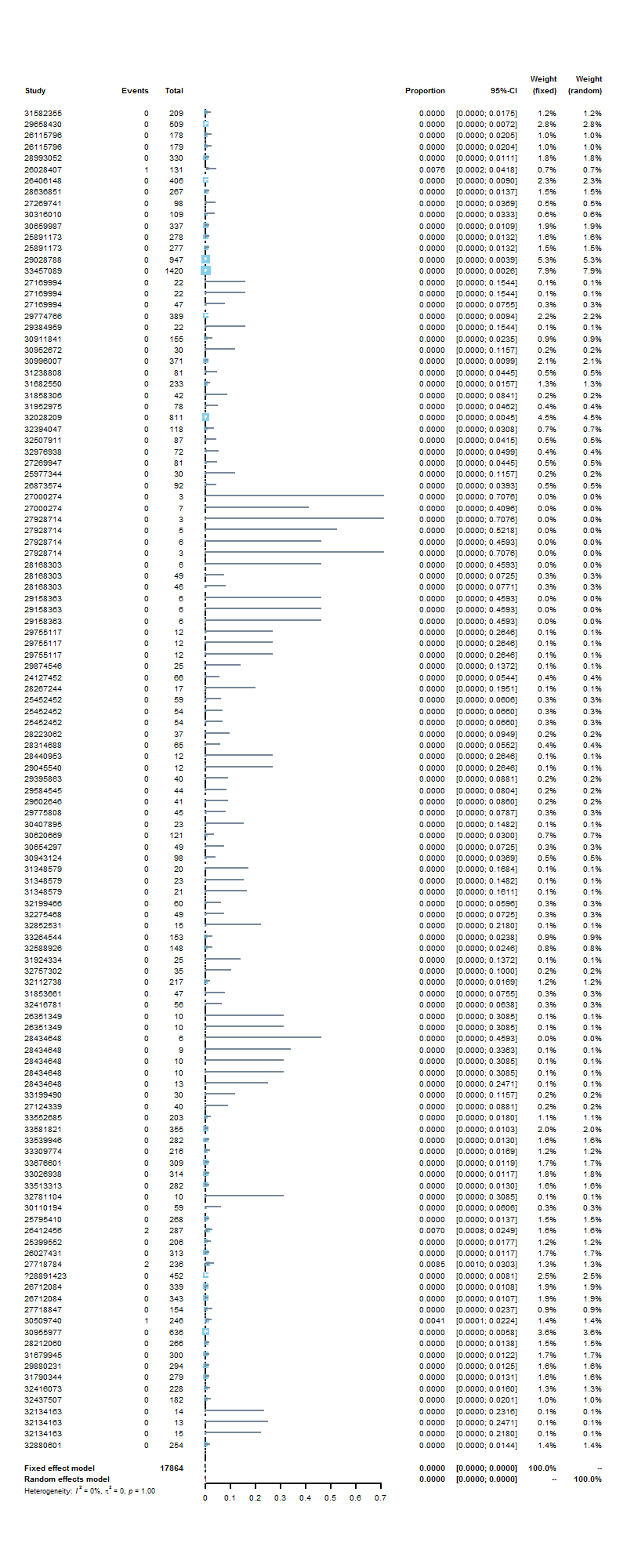


Forest plot of proportion of Skin exfoliation with anti-PD-L1 therapy


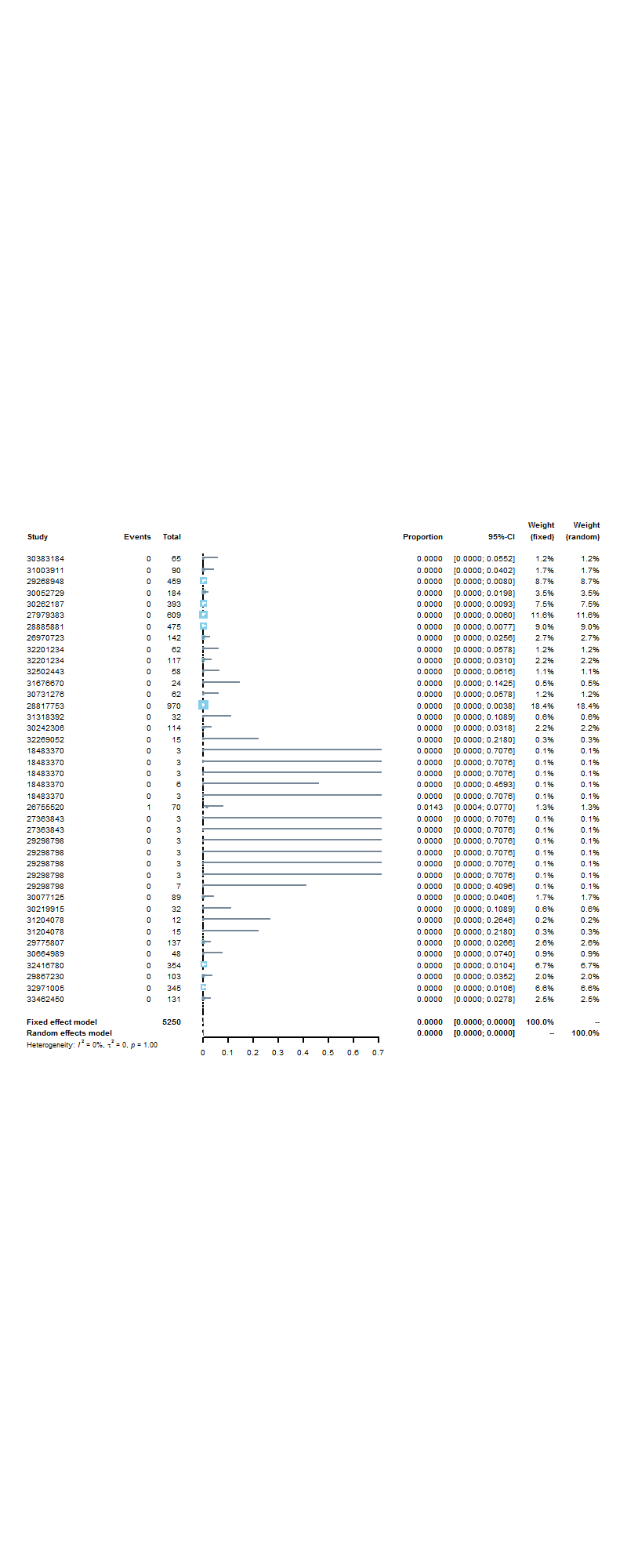


Forest plot of proportion of Skin exfoliation with anti-PD-1/L1 plus anti-CTLA-4 therapy


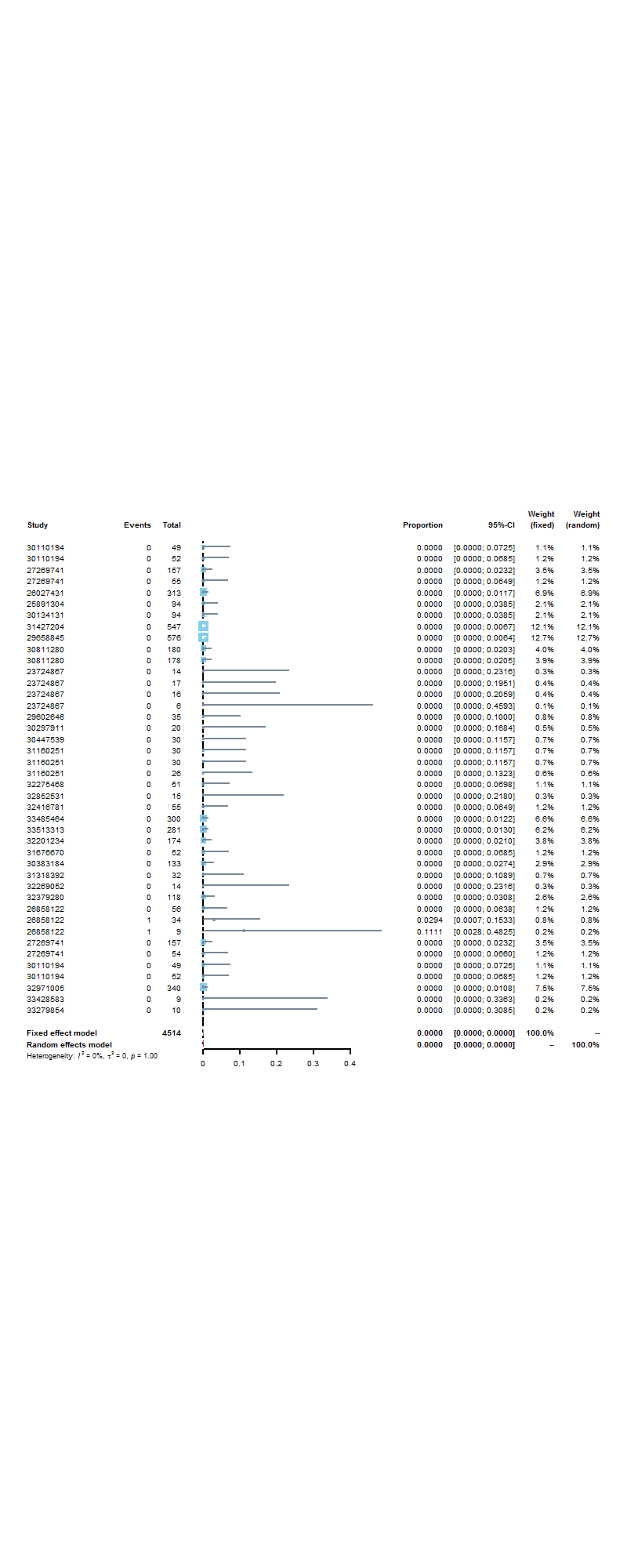


Forest plot of proportion of Skin exfoliation with anti-PD-1/L1 plus chemotherapy


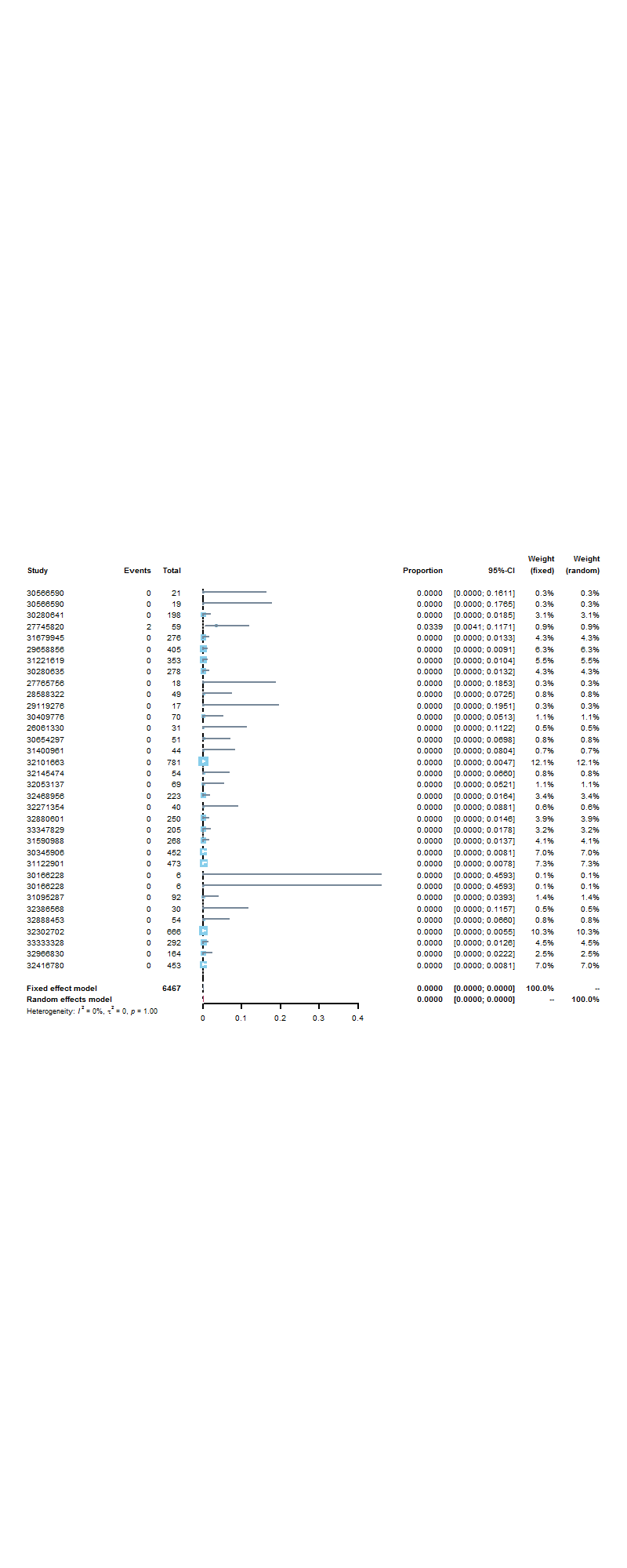


Forest plot of proportion of Skin exfoliation with anti-PD-1/L1 plus EGFR TKI


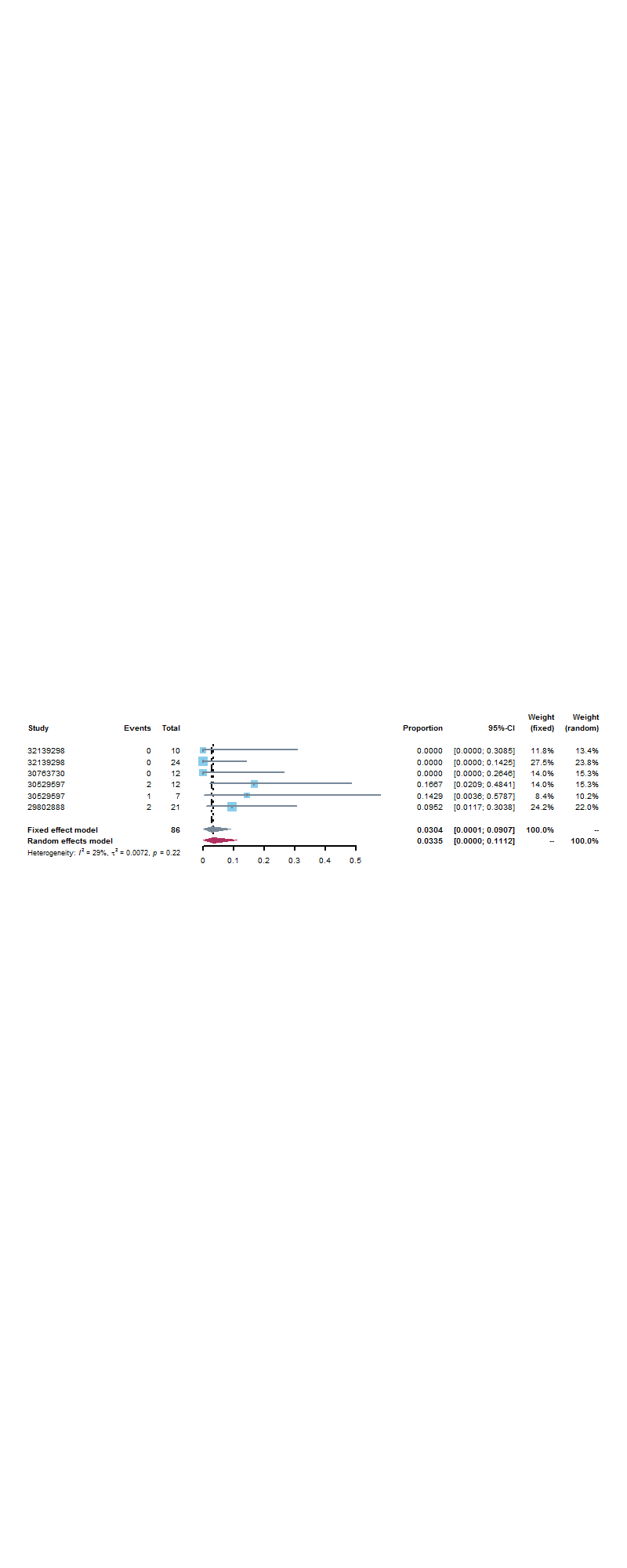


Forest plot of proportion of Skin exfoliation with anti-PD-1/L1 plus VEGFR TKI


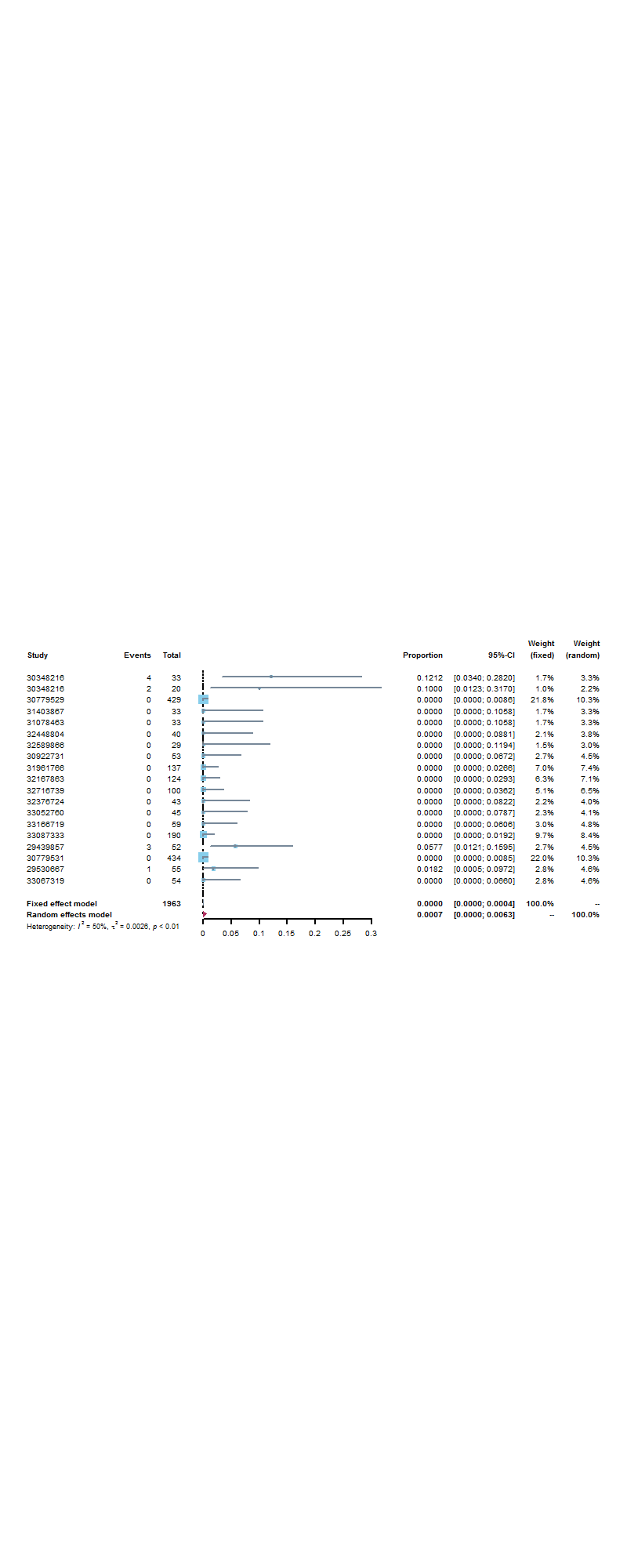


Forest plot of proportion of Skin ulceration with anti-PD-1 therapy


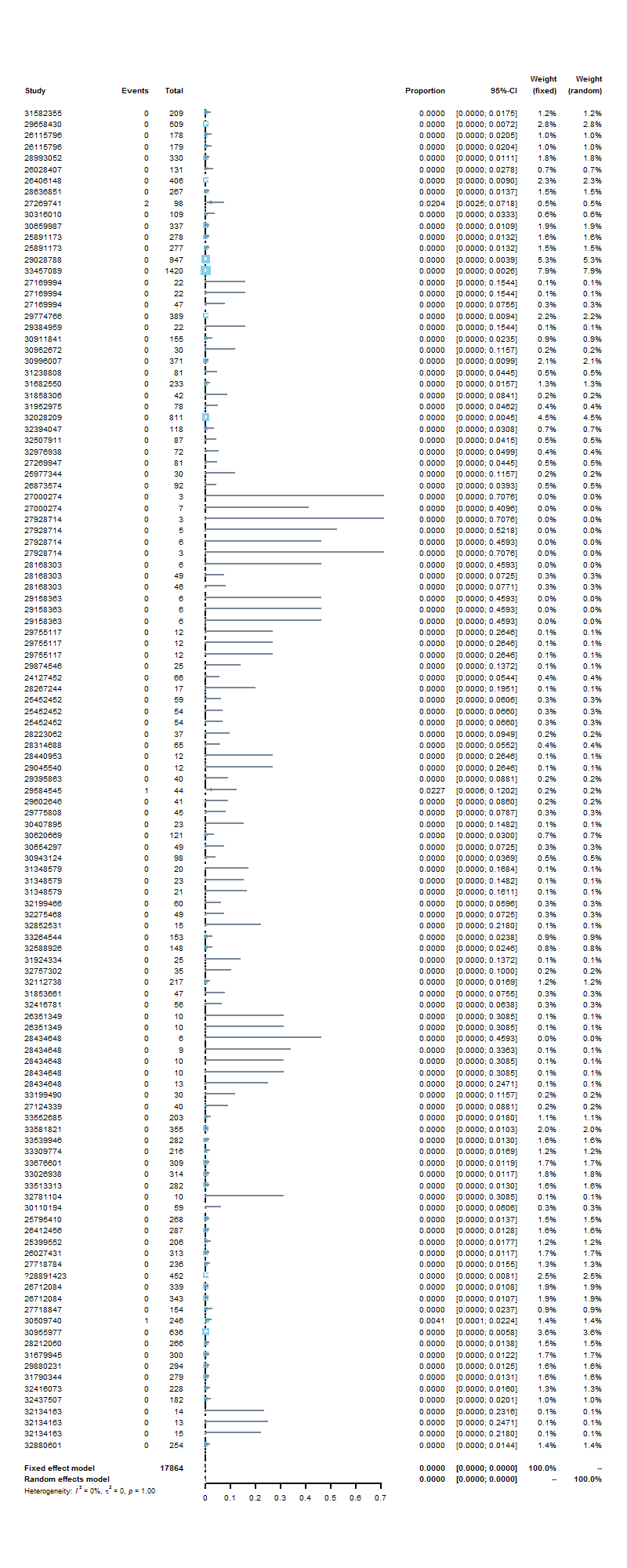


Forest plot of proportion of Skin ulceration with anti-CTLA-4 therapy


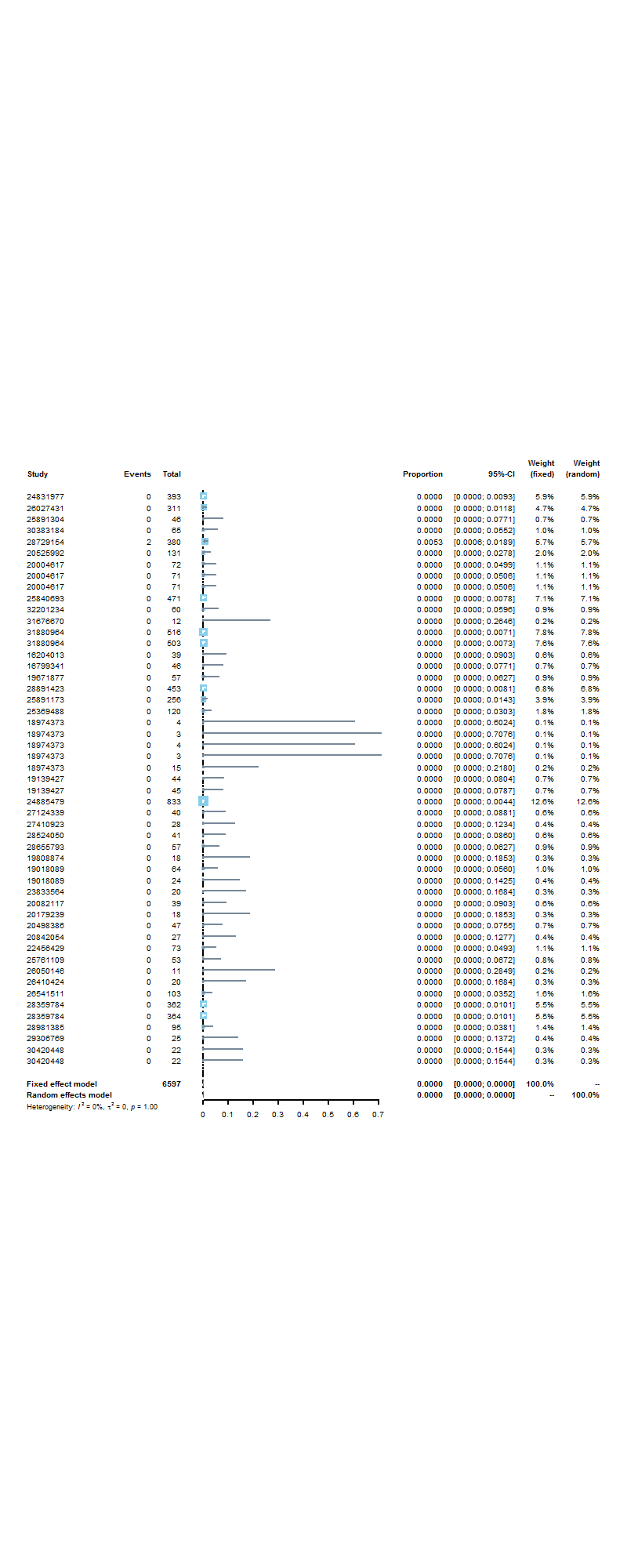


Forest plot of proportion of Skin ulceration with anti-CTLA-4 plus chemotherapy


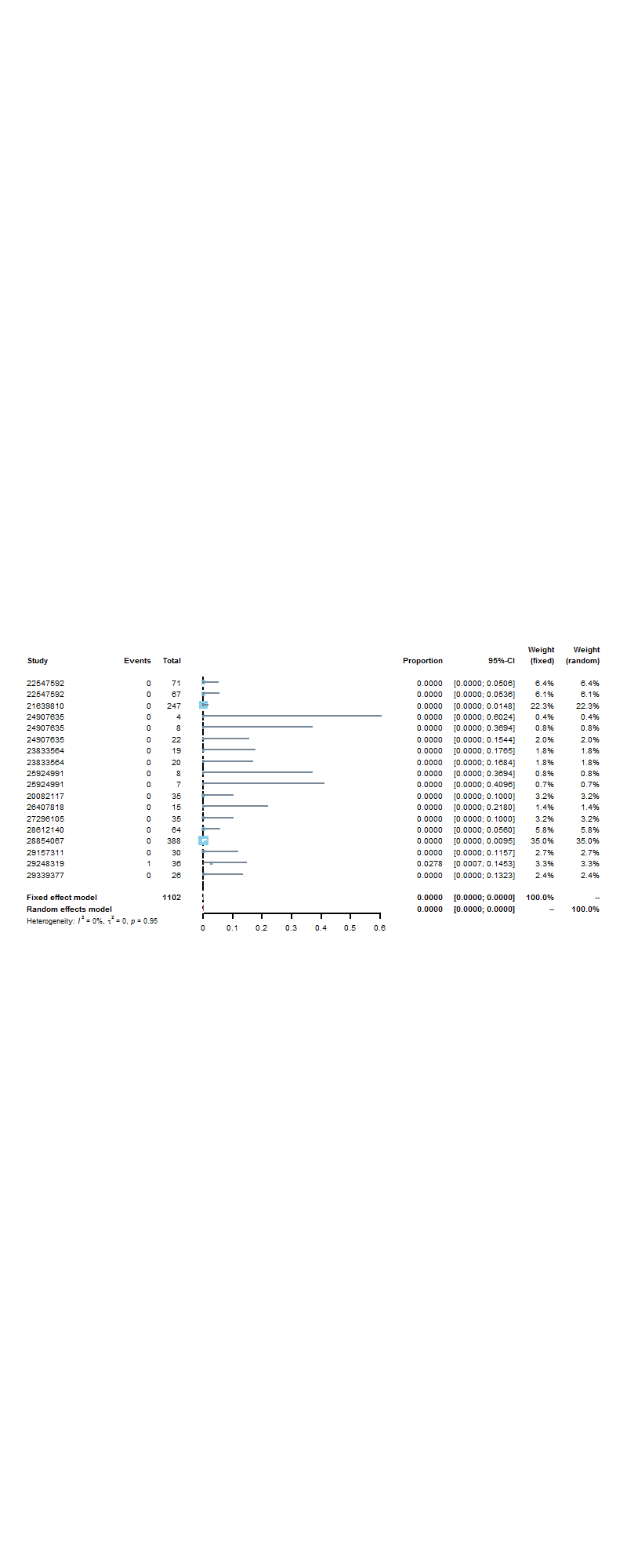


Forest plot of proportion of Skin ulceration with anti-PD-1/L1 plus VEGFR monoclonal antibody


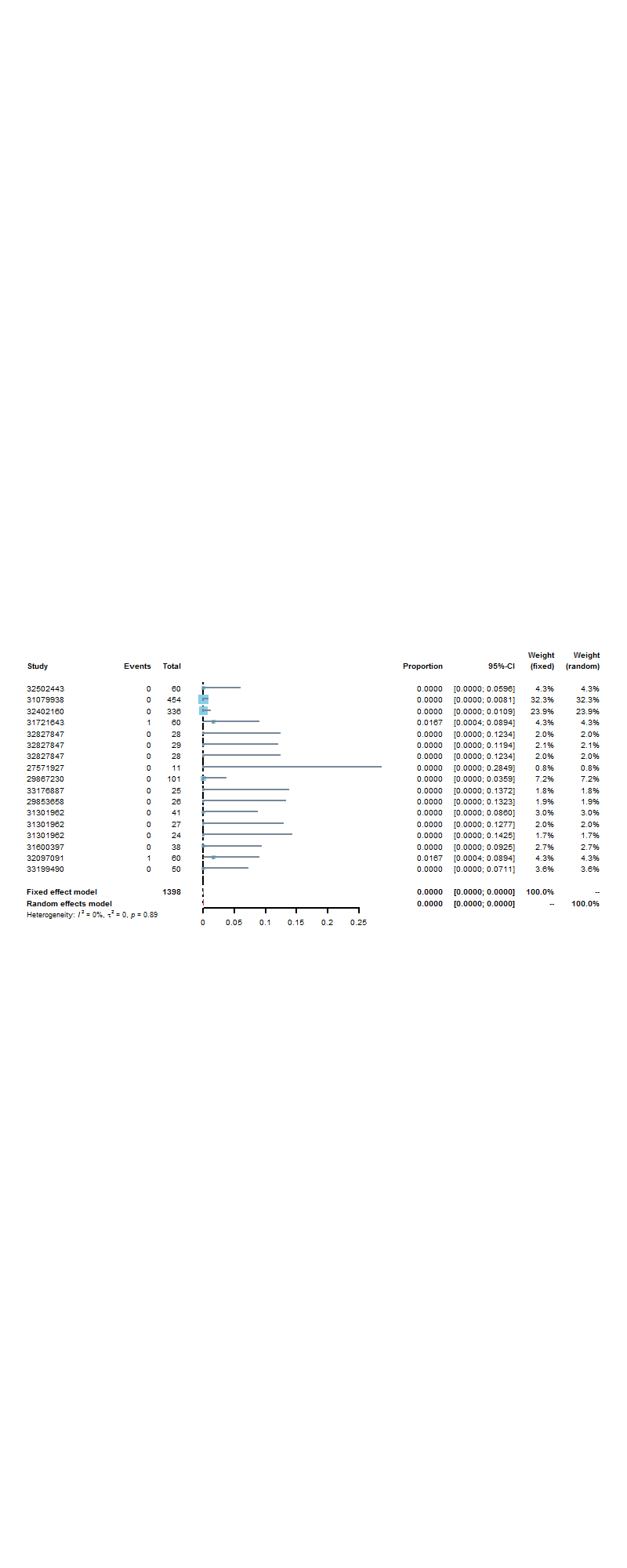


Forest plot of proportion of Skin ulceration with anti-PD-1/L1 plus VEGFR TKI


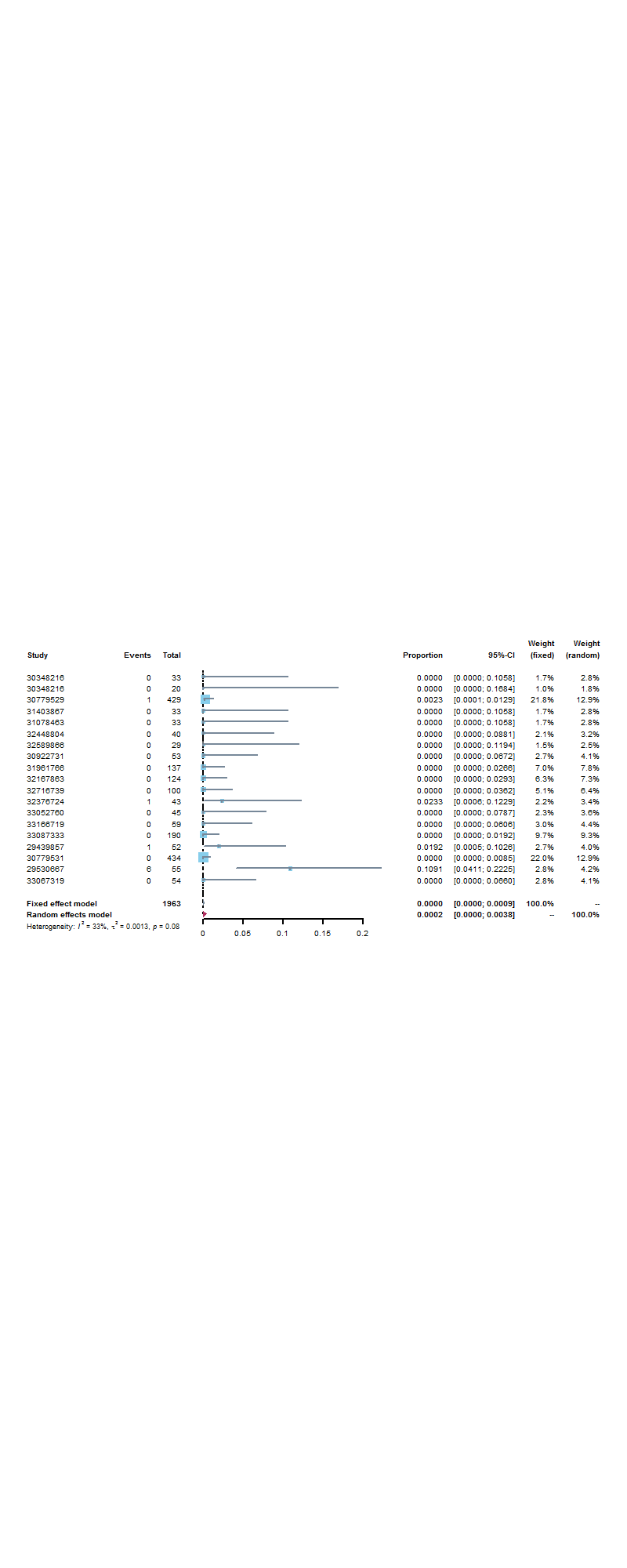


Forest plot of proportion of Urticaria with anti-PD-1 therapy


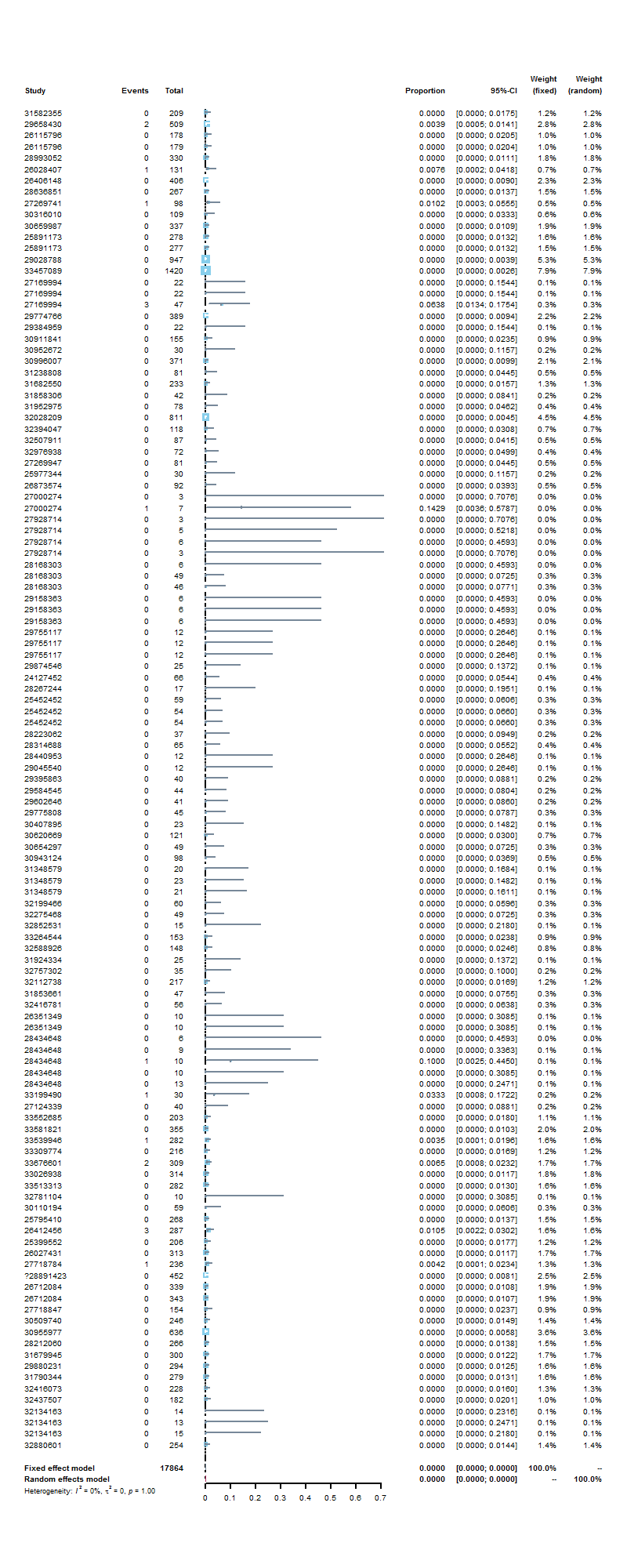


Forest plot of proportion of Urticaria with anti-CTLA-4 therapy


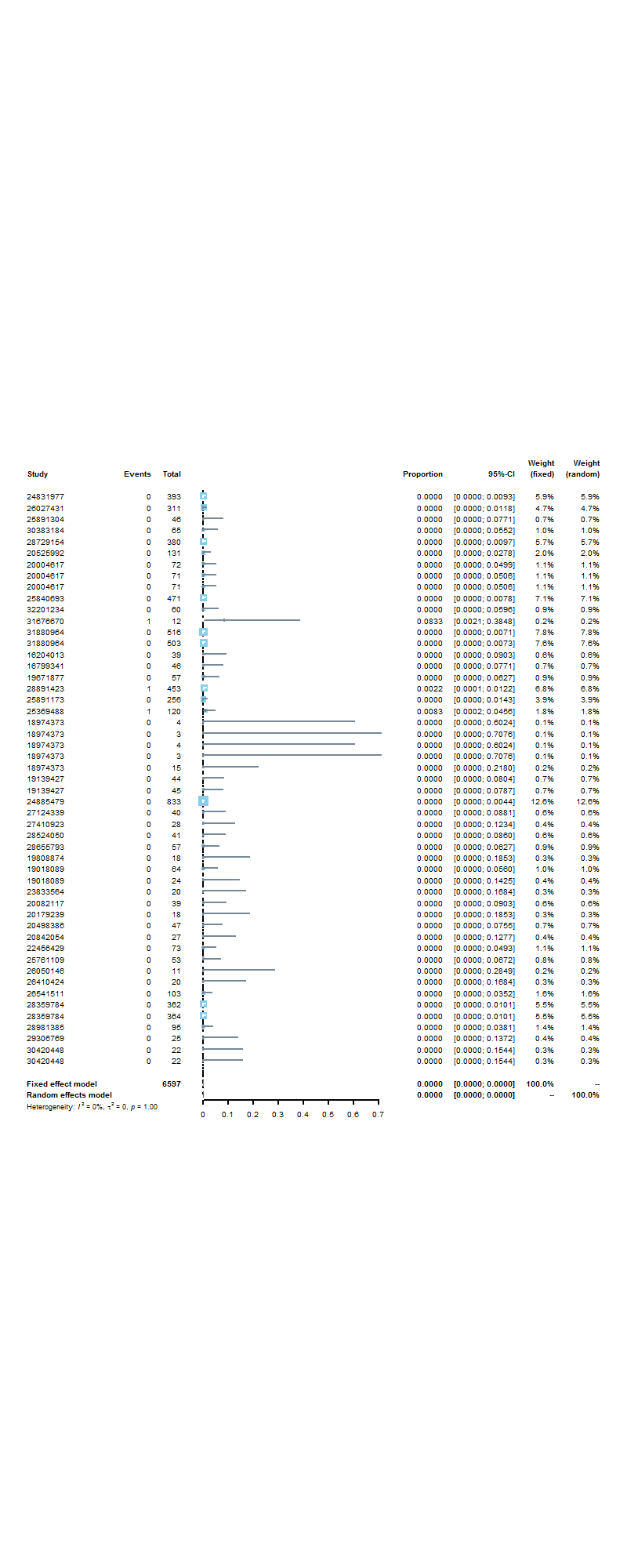


Forest plot of proportion of Urticaria with anti-PD-1/L1 plus anti-CTLA-4 therapy


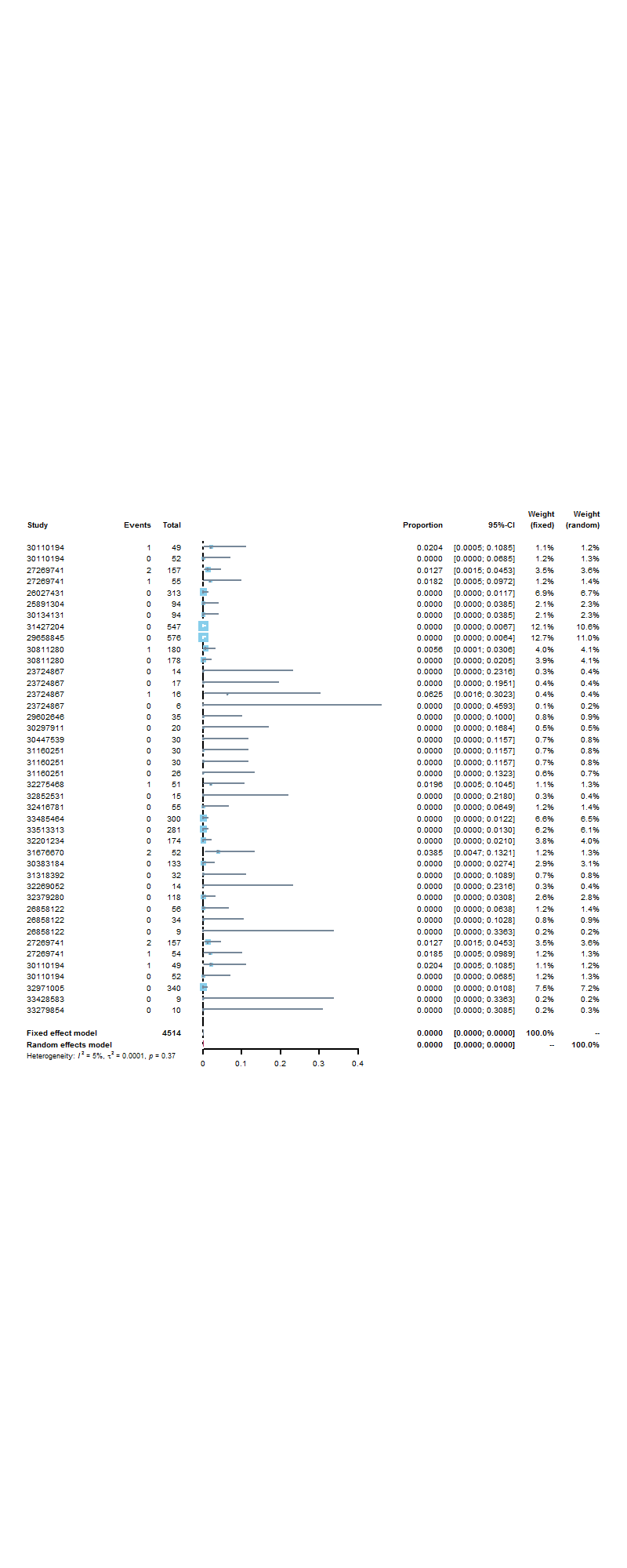


Forest plot of proportion of Urticaria with anti-CTLA-4 plus chemotherapy


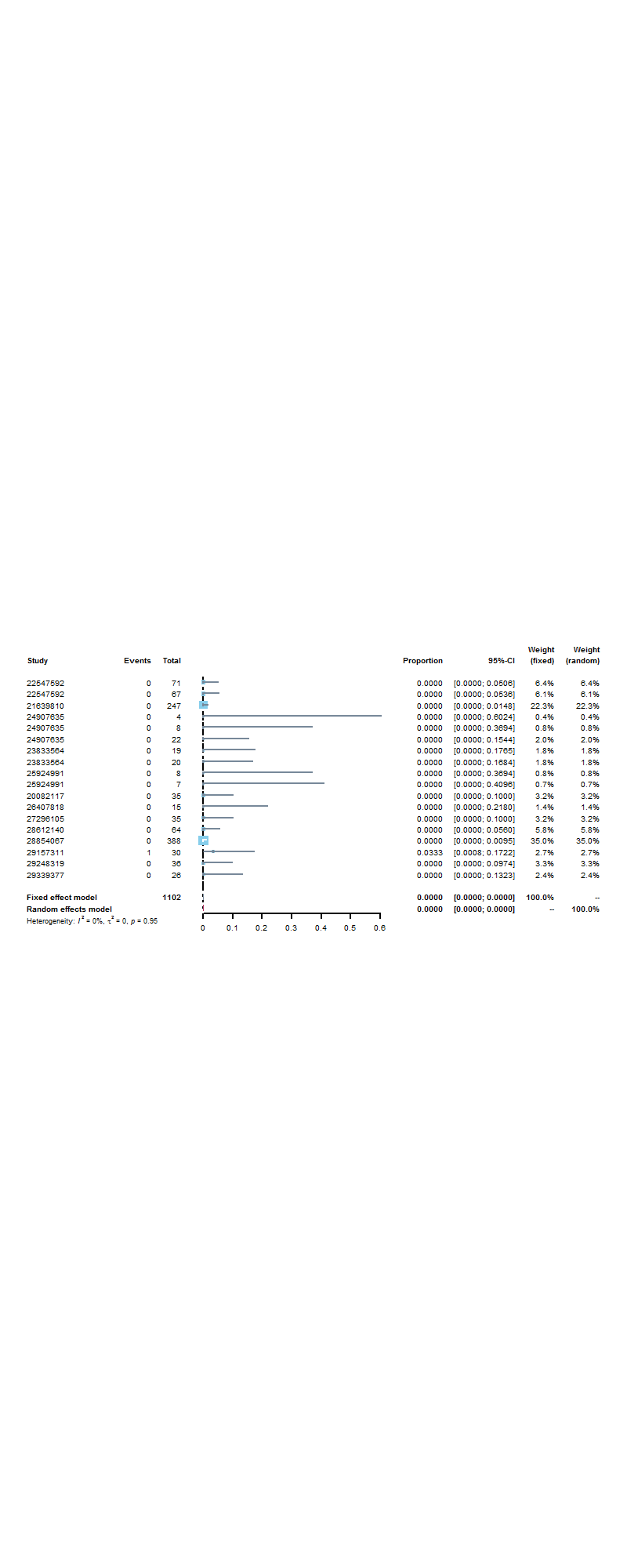


Forest plot of proportion of Urticaria with anti-PD-1/L1 plus EGFR TKI


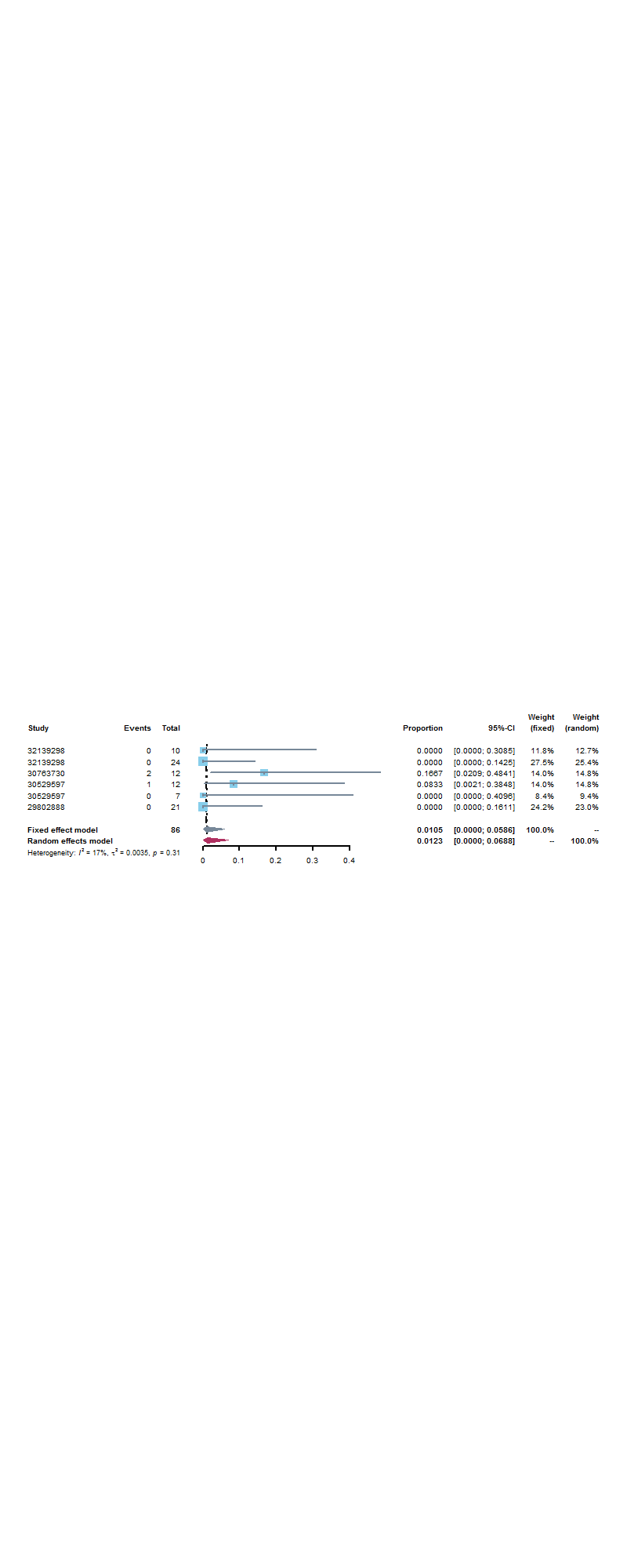


Forest plot of proportion of Urticaria with anti-PD-1/L1 plus VEGFR monoclonal antibody


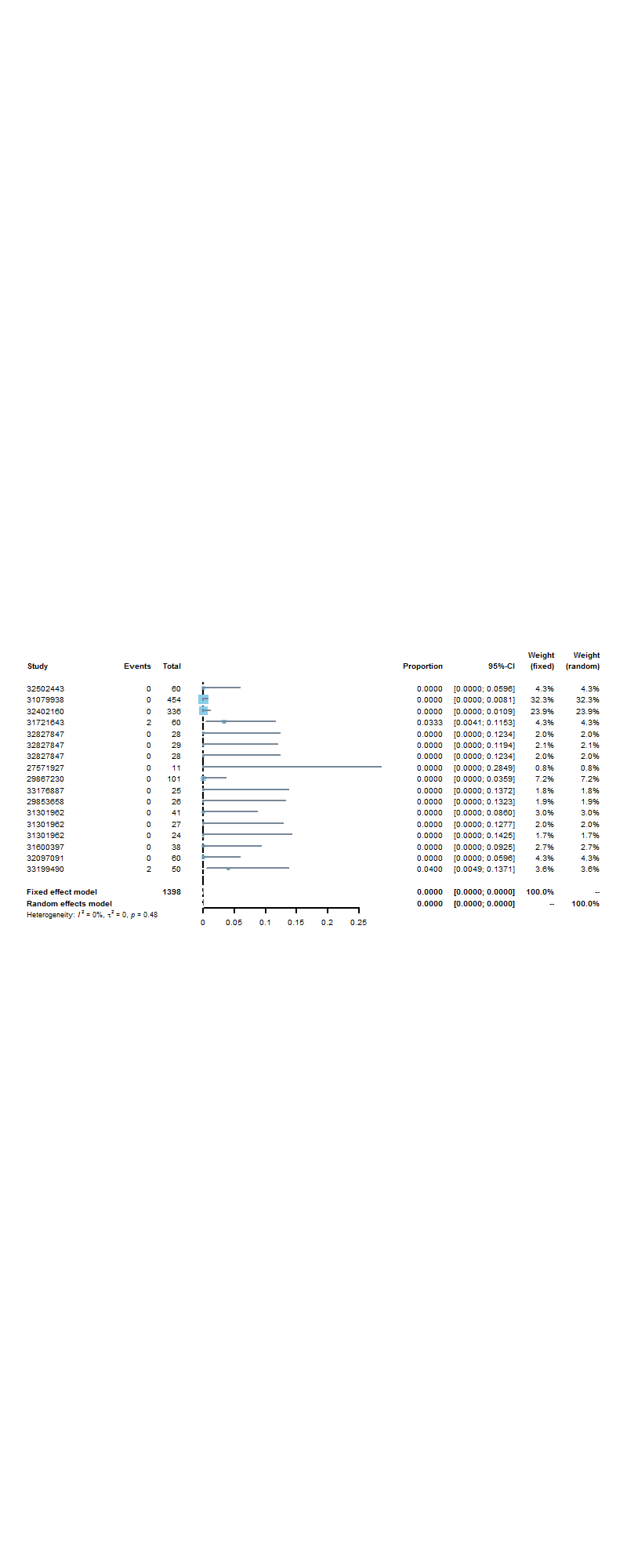


Forest plot of proportion of Urticaria with anti-PD-1/L1 plus VEGFR TKI


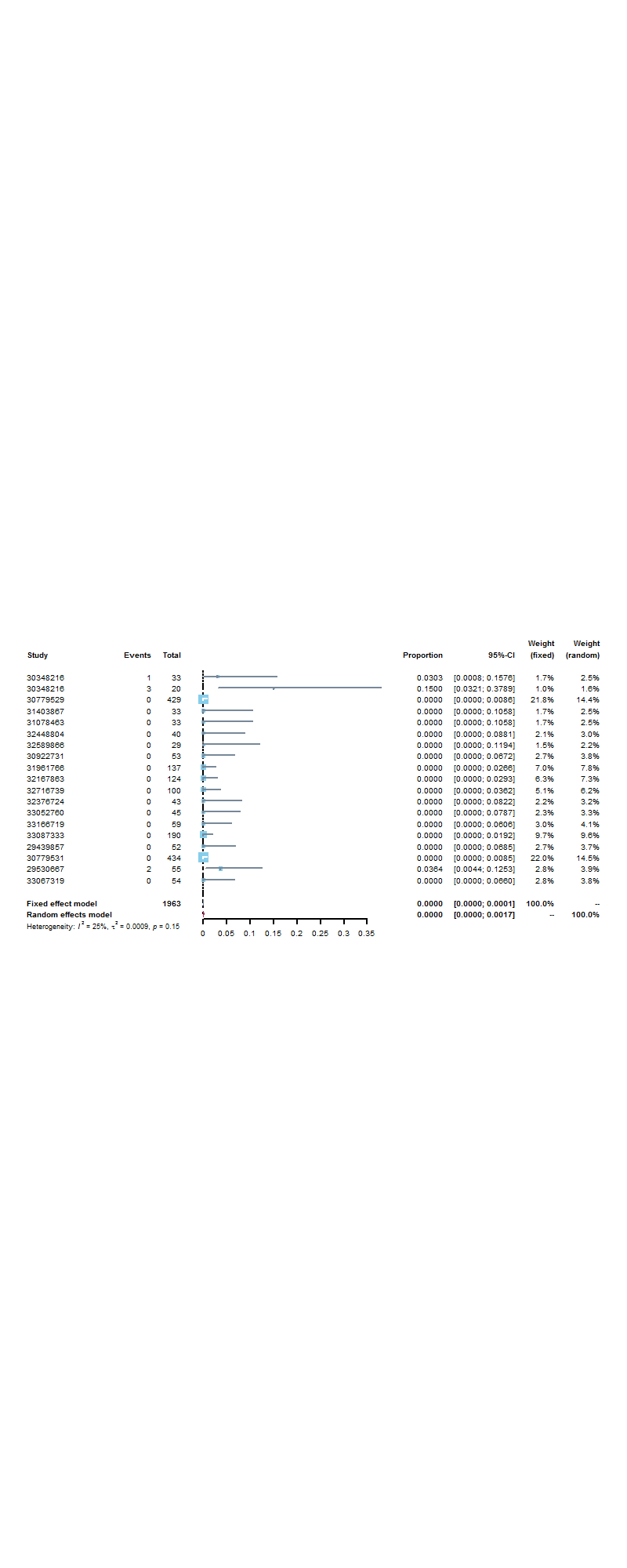


Forest plot of proportion of SJS with anti-PD-1 therapy


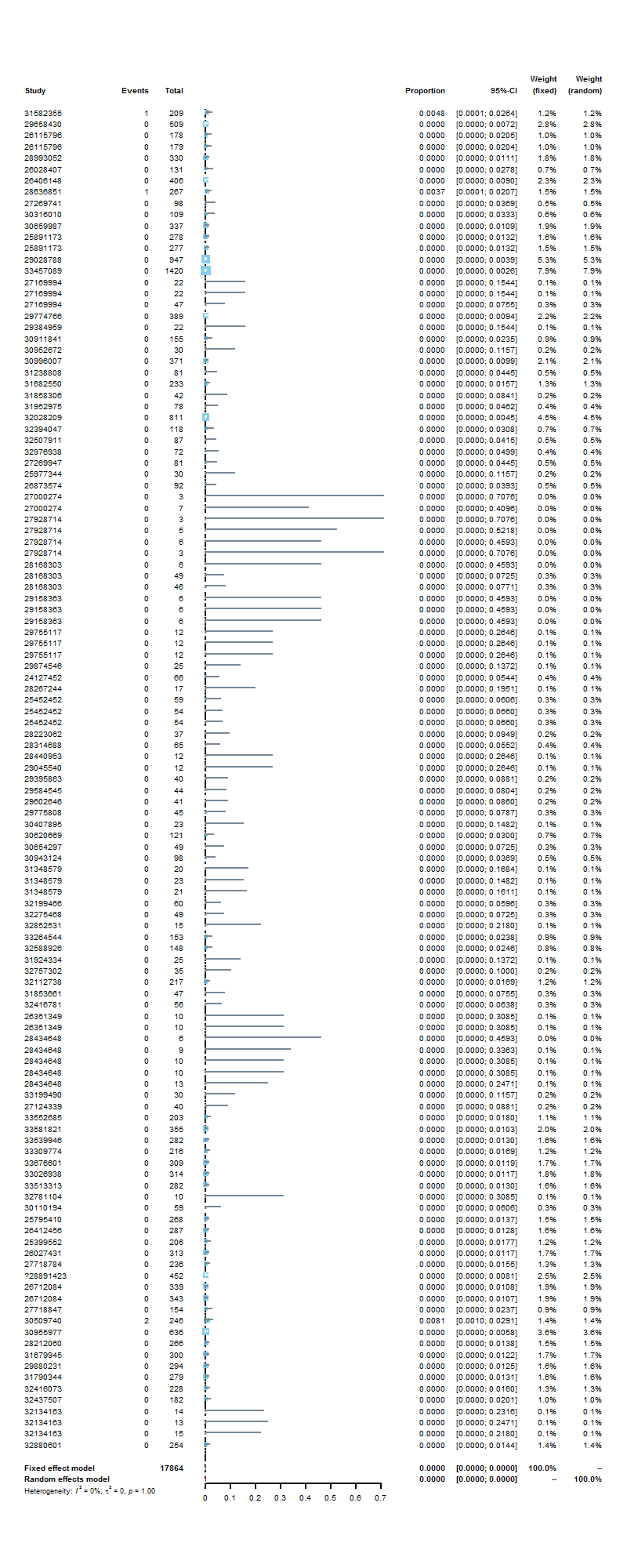


Forest plot of proportion of SJS with anti-PD-L1 therapy


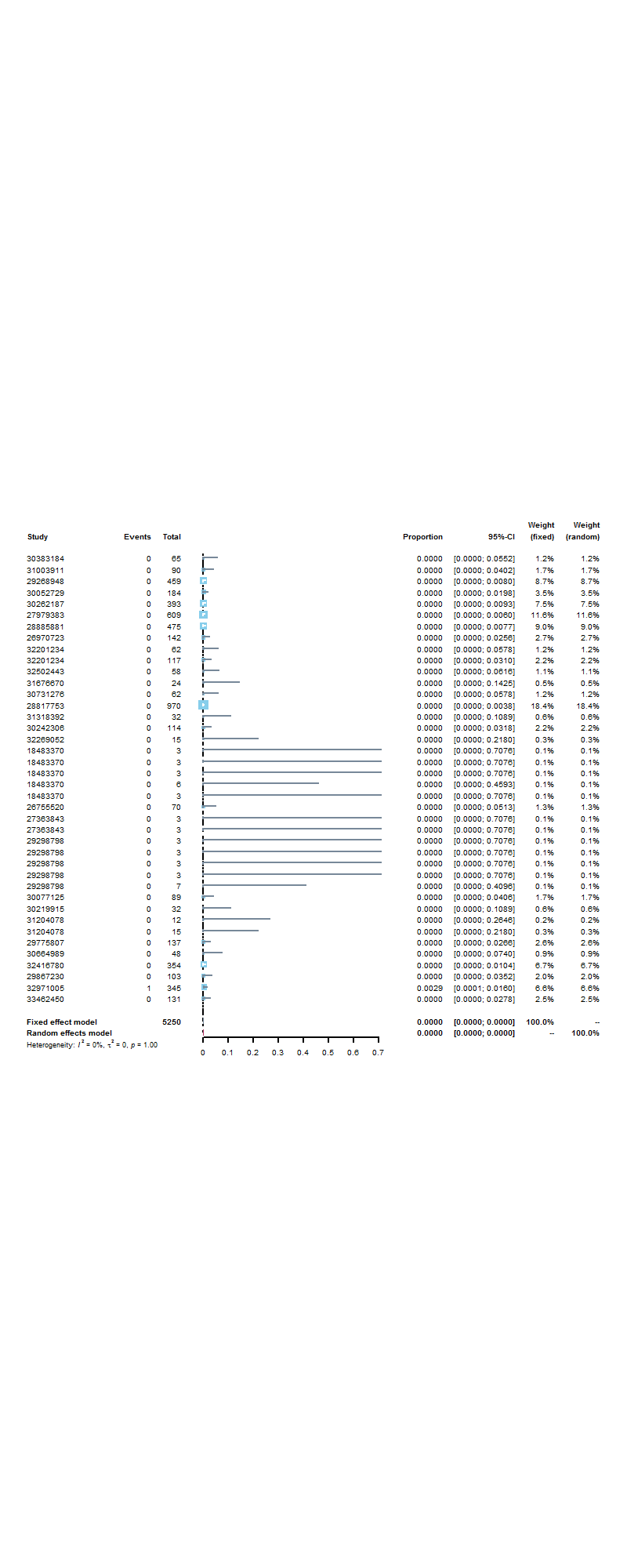


Forest plot of proportion of SJS with anti-CTLA-4 therapy


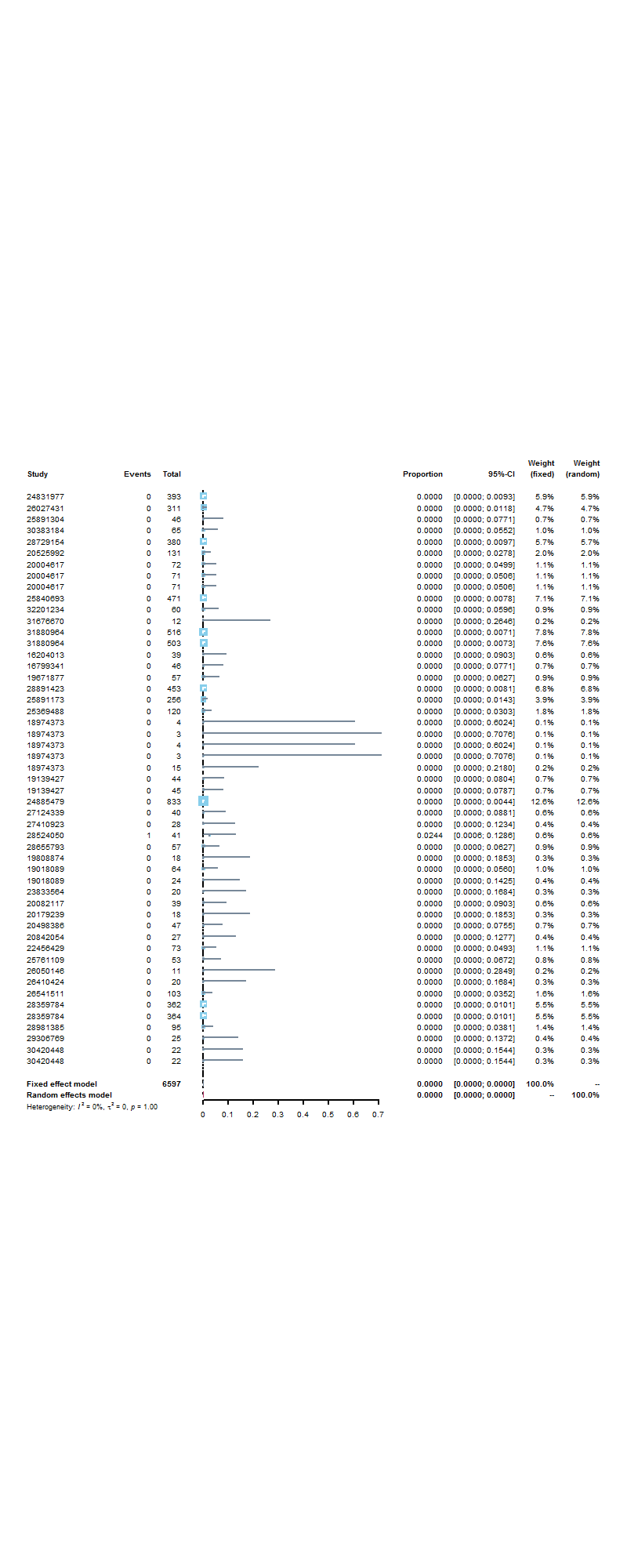


Forest plot of proportion of SJS with anti-PD-1/L1 plus anti-CTLA-4 therapy


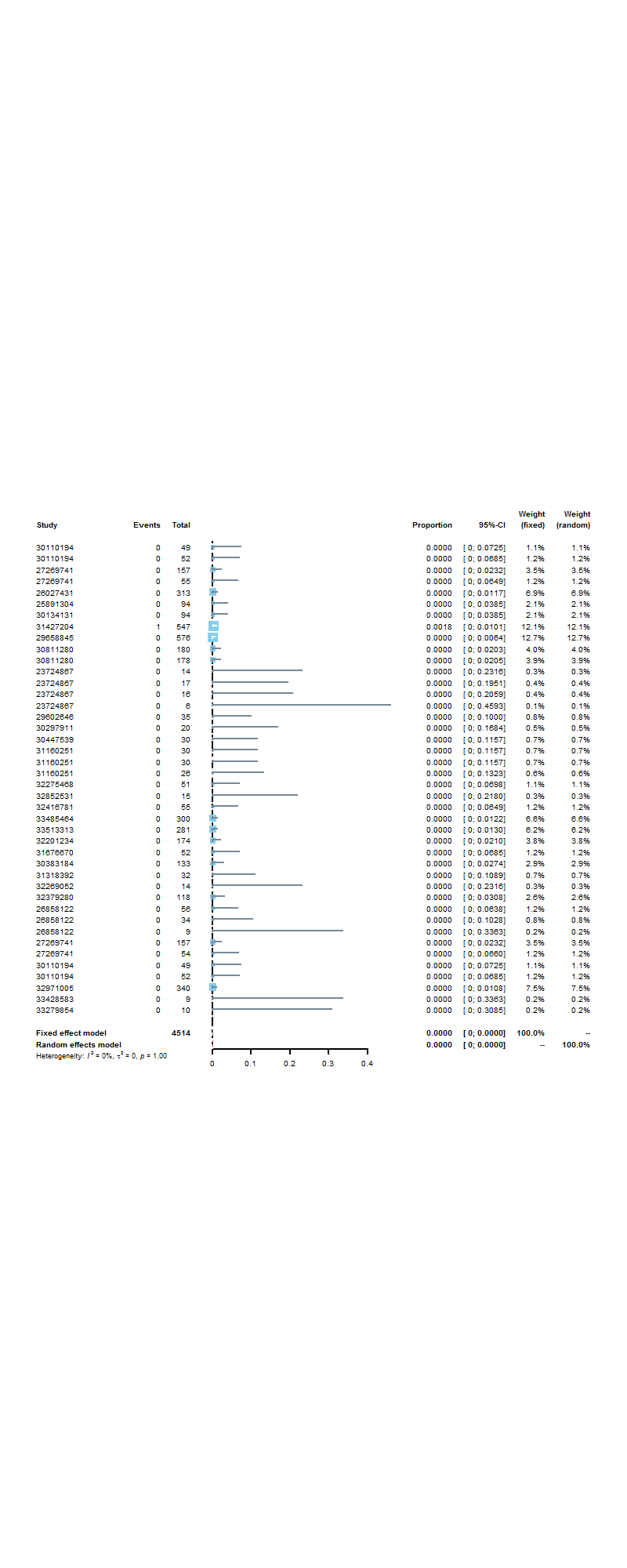


Forest plot of proportion of TEN with anti-PD-L1 therapy


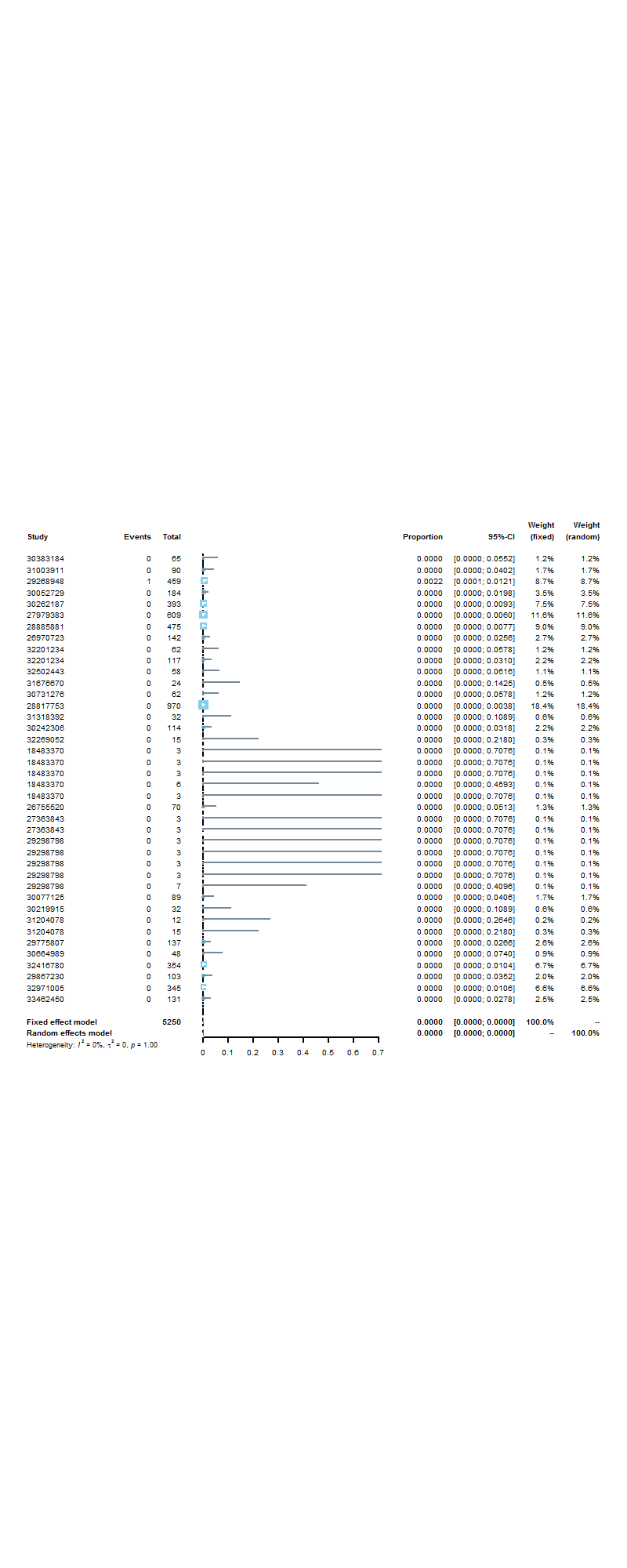


Forest plot of proportion of TEN with anti-PD-1 plus chemotherapy


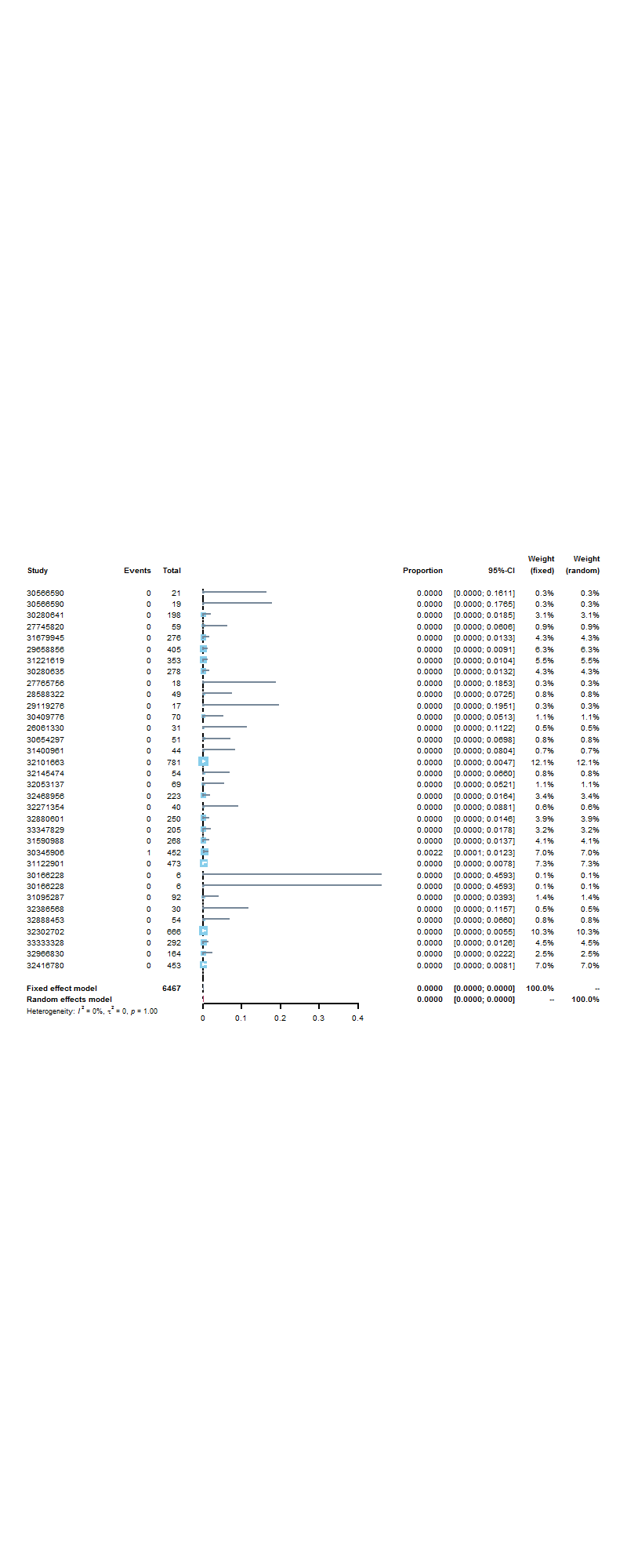


Forest plot of proportion of TEN with anti-PD-1 plus VEGFR TKI


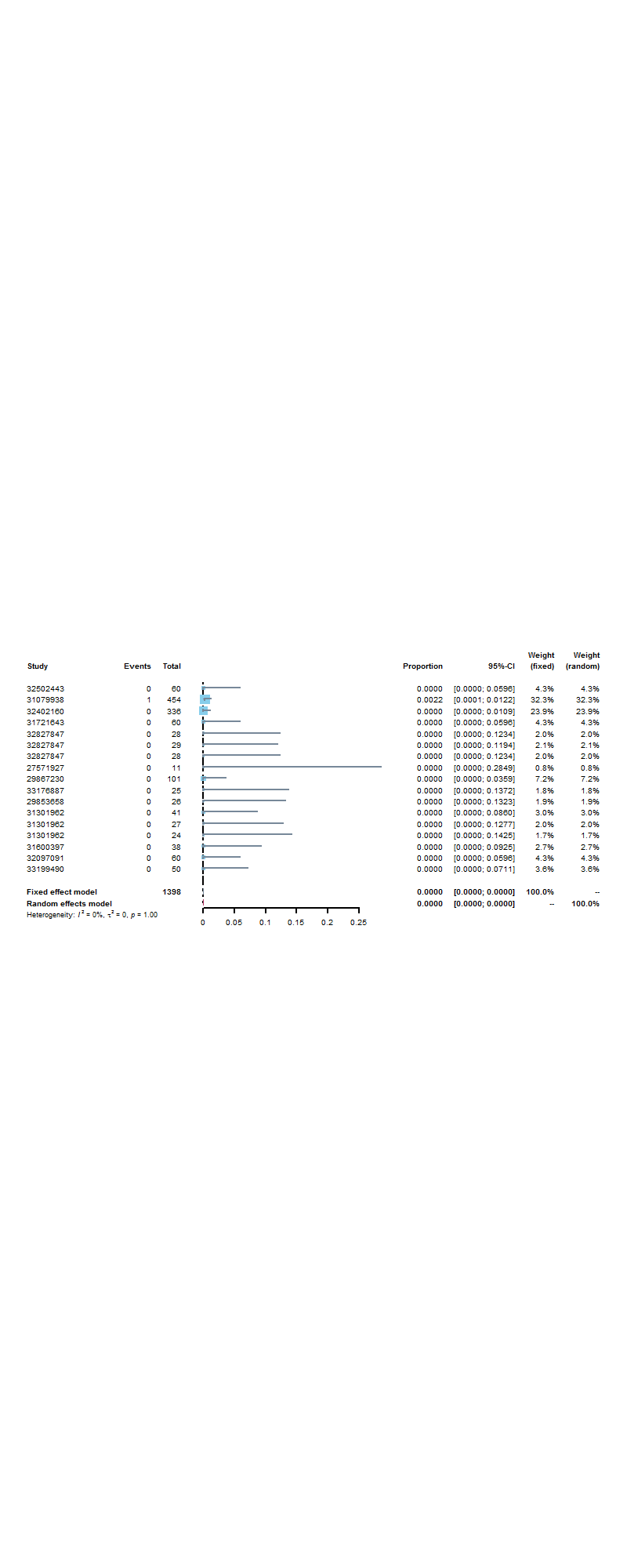

Supplement: Supplementary file 4 [file DataSheet2.docx]
