# Supplementary material for: Selected cutaneous adverse events in patients treated with ICI monotherapy and combination therapy: a retrospective pharmacovigilance study and meta-analysis
Source: Front Pharmacol. 2023 Jun 2;14:1076473. doi: 10.3389/fphar.2023.1076473 (PMC10272362; doi:10.3389/fphar.2023.1076473)

Appendix figure A : skin and mucous membrane of patients at the initial stage of treatment


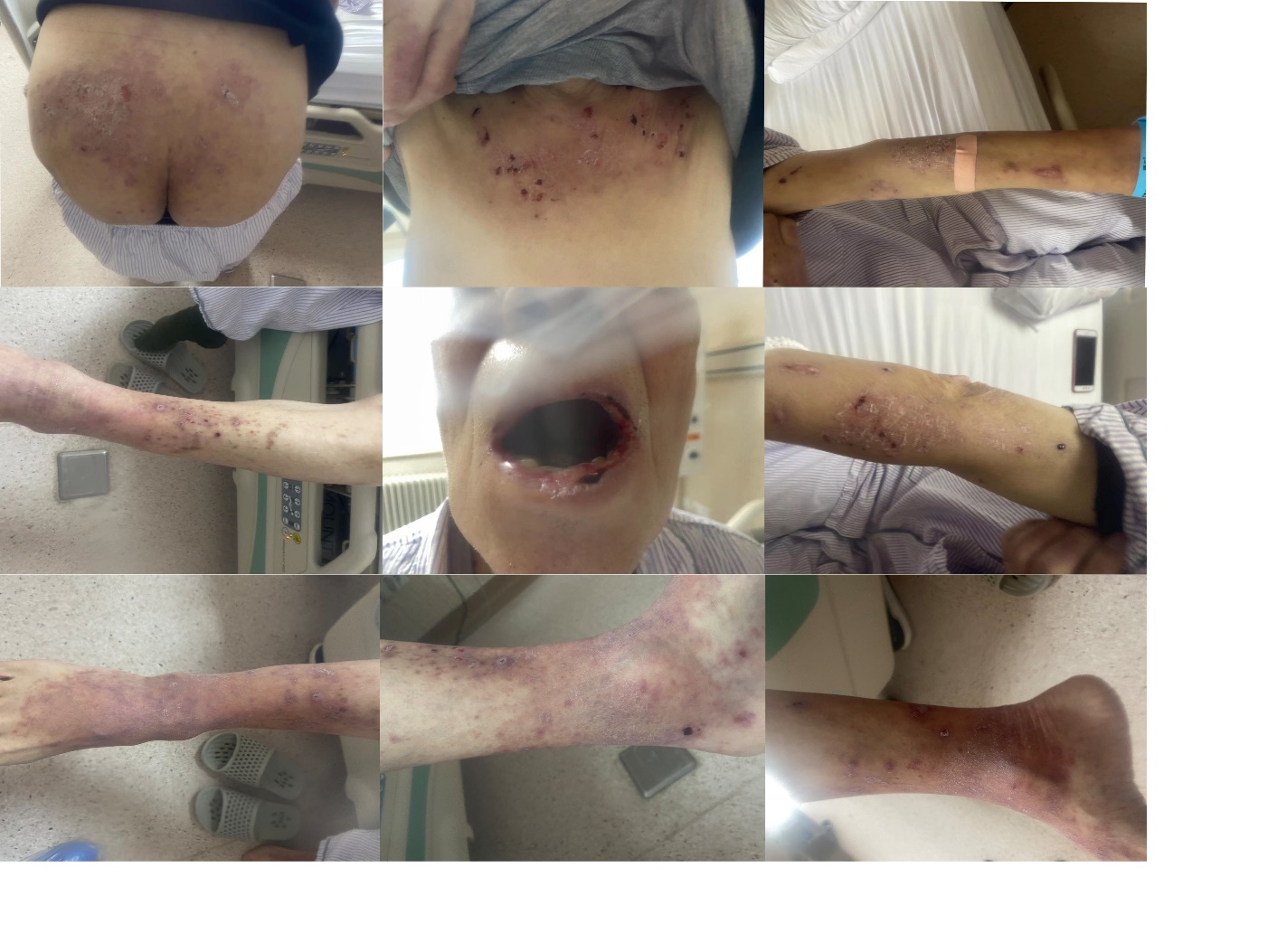


Appendix figure B : skin and mucous membrane of patients, 8 days after treatment


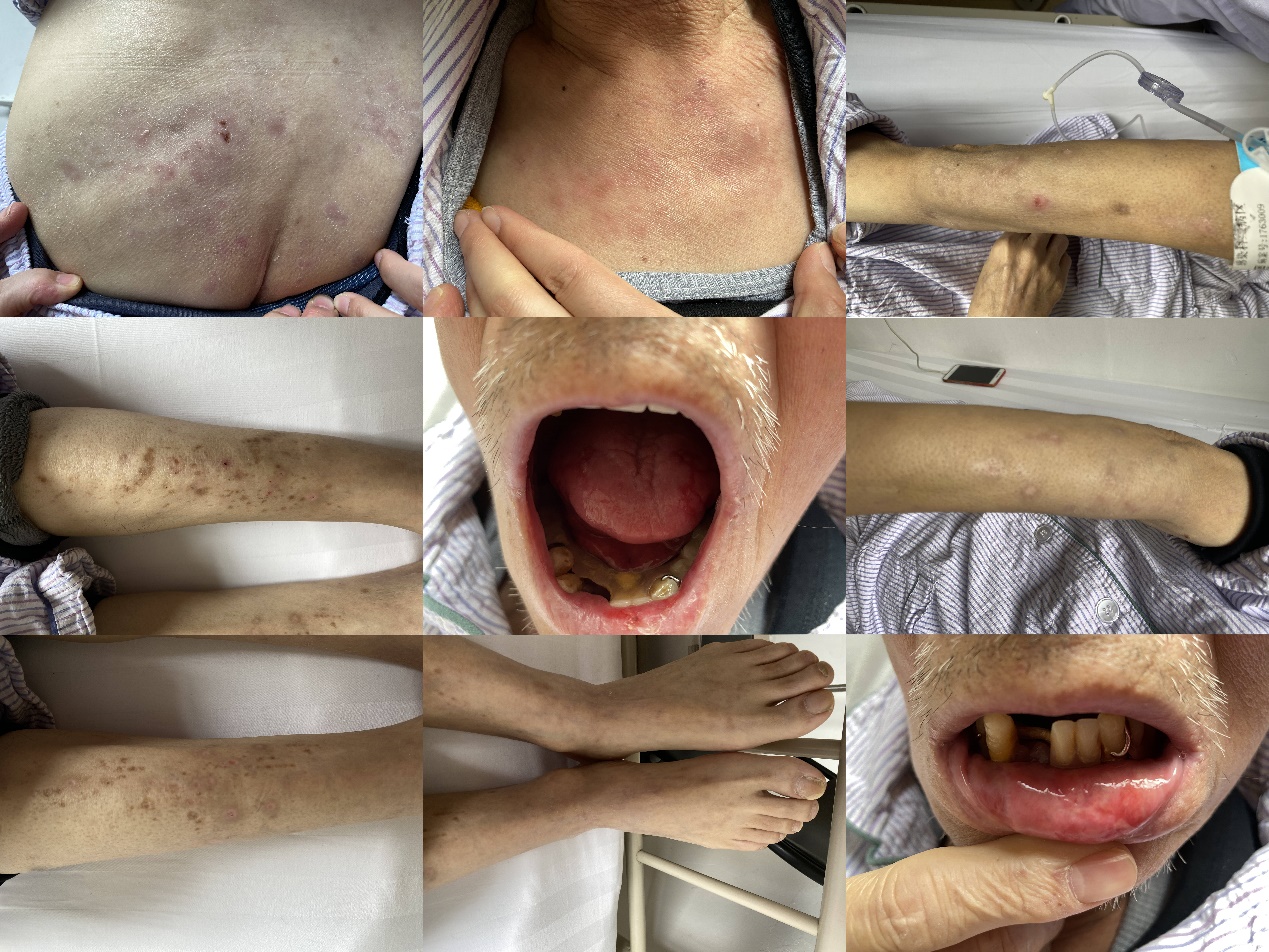

Supplement: Supplementary file 5 [file DataSheet1.docx]
